# New Polyketides and a Ferroptosis Inhibitor from the Marine-Derived Fungus *Diaporthe searlei* CS-HF-1

**DOI:** 10.3390/md23100402

**Published:** 2025-10-16

**Authors:** Jicheng Xiao, Peng Wu, Yan Zhang, Qi Lv, Yulang Chi, Wei Xu, Wenzhen Lin, Zhongbin Cheng

**Affiliations:** 1 Key Laboratory of Tropical Biological Resources of Ministry of Education, School of Pharmaceutical Sciences, Hainan University, Haikou 570228, China; extrayx@126.com (J.X.); pengwu0820@163.com (P.W.); 17373797879@163.com (Y.Z.); 15974358897@163.com (Q.L.); 2 Fujian Institute of Subtropical Botany, Xiamen 361006, China; 3 College of Oceanology and Food Science, Quanzhou Normal University, Quanzhou 362000, China; ylchi@qztc.edu.cn; 4 School of Basic Medical Sciences, YiChun University, Yichun 336000, China; xwkhj@163.com; 5 Xiamen Chenge Biotechnology Co., Ltd., Xiamen 361000, China

**Keywords:** *Diaporthe searlei*, alkaloids, polyketides, ferroptosis

## Abstract

As a driver of neurodegenerative disorders, ischemic injuries, and acute organ dysfunction, ferroptosis represents a therapeutic target, and its inhibition may provide novel therapies. In our ongoing efforts to discover ferroptosis inhibitors from fungal strains, chemical investigation of the strain *Diaporthe searlei* CS-HF-1 led to the isolation of four polyketide-derived alkaloids (**1**–**3** and **17**) and fourteen polyketides (**4**–**16** and **18**), including three new isoindolone derivatives (**1**–**3**), a new phthalide (**4**), a new butyrolactone derivative (**10**), and three new nonenolides (**11**–**13**). The structures were determined by comprehensive spectroscopic analysis. The structures of **1**, **2**, and **10** were confirmed by comparison of experimental and calculated ^13^C NMR chemical shifts. The absolute configurations of compounds **10**, **11**, and **14** were assigned by ECD calculations, while those of **12** and **13** were assigned based on their biogenetic relationship with **14**. Notably, compound **1** represents the first isoindolone featuring a primary amide group attached to the lactam nitrogen, while compound **2** is the first naturally occurring isoindolone dimer. These compounds were assessed for the anti-ferroptotic activity. As a result, asperlactone A (**15**) exhibited inhibition on RSL3-induced ferroptosis in HT22 cells with an EC_50_ of 11.3 ± 0.4 μM. Preliminary mechanistic study revealed that **15** attenuated lipid peroxidation, as evidenced by reduced MDA levels, elevated GSH content, and suppression of lipid radical generation. This study offers a new chemotype for the development of novel ferroptosis inhibitors.

## 1. Introduction

The genus *Diaporthe*, belonging to the family *Diaporthaceae* (order *Diaporthales*, class *Sordariomycetes*), is a large and taxonomically complex genus, with more than 1200 species recorded in the database Index Fungorum [1]. The genus was established in 1870, and its members have been discovered worldwide on a wide variety of terrestrial host plants and marine samples [1]. It is an outstanding genus of filamentous fungi that has been proven to be a prolific source of secondary metabolites [2,3]. In recent years, the metabolites of marine-derived *Diaporthe* species have attracted great attention, leading to the identification of a variety of new compounds [4,5,6,7,8,9,10,11,12,13,14,15,16,17,18,19,20,21,22,23,24,25], including polyketides (butyrolactone derivatives [4], oxygen-bridged cyclooctadiene derivatives [7], monomeric/dimeric/trimeric clavatol derivatives [6], chromone derivatives [9,16,17,21], mono or dimeric xanthones [11], highly oxygenated chloroazaphilone derivatives [19], anthraquinones [16], octaketides [18], and highly substituted phthalides [18]), monoterpenes (the rare thujanes diaporterpenes A–C [10] and acyclic monoterpenes diaporterpenes D–F [5]), sesquiterpenoids including drimane-type sesquiterpenoids [12,14,22], diterpenoids (diaporpenoid A and longidiacids A–B) [14,15], alkaloids (cytochalasins, chromeno[3,2-c]pyridines, and isoindolinones) [15,20], and meroterpenoids including chrodrimanins [12]. Their bioactivities mainly involve cytotoxic, anti-fibrotic, antifungal, antibacterial, antiviral, antioxidant, anti-inflammatory, anti-osteoclastogenesis, and enzyme inhibitory activities. Moreover, diaporthe H, a clavatol-dimer derivative, exhibited potent anti-fibrotic activity with an EC_50_ value of 3.5 μM while showing low cytotoxicity [6]. Mycoepoxydiene (A549, IC_50_ = 1.97 μM) and the cyclohexanone derivative 5,6-dihydroxy-3-(hydroxymethyl)-2,6-dimethylcyclohex-2-en-1-one (MDA-MB-231, IC_50_ = 2.54 μM) exhibited potent cytotoxicities [7]. Pestalotiopsone B, isolated from *Diaporthe* sp., displayed significant anti-influenza A virus activity against the A/Puerto Rico/8/34 (H1N1) strain (IC_50_ = 2.56 μM) [18]. Therefore, chemical study on *Diaporthe* spp. may yield novel/bioactive molecules.

Ferroptosis is an iron-dependent form of programmed cell death driven by lipid peroxidation [26]. It plays a significant role in pathologies, including neurodegenerative diseases, ischemic injuries, and cancer [27,28]. However, current inhibitors face clinical challenges such as poor metabolic stability and bioavailability. Given their structural diversity and biocompatibility, natural products are considered a promising source for developing next-generation ferroptosis inhibitors [29,30].

**Figure 1:**
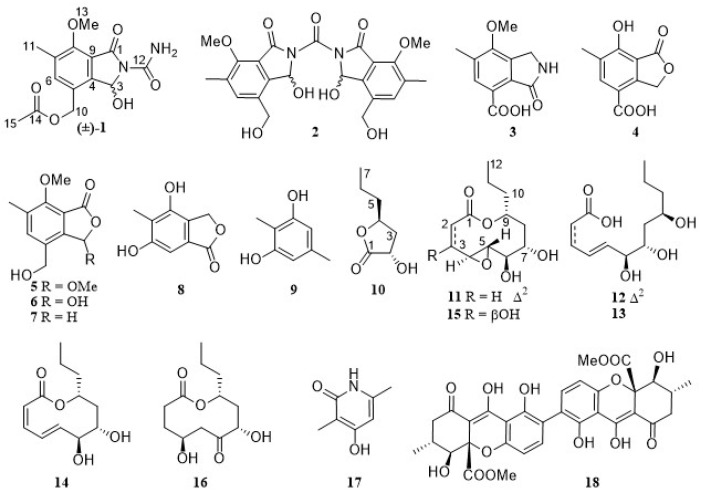
Structures of compounds **1**–**18** from the fungus *Diaporthe searlei* CS-HF-1.

In our ongoing search for bioactive metabolites from fungal strains [31,32,33,34,35,36], especially ferroptosis inhibitors [31,32,33,34], the ^1^H NMR spectrum of the EtOAc extract of the strain *Diaporthe searlei* CS-HF-1 indicated a rich secondary metabolite profile (Figure S1). A literature review revealed that only a potent antibacterial dimeric xanthone (**18**, secalonic acid A) was isolated from this species [37]. Thus, a systematic chemical study of this strain was performed and yielded 18 compounds (Figure 1). Herein, we present the isolation, structural elucidation, and anti-ferroptotic activity of these compounds.

## 2. Results and Discussion

### 2.1. Structural Elucidation

Compound **1** was obtained as a light-yellow oil. Its molecular formula, C_14_H_16_N_2_O_6_, was determined by the positive HRESIMS ion peak at *m*/*z* 331.0909 [M + Na]^+^ (calcd. 331.0901), indicating eight degrees of unsaturation. The ^1^H NMR spectrum (Table 1) showed signals for one methoxy (δ_H_ 3.93, s), one aromatic methyl (δ_H_ 2.28, s), one acetyl methyl (δ_H_ 2.06, s), one aromatic proton (δ_H_ 7.61, s), a pair of two coupled protons [δ_H_ 7.40 (1H, d, *J* = 10.0 Hz); 6.94 (1H, d, *J* = 10.0 Hz)], a broad singlet at δ_H_ 5.98 integrated for two protons, and two isolated and geminally coupled protons [δ_H_ 5.08 (1H, d, *J* = 12.7 Hz), 4.98 (1H, d, *J* = 12.7 Hz)] of an oxymethylene (which suggested spatial proximity to a methine stereocenter). The ^13^C NMR and HSQC spectra revealed a total of 14 carbons, which were assigned to three methyls (δ_C_ 61.7, 20.5, 15.2), including one methoxy, one oxymethylene carbon (δ_C_ 61.5), one oxymethine carbon (δ_C_ 81.5), six aromatic carbons for a pentasubstituted benzene ring (δ_C_ 156.0, 144.3, 137.4, 132.6, 126.4, 118.3), and three carbonyl carbons (δ_C_ 170.2, 166.3, and 156.9). With seven degrees of unsaturation assigned to three carbonyl groups and a benzene ring, the remaining one implied that compound **1** was bicyclic.

**Figure 2:**
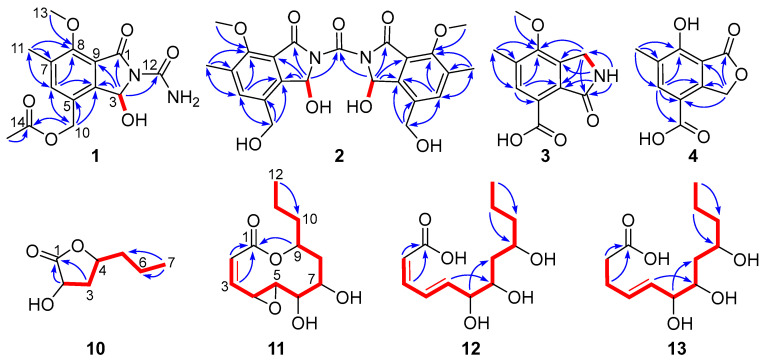
Key COSY (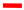) and HMBC (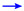) correlations of **1**–**4** and **10**–**13**.

The aforementioned data closely resembled the structural features of the co-isolated analogue phomotone (**6**) [38], containing an identical 4-methoxy-3-methylbenzyloxy moiety. The notable distinctions were the presence of one additional carbonyl carbon (δ_C_ 156.9), an acetyl group (δ_H_ 2.06; δ_C_ 20.5, 170.2), and the upfield-shifted hydroxymethine carbon (**1**: δ_C_ 81.5; **6**: δ_C_ 108.7). The chemical shifts of the hydroxylated methine (δ_H_ 6.94; δ_C_ 81.5) combined with the *N*-containing molecular formula indicated the replacement of the lactone moiety in **6** by a lactam unit in **1**, suggesting an isoindolone derivative. In addition, the HMBC correlation from the oxymethylene protons H_2_-10 (δ_H_ 5.08, 4.98) to the acetyl carbonyl carbon C-14 (δ_C_ 170.2) linked the acetoxy group to C-10 (Figure 2). The residual elemental composition, after accounting for the aforementioned functionalities, was consistent with a primary amide group (-CONH_2_). The deduction was confirmed by the characteristic broad singlet for protons of primary amine at δ_H_ 5.98 (2H, br s) and the HMBC correlation from the hydroxymethine proton at δ_H_ 6.94 to the primary amine carbonyl carbon (δ_C_ 156.9). The structure of **1** was confirmed by comprehensive 2D NMR analysis (Figure 2). Compound **1**, a polyketide-derived alkaloid, was an isoindolone derivative featuring a primary amine unit attached to the amide nitrogen atom.

**Figure 3:**
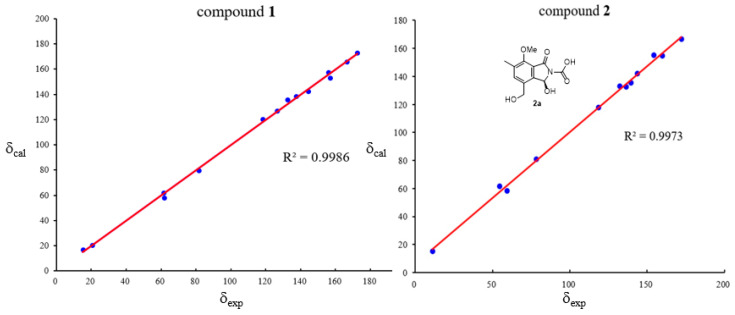
Regression analysis of experimental and calculated ^13^C NMR chemical shifts (ppm) of **1** and **2** (a simplified structure **2a** was used).

To confirm its structure, we conducted DFT calculations of the ^13^C NMR chemical shifts employing the GIAO method at the mPW1PW91/6-311+G(d,p) level using the conductor polarizable calculation model (CPCM) with dimethyl sulfoxide (DMSO) as solvent. The results revealed an excellent linear correlation (*R*^2^ = 0.9986) between the calculated and experimental chemical shifts (Figure 3 and Table S1), thereby providing strong evidence for the assigned structure. Compound **1** was optically inactive and gave a flat CD spectrum (200–400 nm), which indicated that **1** was racemic. Subsequent attempts to resolve the enantiomers of **1** on a Phenomenex Lux Cellulose-2 chiral column with the mobile phase CH_3_CN/H_2_O or *n*-hexane/isopropanol failed. Thus, compound **1** was characterized as a racemate and assigned the trivial name (±)-diasearamide A.

Compound **2** was assigned the molecular formula C_23_H_24_N_2_O_9_ based on positive HRESIMS data (*m*/*z* 495.1398 [M + Na]^+^, calcd. 495.1374) and negative HRESIMS data (*m*/*z* 471.1400 [M − H]^−^, calcd. 471.1409). The ^1^H NMR spectrum displayed signals for one aromatic singlet (δ_H_ 7.62, s), one aromatic methyl (δ_H_ 2.28, s), one methoxy (δ_H_ 3.91, s), two coupled protons [δ_H_ 7.71 (1H, d, *J* = 9.3 Hz); 6.93 (1H, d, *J* = 9.3 Hz)], one oxygenated methylene (δ_H_ 4.51, s), and one broad singlet for one proton at δ_H_ 5.30. The ^13^C NMR spectrum revealed only 12 carbons (Table 1), which were assigned with the aid of the HSQC spectrum to two methyl carbons (δ_C_ 61.6, 15.2), one oxymethylene carbon (δ_C_ 58.4), one oxymethine carbon (δ_C_ 80.9), six aromatic carbons for a pentasubstituted benzene ring (δ_C_ 154.8, 142.2, 135.5, 132.8, 132.4, 117.8), and two carbonyl carbons (δ_C_ 166.6, and 155.2). The observation of 12 carbons, inconsistent with the molecular formula C_23_H_24_N_2_O_9_, suggested a dimeric architecture comprising two identical C-11 subunits linked by a shared carbon atom. A detailed comparison of the NMR data of **1** and **2** indicated that the monomeric unit of **2** corresponded to a derivative of **1** lacking the acetyl and the amide groups. The two C_11_ subunits were linked by a carbonyl carbon based on the HMBC correlation from the oxymethine proton (δ_H_ 6.93) to the remaining carbonyl carbon at δ_C_ 155.2. The structure of **2** was secured by detailed 2D NMR analysis (Figure 2). To further confirm the structure, we performed computational predictions of the NMR chemical shifts of **2** with a simplified model molecule **2a** using the GIAO method at the mPW1PW91/6-311+G(d,p) level with the conductor polarizable calculation model (CPCM) in DMSO. The calculated NMR chemical shifts (Figure 3 and Table S2) for **2a** were in good agreement with the experimental ^13^C NMR data of **2** (*R*^2^ = 0.9979), which further supported the structure of **2**. Since the monomers of the two fragments could have either the same or opposite absolute configurations, the chiral centers are depicted with wavy bonds. Compound **2** was named diasearamide B.

The molecular formula of diasearamide C (**3**) was determined to be C_11_H_11_NO_4_ based on analysis of the HRESIMS (*m*/*z* 220.0614 [M − H]^−^, calcd. 220.0615) and ^13^C NMR data. The ^1^H NMR spectrum exhibited signals for one methoxy (δ_H_ 3.80), one aromatic methyl (δ_H_ 2.32), one aromatic singlet (δ_H_ 7.66), one oxymethylene (δ_H_ 4.39), and one broad singlet assigned to an amide proton (δ_H_ 8.51). The ^13^C NMR and HSQC spectra indicated the presence of six aromatic carbons for a pentasubstituted benzene ring (δ_C_ 160.1, 143.5, 132.3, 128.1, 127.6, 121.8), one carbonyl carbon (δ_C_ 168.9), one methylene carbon (δ_C_ 45.4), and two methyl carbons (δ_C_ 61.3, 16.1), including one methoxy. These structural features were very similar to those of **1** and **2**, indicating an isoindolone derivative with a similar carbon skeleton. The HMBC correlations from the methoxy protons to C-8 (δ_C_ 160.1) and from the aromatic methyl protons to C-6 (δ_C_ 127.6), C-7 (δ_C_ 132.3), C-8, and C-9 (δ_C_ 128.1) indicated these two groups were located at the same positions as those in **1** and **2**. The structure of **3** was further determined by detailed 2D NMR analysis (Figure 2). Specifically, the HMBC correlations from the amide proton at δ_H_ 8.51 (H-2, br s) to C-3 (δ_C_ 168.9), C-4 (δ_C_ 143.5), and C-9 (δ_C_ 128.1) and from H_2_-1 (δ_H_ 4.39) to C-8 and C-7 positioned the methylene group at C-1 and the carbonyl carbon at C-3. Based on the molecular formula, the presence of a carboxyl group (δ_C_ 166.4) was required, and it must be attached to the remaining non-hydrogenated aromatic carbon C-5 (δ_C_ 121.8). Thus, the structure of **3** was determined as depicted and named diasearamide C.

Diasearlide acid (**4**), a light-yellow oil, was assigned the molecular formula C_10_H_8_O_5_ as determined by the negative HRESIMS ion at *m*/*z* 207.0296 ([M − H]^−^, calcd. 207.0299), suggesting seven degrees of unsaturation. The ^1^H and ^13^C NMR spectrum revealed the presence of one pentasubstituted benzene ring (δ_H_ 7.75; δ_C_ 165.9, 148.8, 130.2, 128.1, 115.1, 108.0), one methylene (δ_H_ 5.40; δ_C_ 70.4), one aromatic methyl (δ_H_ 2.23; δ_C_ 15.6), and two carbonyl groups (δ_C_ 171.3, 170.1). The benzene ring and the two carbonyl carbons accounted for six degrees of unsaturation; the remaining one suggested **4** to be bicyclic. These data indicated a phthalide derivative, structurally similar to the co-isolated convolvulol (**7**) [39]. The differences were the absence of the methoxy (δ_H_ 4.02) and the hydroxymethyl groups (δ_H_ 4.68). The HMBC correlations from the aromatic proton (δ_H_ 7.75) to C-4 (δ_C_ 148.8), C-5 (δ_C_ 115.1), and the carbonyl group (δ_C_ 170.1) indicated the replacement of the hydroxymethyl group in **7** by a carboxyl group. Based on the molecular formula, the methoxy group in **7** was replaced by a hydroxy group. Detailed analysis of the 2D NMR data confirmed the structure of **4** (Figure 2).

**Figure 4:**
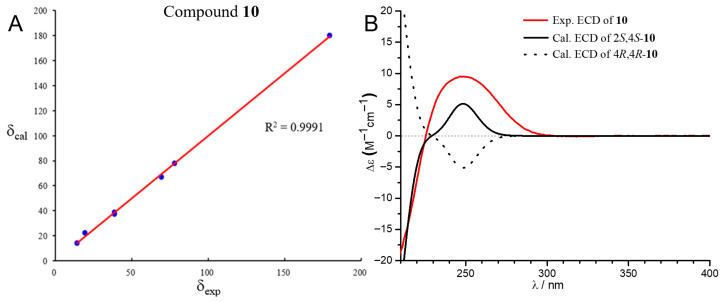
(**A**) Regression analysis of experimental and calculated ^13^C NMR chemical values (MeOH) of **10**; (**B**) experimental and calculated Electronic Circular Dichroism (ECD) in MeOH of **10**.

The molecular formula of compound **10** was determined to be C_7_H_12_O_3_ by HRESIMS (*m*/*z* 143.0710 [M − H]^−^, calcd. 143.0714), establishing an index of hydrogen deficiency of two. The ^1^H NMR spectrum of **10** showed signals for two oxygenated protons [δ_H_ 4.53 (1H, dd, *J* = 10.7, 8.8 Hz); 4.40 (1H, m)], a methyl triplet [δ_H_ 0.98 (3H, t, *J* = 7.2 Hz)], and several aliphatic protons. The ^13^C NMR and HSQC spectra indicated the presence of a carbonyl carbon (δ_C_ 179.4), two oxymethine carbons (δ_C_ 78.1, 69.5), three methylene carbons (δ_C_ 38.7, 38.6, 19.4), and a methyl carbon (δ_C_ 14.1). Since one of the two degrees of unsaturation was accounted for by one carbonyl carbon, the remaining one indicated that **10** was monocyclic. The gross structure was further established by HMBC and ^1^H-^1^H COSY correlations. Specifically, the ^1^H-^1^H COSY relationship of H-2 (δ_H_ 4.53)/H-3a (δ_H_ 2.66)/H-4 (δ_H_ 4.40)/H-5 (δ_H_ 1.63)/H_2_-6 (δ_H_ 1.47)/H_3_-7 (δ_H_ 0.98) defined a six-carbon spin system (CH-2/CH_2_-3/CH-4/CH_2_-5/CH_2_-6/CH_3_-7), the HMBC correlations from H-2 and H-3 to C-1 indicated the C_6_ unit was linked to the carbonyl carbon (δ_C_ 179.4). The remaining degree of unsaturation required that C-4 must be connected to the carbonyl carbon C-1 via one O-atom to form a lactone moiety. Since there was no NOESY correlation between H-2 and H-4, the relative configuration of **10** was assigned to be 2S* and 4S*, respectively. The structure of 10 was confirmed by matching its experimental ^13^C NMR data with the calculated shifts (*R*^2^ = 0.9991), demonstrating excellent consistency (Figure 4, Table S3). Further ECD calculation defined the absolute configuration of the two chiral centers to be 2*S* and 4*S*, respectively (Figure 4). Thus, the structure of compound 10 was determined to be (2*S*,4*S*)-2-hydroxy-4-propylbutyrolactone.

**Figure 5:**
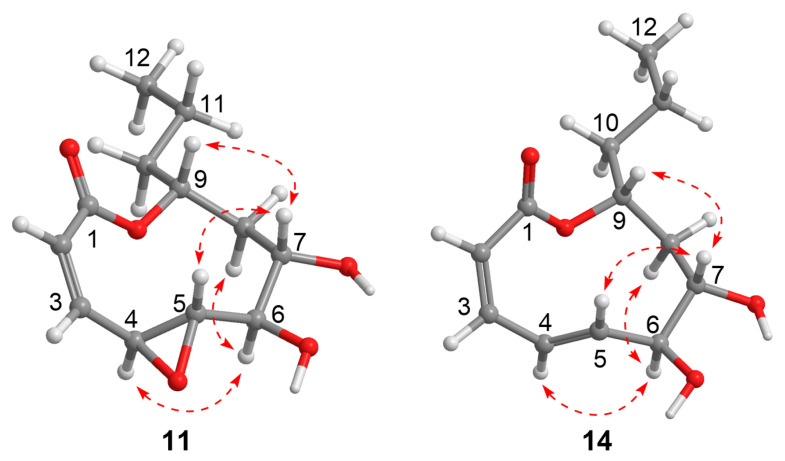
The NOESY correlations of compounds **11** and **14**.

**Figure 6:**
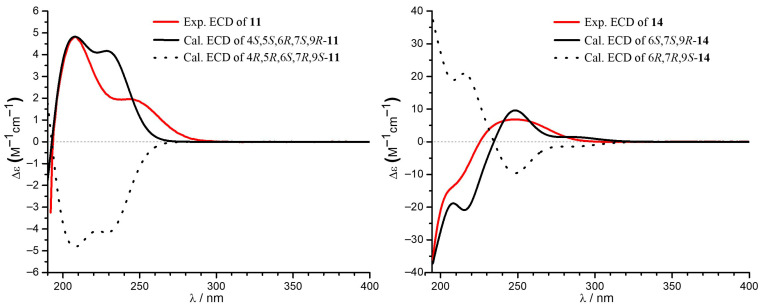
Experimental and Calculated Electronic Circular Dichroism (ECD) Spectra of **11** and **14** in MeOH.

The molecular formula of **11** was established as C_12_H_18_O_5_ based on the HRESIMS (*m*/*z* 241.1065 [M − H]^−^, calcd. 241.1081), indicating the presence of one additional oxygen atom compared to the co-isolated xylarolide (**14**) [40]. The ^1^H NMR spectrum showed signals for two coupled olefinic protons of a *cis*-double bond [δ_H_ 6.52 (d, *J* = 11.0 Hz), 5.96 (d, *J* = 11.0, 1.5 Hz)], five oxygenated protons (δ_H_ 4.88, 3.75, 3.58, 3.03, 2.60), a methyl triplet [δ_H_ 0.95 (3H, s, *J* = 7.2 Hz)], and several aliphatic protons. The ^13^C NMR and HSQC spectra revealed 12 carbons, including one carbonyl carbon (δ_C_ 166.6), two olefinic carbons (δ_C_ 142.6, 126.0), five oxymethine carbons (δ_C_ 79.0, 76.3, 73.7, 62.6, 57.5), three methylene carbons (δ_C_ 41.6, 38.5, 19.3), and one methyl carbon (δ_C_ 14.3). These data (Table 2) showed high similarity to the co-isolated analogue xylarolide (**14**) except for the presence of two additional oxygenated carbon signals (δ_C_ 62.6 and 57.5) in **11** instead of signals for Δ^3^ in **14**. This indicated that **11** was a 3,4-epoxy derivative of **14**. The location of the epoxy ring was confirmed by the COSY relationships of H-2 (δ_H_ 5.96)/H-3 (δ_H_ 6.52)/H-4 (δ_H_ 3.75)/H-5 (δ_H_ 2.60), along with HMBC correlations from H-2 (δ_H_ 5.96) to C-4 (δ_C_ 57.5) and from H-3 (δ_H_ 6.52) to C-5 (δ_C_ 62.6) (Figure 2). The relative configuration was determined by a NOESY experiment (Figure 5). Specifically, the correlations from H-6 to H-4 and H-8b, from H-7 to H-5 and H-9, in association with the coupling constant *J*_4,5_ (2.0 Hz), indicated that the protons H-5, H-7, and H-9 were cofacial and H-4 and H-6 were in the opposite orientation. To determine the absolute configuration of **11**, ECD calculation of 4*S*, 5*S*, 6*R*, 7*S*, 9*R*-**11** was conducted using B3LYP/6-31+G(d,p) optimized geometries at the B3LYP/6-31+G(d,p) level with the solvation model based on density (SMD) in MeOH. The experimental ECD spectrum of **11** showed a similar ECD curve to the calculated curve for 4*S*, 5*S*, 6*R*, 7*S*, 9*R*-**11** at around 220 nm (Figure 6). Compound **11** was named diasearolide A.

Compound **12** had the molecular formula C_12_H_20_O_5_ as determined by the HRESIMS (*m*/*z* 243.1236 [M − H]^−^, calcd. 243.1238), indicating three degrees of unsaturation. The NMR data indicated the presence of one carbonyl carbon (δ_C_ 169.7), one *trans* and one *cis* double bonds [δ_H_ 7.54 (1H, dd, *J* = 15.4, 11.5 Hz), 6.68 (1H, dd, *J* = 11.5, 11.4 Hz), 6.13 (1H, dd, *J* = 15.4, 6.4 Hz), 5.65 (1H, dd, *J* = 11.4 Hz); δ_C_ 145.4, 144.2, 128.8, 118.9], three oxymethines (δ_H_ 4.06, 3.82, 3.80; δ_C_ 76.7, 72.3, 68.8), three methylenes [δ_H_ 1.51 (2H, m), 1.43 (2H, m), 1.44 (1H, m), 1.37 (1H, m); δ_C_ 41.5, 41.2, 19.9), and one methyl [δ_H_ 0.93 (3H, t, *J* = 6.9 Hz); δ_C_ 14.4)]. These data closely resembled those of **14,** with the sole discrepancy owing to the chemical shifts of the oxymethine CH-9 (**12**: δ_H_ 3.82, δ_C_ 68.8; **14**: δ_H_ 4.86, δ_C_ 77.1). As three degrees of unsaturation were accounted for by the two double bonds and the carbonyl group, indicating **12** to be acyclic. Thus, compound **12** was the ring-opened derivative generated from the hydrolysis of the lactone moiety in compound **14**. The structure of **12** was confirmed by detailed 2D NMR analysis (Figure 2). It should be noted that the relative configuration of **14** was originally determined to be 6S*, 7S*, and 9R* in the literature [40]. However, a subsequent total synthesis of the proposed structure (6S*, 7S*, 9R*)-**14** yielded a product with NMR data that did not match those of the natural **14**. This discrepancy led the authors to suggest a revision of the structure of xylarolide [41]. In our study, the structure of natural xylarolide was confirmed to be correct by detailed NMR analysis (including the key NOESY correlations in Figure 5) and by comparison with the co-isolated analogue asperlactone A (**15**) [42], whose structure was unequivocally confirmed by X-ray crystallography. The absolute configuration of **14** was also determined to be 6*S*, 7*S*, and 9*R* by comparison of the experimental ECD spectrum with the calculated ECD curves (Figure 6). The absolute configuration of C-6, C-7, and C-9 in **12** was determined to be the same as **14** based on biogenetic considerations and sharing almost identical NMR data.

Compound **13** had the molecular formula C_12_H_22_O_5_ as determined by the negative HRESIMS ion at *m*/*z* 245.1378 ([M − H]^−^, calcd. 245.1394), two mass units over that of **12**. The NMR spectra of **13** showed similar structural features to **12**, with obvious differences being the presence of two additional methylenes (δ_C_ 34.4 and 29.0) and the absence of one double bond. The aforementioned information indicated that **13** was the hydrogenated derivative of **12**. The HMBC correlations from the extra methylene protons [δ_H_ 2.43 (2H, m) and 2.36 (2H, m)] to the sole carboxyl carbon (δ_C_ 176.2) indicated that the Δ^2^ in **12** was hydrogenated in **13**. The structure of **13** was secured by 2D NMR analysis (Figure 2). The absolute configuration of the chiral centers in **13** was proposed to be identical to that of **12** based on biogenetic considerations.

Additionally, the co-isolated known compounds were determined to be dihydrogladiolic acid methyl lactal (**5**) [43], phomotone (**6**) [38], convolvulol (**7**) [39], 4,6-dihydroxy-5-methyl-1(3H)-isobenzofuranone (**8**) [44], 2,5-dimethylresorcin (**9**) [45], xylarolide (**14)** [40], asperlactone A (**15**) [42], phomolide D (**16**) [38], 4-hydroxy-3,6-dimethyl-2(1*H*)-pyridinone (**17**) [46], and secalonic acid A (**18**) [37] by comparing the NMR data and specific rotations with those in the literature.

### 2.2. Biological Activity Assessment

The isolated compounds were evaluated for their anti-ferroptotic and anti-inflammatory activities.

#### 2.2.1. Anti-Ferroptotic Activity in an RSL3-Induced Ferroptosis Model in HT22 Cells

**Figure 7:**
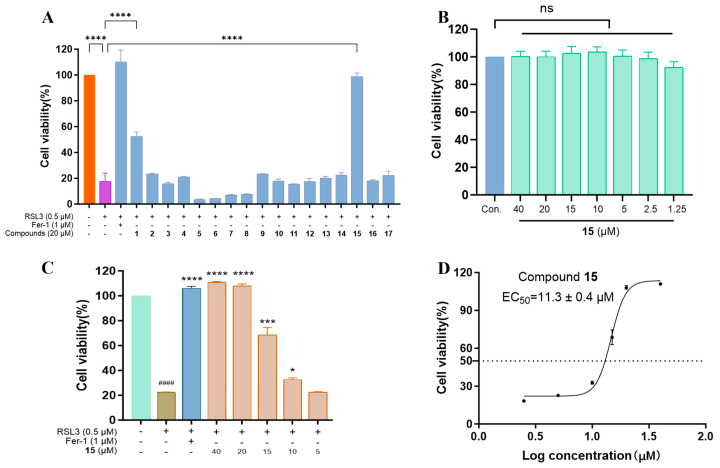
Compound 15 inhibited ferroptosis in HT22 cells. (**A**) Viability of HT22 cells treated with tested compounds (20 μM) and RSL3 (0.5 μM). (**B**) Viability of HT22 cells treated with 15 (1.25–40 μM) for 24 h in the absence of RSL3. (**C**,**D**) Dose-dependent protective effect of 15 (5–40 μM) in RSL3-induced HT22 cells. Data are presented as mean ± SD (n = 3). ^####^ *p* < 0.0001 vs. nontreated cells; * *p* < 0.05, *** *p* < 0.001, and **** *p* < 0.0001 vs. RSL3. DMSO: vehicle control; Fer-1 (1 μM): positive control.

Compounds **1**–**17** were screened for their anti-ferroptotic activity (**18** was excluded due to its known cytotoxicity) in an RSL3-induced ferroptosis model in HT22 cells. As shown in Figure 7A, treatment with 0.5 μM RSL3 reduced cell viability to roughly 20% relative to the untreated control. At a concentration of 20 μM, only compounds **1** and **15** exhibited obvious cytoprotection, restoring cell viability to 52% and 98%, respectively, whereas the remaining analogues showed negligible effects (<30% viability). Notably, **15** was non-cytotoxic to HT22 cells at concentrations up to 40 μM (Figure 7B). The potency of **15** was further assessed **via** a concentration–response study (5–40 μM), using ferrostatin-1 (Fer-1) as a positive control. As a result, compound **15** produced a dose-dependent increase in cell viability over the concentration range of 5–20 μM (Figure 7C), with an EC_50_ value of 11.3 ± 0.4 μM (Figure 7D).

**Figure 8:**
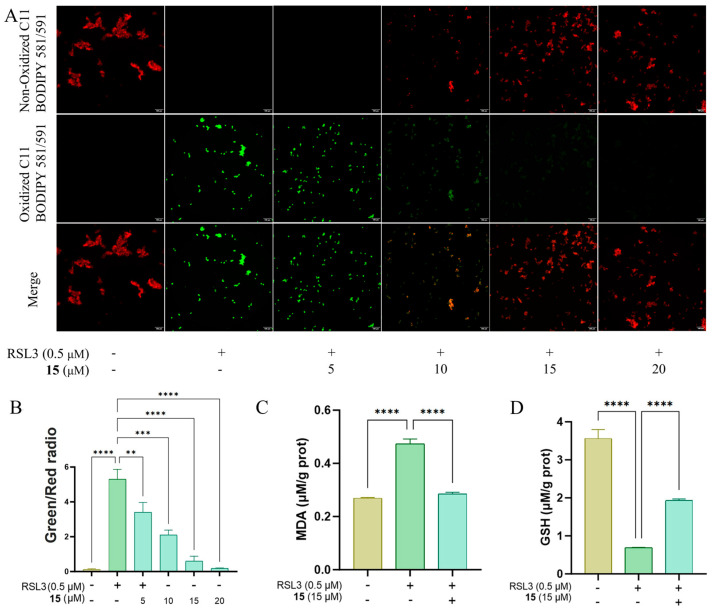
(**A**,**B**) C11 BODIPY fluorescence ratio (green/red) reflecting lipid peroxidation. (**C**) MDA levels; (**D**) GSH content; ** *p* < 0.01, *** *p* < 0.001, **** *p* < 0.0001 vs. RSL3.

To elucidate the preliminary mechanism of action underlying the anti-ferroptotic activity of compound **15**, we investigated its effect on key biomarkers of ferroptosis. Fluorescence imaging using C11-BODIPY^581/591^ fluorescent probe demonstrated that **15** concentration-dependently mitigated the pronounced lipid peroxidation induced by RSL3, with significant suppression observed at concentrations above 15 μM (Figure 8A,B). Consistent with the suppression of lipid peroxidation, compound **15** (15 μM) also attenuated the RSL3-induced elevation in MDA (Figure 8C) and reduction in GSH (Figure 8D). These results indicated that the protective effect of **15** is mediated through the inhibition of lipid peroxidation.

It is worth noting that **15** exhibited ferroptosis-inhibitory activity with a distinct scaffold that has not been previously reported among current known ferroptosis inhibitors. This study offers a new chemotype for the development of novel ferroptosis inhibitors.

#### 2.2.2. Anti-Inflammatory Activity Against LPS-Activated NO Production in RAW264.7 Macrophages

These compounds were first assessed for cytotoxicity against RAW264.7 cells at 50 μM. Compounds demonstrating >90% cell viability (compounds **6**, **7**, **13**, and **18** were excluded) were then assessed for inhibition of NO production in LPS-activated RAW264.7 macrophages, using the natural NO inhibitor quercetin as a reference (IC_50_ = 16 ± 1 μM). Only compounds **9** and **11** exhibited weak inhibitory effects, with 28.3% and 31.6% inhibition at 50 μM, respectively. The reduced activity of compound **14** compared to its epoxy derivative **11** suggested the epoxy moiety enhanced the inhibitory effect.

## 3. Experimental Section

### 3.1. General Experimental Procedure

Specific rotations were measured by an Autopol III automatic polarimeter (Rudolph Research Co., Ltd., Flanders, NJ, USA). Ultraviolet spectra were recorded on a UV-2600 spectrometer (Shimadzu Co., Kyoto, Japan). ECD spectra were obtained on an Applied Photophysics Chirascan spectrometer (Surrey, UK). The NMR spectra were acquired on a Bruker AVANCE III HD 400NMR spectrometer (Bruker, Fällanden, Switzerland) using solvent signals (Methanol-*d*_4_: δ_H_ 3.31/δ_C_ 49.0; DMSO-*d*_6_:δ_H_ 2.5/δ_C_ 39.52) as references. HRESIMS spectra were acquired on a Shimadzu LCMS-IT-TOF spectrometer (Shimadzu Co., Kyoto, Japan) equipped with an ESI source. Semi-preparative high-performance liquid chromatography (HPLC) was undertaken on a Shimadzu LC-6AD pump (Shimadzu Co., Kyoto, Japan) equipped with a UV detector, employing a YMC-Pack ODS-A HPLC (YMC Co., Ltd., Kyoto, Japan) column (250 mm × 10 mm, *S*-5 μm, 12 nm).

### 3.2. Fungal Strain and Identification

Fungus CS-HF-1 was isolated from the sediment at a depth of 0.6 m at Haikou Holiday Beach. The strain was identified as *Diaporthe searlei* through microscopic examination and internal transcribed spacer (ITS) sequencing. Its ITSsequence was deposited in GenBank under accession number PX129557, and the strain was preserved at the School of Pharmaceutical Sciences, Hainan University, China.

### 3.3. Fermentation, Extraction, and Isolation

The fermentation was carried out in 30 Fernbach flasks (500 mL), each containing 70 g of rice. Artificial seawater (90 mL) was added to each flask, and the contents were soaked for three hours before autoclaving at 15 psi for 30 min. After cooling to room temperature, each flask was inoculated with 3.0 mL of the spore inoculum and incubated at room temperature for 30 days. The fermentation was conducted in 100 Erlenmeyer flasks (500 mL), each containing 80 g of rice and 95 mL of artificial seawater. The contents were soaked for 1 h and then autoclaved for 20 min. Each flask was inoculated with 1.0 mL of the spore inoculum and incubated at room temperature (r. t.) for 30 days. The fermented material from all flasks was combined, soaked in EtOAc (5 L), and ultrasonically extracted for 1 h. After evaporation in vacuo, the EtOAc extract (14.3 g) was dissolved in methanol (50 mL) and **18** (200 mg) was collected as a precipitate. The mother liquor was further fractionated on an MCI gel column chromatography (CC) with MeOH/H_2_O (10:90 → 100:0) as eluent to yield seven fractions (F1–F7). Fraction F2 (2.7 g) was separated on an ODS silica gel CC (55 g) using a gradient elution of MeOH/H_2_O (10:90 → 60:40, 500 mL per gradient) to yield **6** subfractions (F2.1–F2.6). F2.1 (0.15 g) was purified by HPLC (YMC-Pack ODS-A column, 250 × 10 mm, *S*-5 μm, 12 nm) using MeOH/H_2_O (15:85, 2 mL/min) as the mobile phase to afford compound **17** (7.4 mg, *R*_t_ 29.0 min). Compounds **2** (2.8 mg) and **3** (3.1 mg) were obtained from fractions F2.3 (0.38 g) and F2.4 (1.37 g) by precipitation, respectively. The mother liquor from fraction F2.4 was further separated on an ODS silica gel CC using MeOH/H_2_O (10:90 → 60:40) as eluent to yield 5 subfractions (F2.4.1–F2.4.5). The subfraction F2.4.2 (419 mg) was separated by HPLC using MeOH/H_2_O (35:65) as the mobile phase to collect 7 subfractions (F2.4.2.1–F2.4.2.7). F2.4.2.2 (30 mg) was purified by HPLC using MeCN/H_2_O (17:83, 2 mL/min) as the mobile phase to afford compound **10** (2.0 mg, *R*_t_ 37.9 min). F2.4.2.3 (101 mg) was purified by HPLC using MeCN/H_2_O (16:84, 2 mL/min) as the mobile phase to afford compound **7** (2.5 mg, *R*_t_ 52.2 min) and a mixture, the latter was purified by HPLC using MeOH/H_2_O (31:69, 2 mL/min) to afford compound **8** (2.0 mg, *R*_t_ 42.0 min) and compound **15** (1.2 mg, *R*_t_ 47.2 min). F2.4.2.4 (87 mg) was purified by HPLC using MeCN/H_2_O (17:83, 2.5 mL/min) as the mobile phase to afford compound **13** (40.0 mg, *R*_t_ 30.9 min). F2.4.5 (332 mg) was separated on an ODS silica gel CC using a gradient elution of MeOH/H_2_O to yield 6 subfractions (F2.4.5.1–F2.4.5.6). F2.4.5.4 (94 mg) was purified by HPLC using MeOH/H_2_O (37:63, 2 mL/min) to afford compound **16** (8.2 mg, *R*_t_ 34.1 min). F2.4.5.5 (99 mg) was purified by HPLC using the solvent MeOH/H_2_O (37:63, 2 mL/min) to afford compounds **1** (5.3 mg, *R*_t_ 34.8 min), **4** (3.9 mg, *R*_t_ 51.2 min), **6** (6.2 mg, *R*_t_ 38.6 min), and **11** (7.2 mg, *R*_t_ 41.7 min). F2.4.5.6 (52 mg) was purified by HPLC using MeOH/H_2_O (35:65, 2 mL/min) as the mobile phase to afford compound **12** (10.3 mg, *R*_t_ 33.1 min). F2.5 (0.31 g) was purified by HPLC using MeOH/H_2_O (45:55, 2.5 mL/min) to afford compound **5** (7.1 mg, *R*_t_ 32.0 min). Fraction F4 (0.4 g) was purified on an ODS silica gel CC eluting with MeOH/H_2_O (20:80 → 60:40) to give compound **14** (128 mg). F5 (2.2 g) was separated on an ODS silica gel CC using a gradient elution of MeOH/H_2_O (10:90 → 30:70) to afford compound **9** (8.8 mg).

(±)–Diasearamide A (**1**)**:** Light yellow oil; [α]^25^_D_ 0 (*c* 0.1, MeOH); UV (MeOH) *λ*_max_ (log ε) 210 (3.73), 291 (3.02) nm; ^1^H and ^13^C NMR data, see Table 1; HRESIMS *m*/*z* 331.0909 [M + Na]^+^ (calcd. for C_14_H_16_N_2_O_6_Na^+^, 331.0901); 343.0698 [M + Cl]^−^ (calcd. for C_14_H_16_N_2_O_6_Cl^−^, 343.0702); 370.0885 [M + NO_3_]^−^ (calcd. for C_14_H_16_N_3_O_9_^−^, 370.0892).

(±)–Diasearamide B (**2**)**:** White powder; [α]^25^_D_ 0 (*c* 0.1, MeOH); UV (MeOH) *λ*_max_ (log ε) 209 (4.64), 240 (3.80), 297 (3.58) nm; ^1^H and ^13^C NMR data, see Table 1; HRESIMS *m*/*z* 495.1398 [M + Na]^+^ (calcd. for C_23_H_24_N_2_O_9_Na^+^, 495.1374); 471.1400 [M − H]^−^ (calcd. for C_23_H_23_N_2_O_9_^−^, 471.1409); 507.1162 [M + Cl]^−^ (calcd. for C_23_H_24_N_2_O_9_Cl^−^, 507.1176); 534.1338 [M + NO_3_]^−^ (calcd. for C_23_H_24_N_2_O_9_NO_3_^−^, 534.1365).

Diasearamide C (**3**)**:** White powder; UV (MeOH) *λ*_max_ (log ε) 211 (4.33), 245 (3.80) nm; HRESIMS *m*/*z* 220.0614 [M − H]^−^ (calcd. for C_11_H_10_NO_4_^−^, 220.0615).

Diasearlide acid (**4**)**:** Light yellow oil; UV (MeOH) *λ*_max_ (log ε) 218 (3.93), 263 (3.46), 294 (3.24) nm; ^1^H and ^13^C NMR data, see Table 1; HRESIMS *m*/*z* 207.0296 [M − H]^−^ (calcd. for C_10_H_7_O_5_^−^, 207.0299).

(2*S*,4*S*)-2-Hydroxy-4-propylbutyrolactone (**10**)**: *Colorless*** oil; [α]^25^_D_ −25 (*c* 0.1, MeOH); UV (MeOH) *λ*_max_ (log ε) 245, 289 nm; ECD (*c* 5.6 × 10^−4^ M, MeOH) *λ*_max_ (De) 249 (+9.48) nm. ^1^H NMR (400 MHz, methanol-*d*_4_): δ_H_ 4.53 (1H, dd, *J* = 10.7, 8.8 Hz, H-2), 2.66 (1H, m, H-3a), 1.76 (1H, m, H-3b), 4.40 (1H, m, H-4), 1.70 (1H, m, H-5a), 1.63 (1H, m, H-5b), 1.47 (2H, m, H-6), 0.98 (3H, t, *J* = 7.2 Hz, H-7); δ_C_ 179.4 (C, C-1), 69.5 (CH, C-2), 38.7 (CH_2_, C-3), 78.1 (CH, C-4), 38.6 (CH_2_, C-5), 19.4 (CH_2_, C-6), 14.1 (CH_3_, C-7); HRESIMS *m*/*z* 143.0710 [M − H]^−^ (calcd. for C_7_H_11_O_3_^−^, 143.0714).

Diasearolide A (**11**)**:** Light yellow oil; [α]^25^_D_ +33 (*c* 0.1, MeOH); UV (MeOH) *λ*_max_ (log ε) 203 (3.84) nm; ECD (*c* 2.1 × 10^−3^ M, MeOH) *λ*_max_ (Δε) 208 (+4.82), 242 (+1.96) nm. ^1^H and ^13^C NMR data, see Table 2; HRESIMS *m*/*z* 241.1065 [M − H]^−^ (calcd. for C_12_H_17_O_5_^−^, 241.1081).

Diasearolide B (**12**)**:** Light yellow oil; [α]^25^_D_ –8 (*c* 0.1, MeOH); UV (MeOH) *λ*_max_ (log ε) 254 (3.61) nm; ^1^H and ^13^C NMR data, see Table 2; HRESIMS *m*/*z* 243.1236 [M − H]^−^ (calcd. for C_12_H_19_O_5_^−^, 243.1238).

Diasearolide C (**13**)**:** Light yellow oil; [α]^25^_D_ −9 (*c* 0.1, MeOH); UV (MeOH) *λ*_max_ (log ε) 202 (3.43) nm; ^1^H and ^13^C NMR data, see Table 2; HRESIMS *m*/*z* 245.1378 [M − H]^−^ (calcd. for C_12_H_21_O_5_^−^, 245.1394).

### 3.4. ECD and NMR Calculation

ECD calculation: Conformational analysis of **10** was performed via random searching in the Sybyl-X 2.0 version using the MMFF94S force field with an energy cutoff of 3 kcal/mol. The results showed four conformers (52.88%, 28.44%, 12.83%, 5.85%) for (2*S*, 4*S*)-**10**. The conformers of **11** and **14** were determined by analysis of the NOESY correlations and referencing to the X-Ray data of **15**, followed by energy minimization. The conformers were optimized using density functional theory (DFT) at the B3LYP/6-31+g(d,p) level in MeOH using the solvation model density (SMD) by the GAUSSIAN 09 program. The energies, oscillator strengths, and rotational strengths (velocity) of the first 30 electronic excitations were calculated using the TDDFT methodology at the B3LYP/6-31+g(d,p) level using the SMD solvation model in MeOH. The ECD spectrum was simulated by the overlapping Gaussian function (half the bandwidth at 1/e peak height, σ = 0.3) [47]. To obtain the final spectra, the simulated spectra of the conformers were averaged according to the Boltzmann distribution theory and their relative Gibbs free energy (ΔG). Theoretical ECD spectra of the enantiomers were obtained by inverting the calculated ones. Comparison between calculated and experimental ECD spectra resolved the absolute configuration.

NMR calculation: The conformers (which were obtained via random searching in the Sybyl-X 2.0 version using the MMFF94S force field with an energy cutoff of 3 kcal/mol) of **1**, **2a**, and **10** were optimized using density functional theory (DFT) at the B3LYP/6-31g* level (Gaussian 09). The NMR shielding constants were calculated with the GIAO method at mPW1PW91/6-311+G(d,p) levels using the conductor polarizable calculation model (CPCM) in MeOH. The computational ^13^C NMR data were obtained by the linear regression analysis method in the literature [48].

### 3.5. Biological Study

The ferroptosis-related assays (cell culture, cell viability, MDA and GSH levels, and C11-BODIPY^581/591^ staining) were performed as described previously [31]. Additionally, the measurement of inhibitory activity against NO production followed the reported method [49].

## Figures and Tables

**Table 1:** ^1^H (400 MHz) and ^13^C NMR (101 MHz) data of **1**–**4** and ^1^H NMR data of **5**–**8** (δ in ppm, *J* in Hz).

No.	1 *^a^*		2 *^a^*		3 *^a^*		4 *^a^*		5 *^b^*	6 *^b^*	7 *^b^*	8 *^b^*
	δ_H_	δ_C_	δ_H_	δ_C_	δ_H_	δ_C_	δ_H_	δ_C_	δ_H_	δ_H_	δ_H_	δ_H_
1		166.3, C		166.6, C	4.39, br s	45.4, CH_2_		171.3, C				5.20, s
2					8.51, br s							
3	6.94, d (10.0)	81.5, CH	6.93, d (9.3)	80.9, CH		168.9, C	5.40, s	70.4, CH_2_	6.40, s	6.60, s	5.28, s	
4		144.3, C		142.2, C		143.5, C		148.8, C				
5		126.4, C		132.8, C		121.8, C		115.1, C				6.78, s
6	7.61, s	137.4, CH	7.62, s	135.5, CH	7.66, s	127.6, CH	7.75, s	130.2, CH	7.60, s	7.61, s	7.58, s	
7		132.6, C		132.4, C		132.3, C		128.1, C				
8		156.0, C		154.8, C		160.1, C		165.9, C				
9		118.3, C		117.8, C		128.1, C		108.0, C				
10	5.08, d (12.7) 4.98, d (12.7)	61.5, CH_2_	4.51, br s	58.4, CH_2_		166.4, C		170.1, C			4.68, s	
11	2.28, s	15.2, CH_3_	2.28, s	15.2, CH_3_	2.32, s	16.1, CH_3_	2.23, s	15.6, CH_3_	2.33, s	2.33, s	2.31, s	2.16, s
12		156.9, C		155.2, C								
13	3.93, s	61.7, CH_3_	3.91, s	61.6, CH_3_	3.80, s	61.3, CH_3_			4.00, s	4.00, s	4.02, s	
14		170.2, C										
15	2.06, s	20.5, CH_3_										
NH_2_	5.98, s											
3-OH	7.40, d (10.0)		7.71, d (9.3)									
3-OMe									3.58, s			
10-OH			5.30, br s									

*^a^* In DMSO-*d*_6_; *^b^* in methanol-*d*_4_.

**Table 2:** ^1^H (400 MHz) and ^13^C NMR (101 MHz) data of **11**–**14** and ^13^C NMR data of **15** and **16** in methanol-*d*_4_ (δ_H_ in ppm, *J* in Hz).

No.	11		12		13		14		15	16
	δ_H_	δ_C_	δ_H_	δ_C_	δ_H_	δ_C_	δ_H_	δ_C_	δ_C_	δ_C_
1		166.6, C		169.7, C		176.2, C		170.3, C	171.3, C	174.2, C
2	5.96, d (11.0, 1.5)	126.0, CH	5.65, d (11.4)	118.9, CH	2.43, m	34.4, CH_2_	5.91, d (10.6)	126.3, CH	40.8, CH_2_	28.4, CH_2_
3	6.52, d (11.0)	142.6, CH	6.68, dd (11.5, 11.4)	145.4, CH	2.36, m	29.0, CH_2_	6.70, d (10.6)	141.0, CH	64.9, CH	28.9, CH_2_
4	3.75, br s	57.5, CH	7.54, dd (15.4, 11.5)	128.8, CH	5.73, m	132.7, CH	6.22, d (15.3)	130.4, CH	62.1, CH	66.2, CH
5	2.60, dd (8.7, 2.0)	62.6, CH	6.13, dd (15.4, 6.4)	144.2, CH	5.54, dd (14.9, 6.0)	132.0, CH	5.36, dd (15.3, 10.1)	135.3, CH	55.3, CH	45.7, CH_2_
6	3.03, dd (8.7, 8.4)	79.3, CH	4.06, dd (6.4, 5.8)	76.7, CH	3.83, m	77.3, CH	3.78, dd (10.1, 9.1)	79.3, CH	78.8, CH	211.1, C
7	3.58, dd (8.4, 8.4)	73.7, CH	3.80, m	72.3, CH	3.70, m	72.5, CH	3.38, dd (9.1, 8.6)	78.0, CH	74.1, CH	75.3, CH
8	2.03, m	41.6, CH_2_	1.51, m	41.2, CH_2_	1.47, m	41.2, CH_2_	1.88, dd (17.0, 8.6) 1.79 (17.0)	42.0, CH_2_	41.6, CH_2_	39.6, CH_2_
9	4.88, m	76.3, CH	3.82, m	68.8, CH	3.81, m	68.9, CH	4.86, m	77.1, CH	74.6, CH	73.8, CH
10	1.69, m; 1.61, m	38.5, CH_2_	1.43, m	41.5, CH_2_	1.44, m	41.4, CH_2_	1.54, m	40.0, CH_2_	39.0, CH_2_	37.1, CH_2_
11	1.39, m	19.3, CH_2_	1.44, m; 1.37, m	19.9, CH_2_	1.44, m; 1.38, m	19.9, CH_2_	1.34, m	19.5, CH_2_	19.2, CH_2_	20.0, CH_2_
12	0.95, t (7.2)	14.3, CH_3_	0.93, t (6.9)	14.4, CH_3_	0.93, t (6.6)	14.4, CH_3_	0.92, t (7.4)	14.2, CH_3_	14.3, CH_3_	14.1, CH_3_

## Data Availability

Data is contained within the article and Supplementary Materials.

## Supplementary Materials

The following supporting information can be downloaded at: https://www.mdpi.com/article/10.3390/md23100402/s1, Figure S1: ^1^H NMR spectrum of the EtOAc extract of *Diaporthe searlei* CS-HF-1; Figures S2−S25, Figures S34−S57, and Figures S68−S75: 1D and 2D NMR, and HRESIMS spectra of **1**–**4** and **10**–**13**; Figures S26−S33 and Figures S58−S67: ^1^H and ^13^CNMR spectra of **5** –**9** and **14**–**18**; Tables S1–S3: Calculated NMR data of **1**, **2a**, and **10**.
