# Supplementary material for: New Polyketides and a Ferroptosis Inhibitor from the Marine-Derived Fungus Diaporthe searlei CS-HF-1
Source: Mar Drugs. 2025 Oct 16;23(10):402. doi: 10.3390/md23100402 (PMC12565626; doi:10.3390/md23100402)
Supplement: Supplementary file 1 [file marinedrugs-23-00402-s001.zip › marinedrugs-3855737-supplementary111111.pdf]

New Polyketides and a Ferroptosis Inhibitor from the Marine-Derived Fungus *Diaporthe searlei* CS-HF-1

Jicheng Xiao [1,†], Peng Wu [1,†], Yan Zhang [1,†], Qi Lv [1], Yulang Chi [3], Wei Xu [4,5], Wenzhen Lin [2,*] and Zhongbin Cheng[1,*]

[1] Key Laboratory of Tropical Biological Resources of Ministry of Education, School of Pharmaceutical Sciences, Hainan University, Haikou 570228, China; extrayx@126.com (J.X.); pengwu0820@163.com (P.W.); 17373797879@163.com (Y.Z.); 15974358897@163.com (Q.L.)

[2] Fujian Institute of Subtropical Botany, Xiamen 361006, China

[3] College of Oceanology and Food Science, Quanzhou Normal University, Quanzhou 362000, China; ylchi@qztc.edu.cn

[4] School of Basic Medical Sciences, YiChun University, Yichun 336000, China; xwkhj@163.com

[5] Xiamen Chenge Biotechnology Co., Ltd., Xiamen 361000, China

* Correspondence: chengzhongbin@hainanu.edu.cn (Z.C.); linwenzhen0703@163.com (W.L.)

† These authors contributed equally to this work.

No.	Content	Page
**Figure S1:**	^{1}H NMR spectrum of the EtOAc extract of *Diaporthe searlei* CS-HF-1 in methanol-d_4 (400 MHz)	3
**Figure S2:**	^{1}H NMR spectrum of **1** in DMSO-d_6 (400 MHz)	4
**Figure S3:**	^{13}C NMR spectrum of **1** in DMSO-d_6 (101 MHz)	4
**Figure S4:**	HSQC spectrum of **1** in DMSO-d_6	5
**Figure S5:**	HMBC spectrum of **1** in DMSO-d_6	5
**Figure S6:**	COSY spectrum of **1** in DMSO-d_6	6
**Figure S7:**	NOESY spectrum of **1** in DMSO-d_6	6
**Figure S8:**	^{1}H NMR spectrum of **2** in DMSO-d_6 (400 MHz)	7
**Figure S9:**	^{13}C NMR spectrum of **2** in DMSO-d_6 (101 MHz)	7
**Figure S10:**	HSQC spectrum of **2** in DMSO-d_6	8
**Figure S11:**	HMBC spectrum of **2** in DMSO-d_6	8
**Figure S12:**	COSY spectrum of **2** in DMSO-d_6	9
**Figure S13:**	NOESY spectrum of **2** in DMSO-d_6	9
**Figure S14:**	^{1}H NMR spectrum of **3** in DMSO-d_6 (400 MHz)	10
**Figure S15:**	^{13}C NMR spectrum of **3** in DMSO-d_6 (101 MHz)	10
**Figure S16:**	HSQC spectrum of **3** in DMSO-d_6	11
**Figure S17:**	HMBC spectrum of **3** in DMSO-d_6	11
**Figure S18:**	COSY spectrum of **3** in DMSO-d_6	12
**Figure S19:**	NOESY spectrum of **3** in DMSO-d_6	12
**Figure S20:**	^{1}H NMR spectrum of **4** in methanol-d_4 (400 MHz)	13
**Figure S21:**	^{13}C NMR spectrum of **4** in methanol-d_4 (101 MHz)	13
**Figure S22:**	HSQC spectrum of **4** in methanol-d_4	14
**Figure S23:**	HMBC spectrum of **4** in methanol-d_4	14
**Figure S24:**	COSY spectrum of **4** in methanol-d_4	15
**Figure S25:**	NOESY spectrum of **4** in methanol-d_4	15
**Figure S26:**	^{1}H NMR spectrum of **5** in DMSO-d_6 (400 MHz)	16
**Figure S27:**	^{13}C NMR spectrum of **5** in DMSO-d_6 (101 MHz)	16
**Figure S28:**	^{1}H NMR spectrum of **6** in methanol-d_4 (400 MHz)	17
**Figure S29:**	^{1}H NMR spectrum of **7** in methanol-d_4 (400 MHz)	17
**Figure S30:**	^{1}H NMR spectrum of **8** in methanol-d_4 (400 MHz)	18
**Figure S31:**	^{13}C NMR spectrum of **8** in methanol-d_4 (101 MHz)	18
**Figure S32:**	^{1}H NMR spectrum of **9** in methanol-d_4 (400 MHz)	19
**Figure S33:**	^{13}C NMR spectrum of **9** in methanol-d_4 (101 MHz)	19
**Figure S34:**	^{1}H NMR spectrum of **10** in methanol-d_4 (400 MHz)	20

**Figure S35:**	^{13}C NMR spectrum of **10** in methanol-d_4 (101 MHz)	20
**Figure S36:**	HSQC spectrum of **10** in methanol-d_4	21
**Figure S37:**	HMBC spectrum of **10** in methanol-d_4	21
**Figure S38:**	COSY spectrum of **10** in methanol-d_4	22
**Figure S39:**	NOESY spectrum of **10** in methanol-d_4	22
**Figure S40:**	^{1}H NMR spectrum of **11** in methanol-d_4 (400 MHz)	23
**Figure S41:**	^{13}C NMR spectrum of **11** in methanol-d_4 (101 MHz)	23
**Figure S42:**	HSQC spectrum of **11** in methanol-d_4	24
**Figure S43:**	HMBC spectrum of **11** in methanol-d_4	24
**Figure S44:**	COSY spectrum of **11** in methanol-d_4	25
**Figure S45:**	NOESY spectrum of **11** in methanol-d_4	25
**Figure S46:**	^{1}H NMR spectrum of **12** in methanol-d_4 (400 MHz)	26
**Figure S47:**	^{13}C NMR spectrum of **12** in methanol-d_4 (101 MHz)	26
**Figure S48:**	HSQC spectrum of **12** in methanol-d_4	27
**Figure S49:**	HMBC spectrum of **12** in methanol-d_4	27
**Figure S50:**	COSY spectrum of **12** in methanol-d_4	28
**Figure S51:**	NOESY spectrum of **12** in methanol-d_4	28
**Figure S52:**	^{1}H NMR spectrum of **13** in methanol-d_4 (400 MHz)	29
**Figure S53:**	^{13}C NMR spectrum of **13** in methanol-d_4 (101 MHz)	29
**Figure S54:**	HSQC spectrum of **13** in methanol-d_4	30
**Figure S55:**	HMBC spectrum of **13** in methanol-d_4	30
**Figure S56:**	COSY spectrum of **13** in methanol-d_4	31
**Figure S57:**	NOESY spectrum of **13** in methanol-d_4	31
**Figure S58:**	^{1}H NMR spectrum of **14** in methanol-d_4 (400 MHz)	32
**Figure S59:**	^{13}C NMR spectrum of **14** in methanol-d_4 (101 MHz)	32
**Figure S60:**	^{1}H NMR spectrum of **15** in methanol-d_4 (400 MHz)	33
**Figure S61:**	^{13}C NMR spectrum of **15** in methanol-d_4 (101 MHz)	33
**Figure S62:**	^{1}H NMR spectrum of **16** in methanol-d_4 (400 MHz)	34
**Figure S63:**	^{13}C NMR spectrum of **16** in methanol-d_4 (101 MHz)	34
**Figure S64:**	^{1}H NMR spectrum of **17** in DMSO-d_6 (400 MHz)	35
**Figure S65:**	^{13}C NMR spectrum of **17** in DMSO-d_6 (101 MHz)	35
**Figure S66:**	^{1}H NMR spectrum of **18** in DMSO-d_6 (400 MHz)	36
**Figure S67:**	^{13}C NMR spectrum of **18** in DMSO-d_6 (101 MHz)	36
**Figure S68:**	HRESIMS spectrum of compound **1**	37
**Figure S69:**	HRESIMS spectrum of compound **2**	37
**Figure S70:**	HRESIMS spectrum of compound **3**	38
**Figure S71:**	HRESIMS spectrum of compound **4**	38
**Figure S72:**	HRESIMS spectrum of compound **10**	38
**Figure S73:**	HRESIMS spectrum of compound **11**	38
**Figure S74:**	HRESIMS spectrum of compound **12**	39
**Figure S75:**	HRESIMS spectrum of compound **13**	39
**Table S1:**	Shielding sensors (σ_C) of the conformers (**1**) and the calculated chemical shifts (δ_C).	39
**Table S2:**	Shielding sensors (σ_C) of the conformer (**2**) and the calculated chemical shifts (δ_C).	40
**Table S3:**	Shielding sensors (σ_C) of the conformer (**10**) and the calculated chemical shifts (δ_C).	40

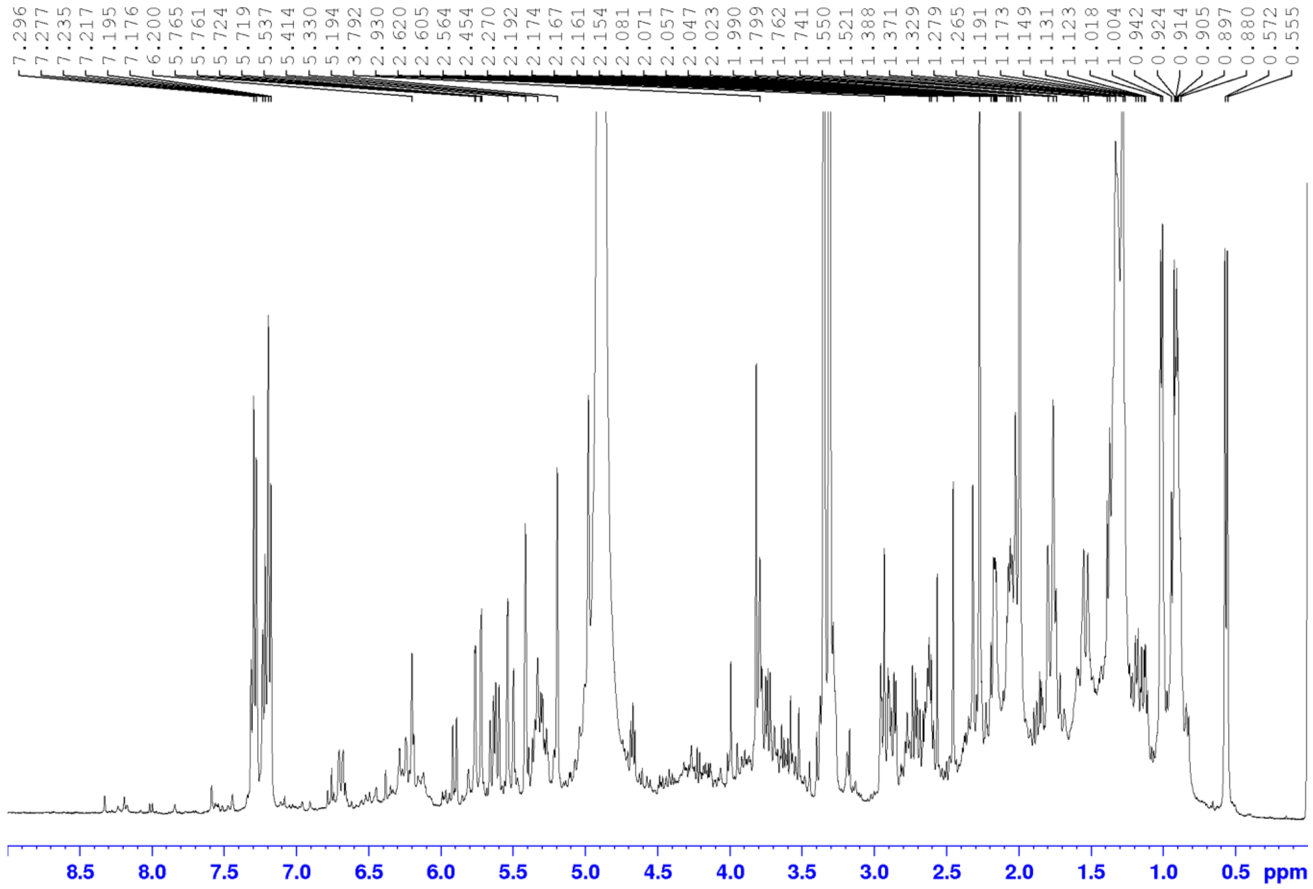

Figure S1: ^{1}H NMR spectrum of the EtOAc extract of *Diaporthe searlei* CS-HF-1 in methanol-d_4 (400 MHz)

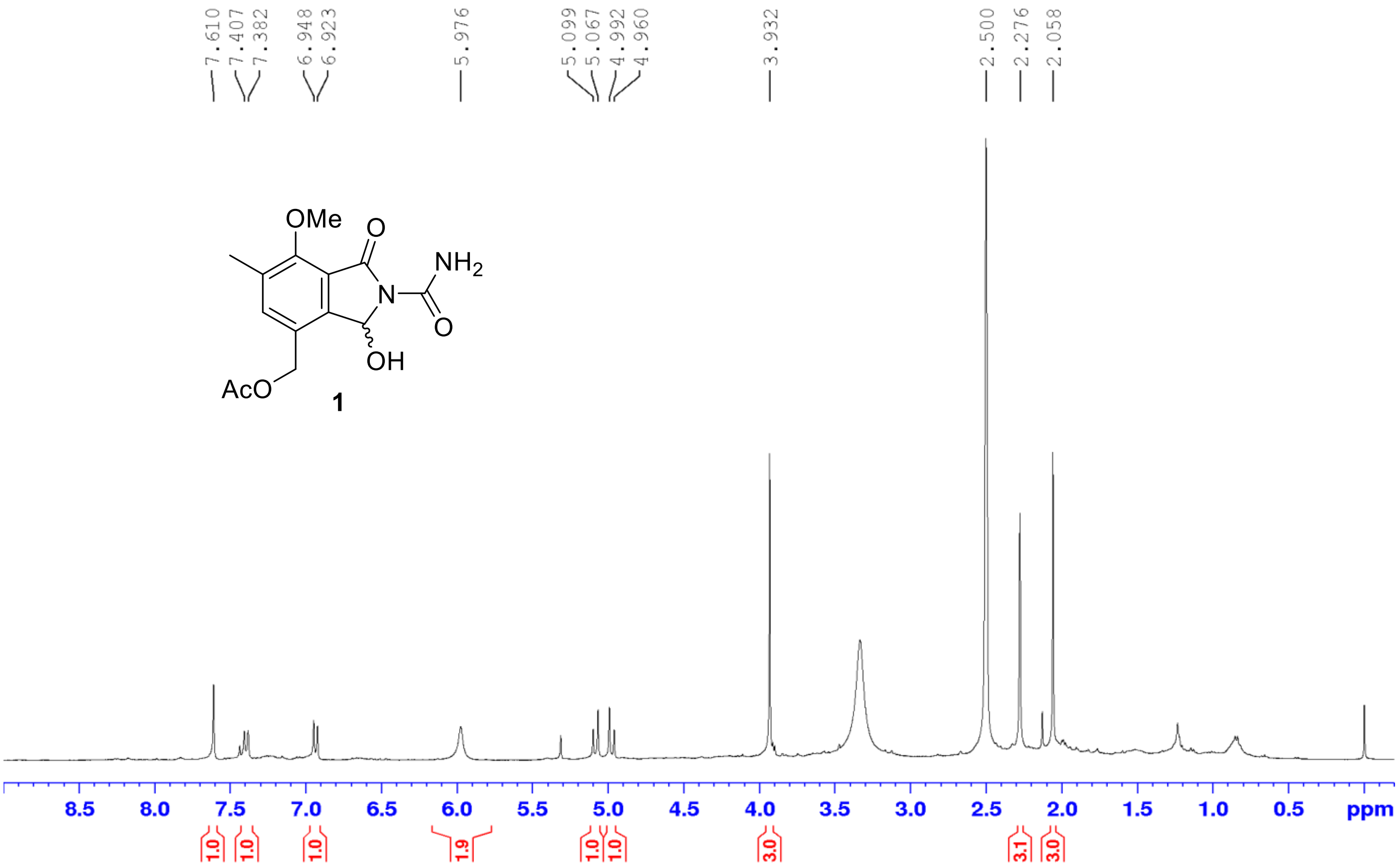

Figure S2: ^{1}H NMR spectrum of **1** in DMSO-d_6 (400 MHz)

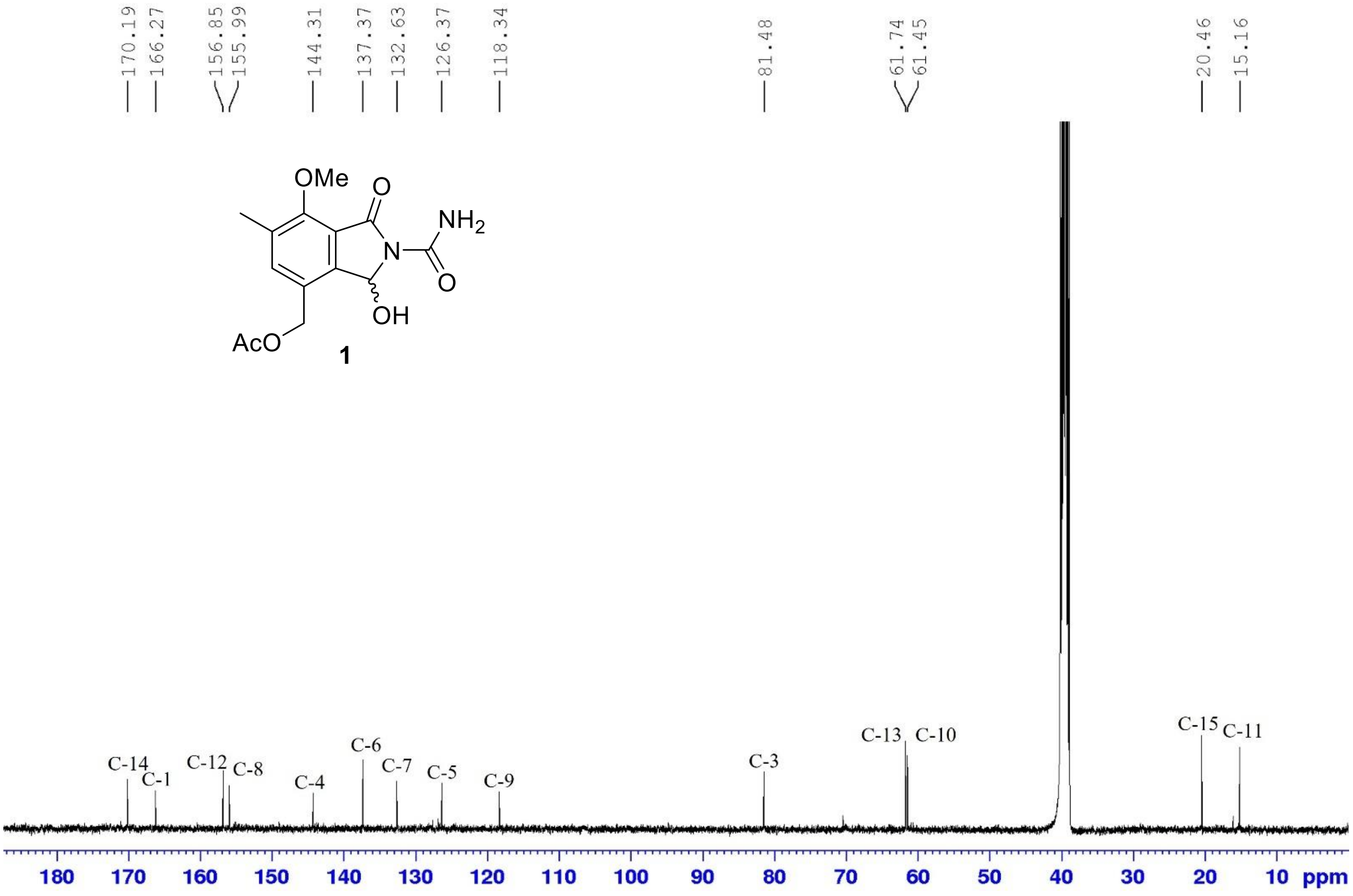

Figure S3: ^{13}C NMR spectrum of **1** in DMSO-d_6 (101 MHz)

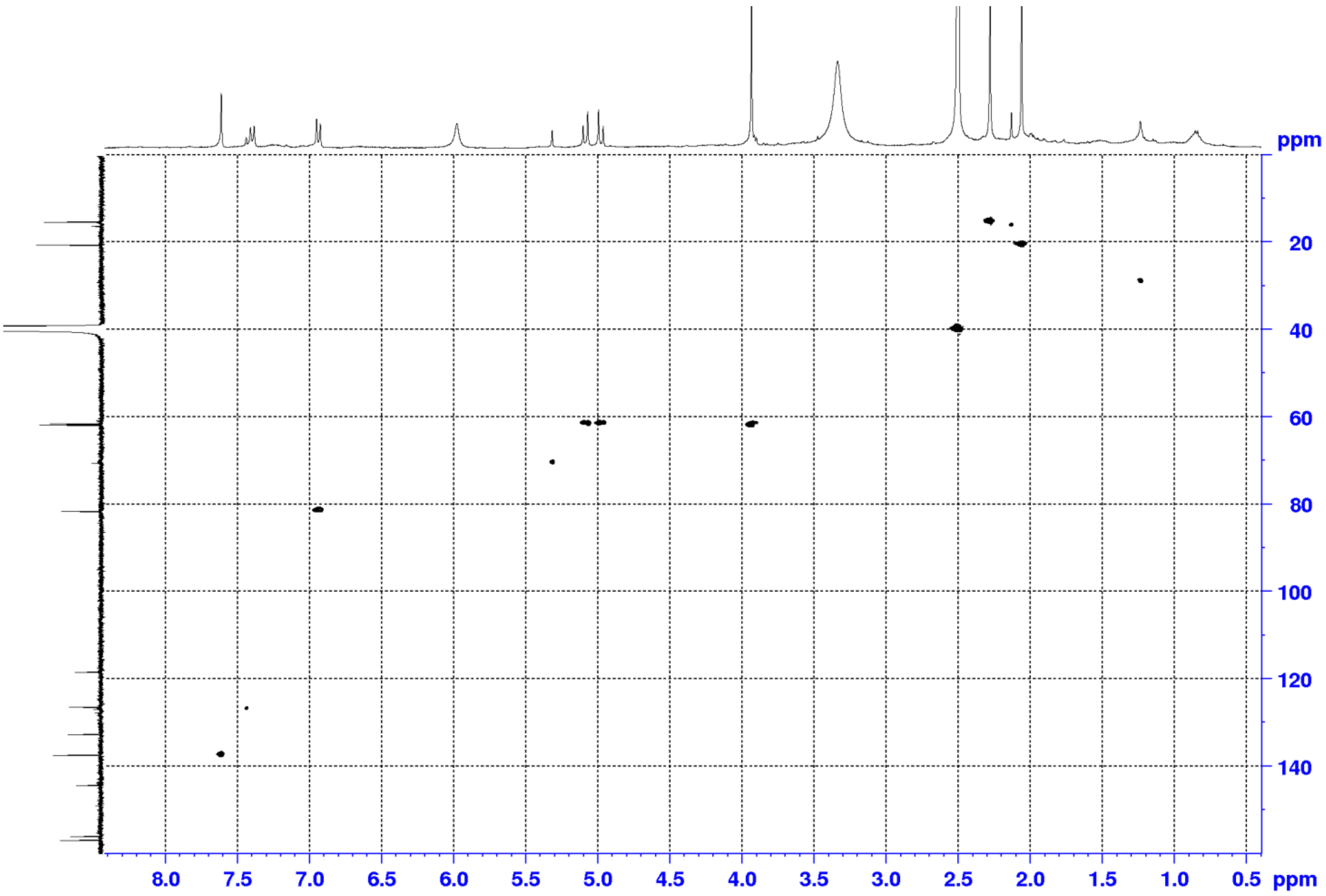

Figure S4: HSQC spectrum of **1** in DMSO-d_6

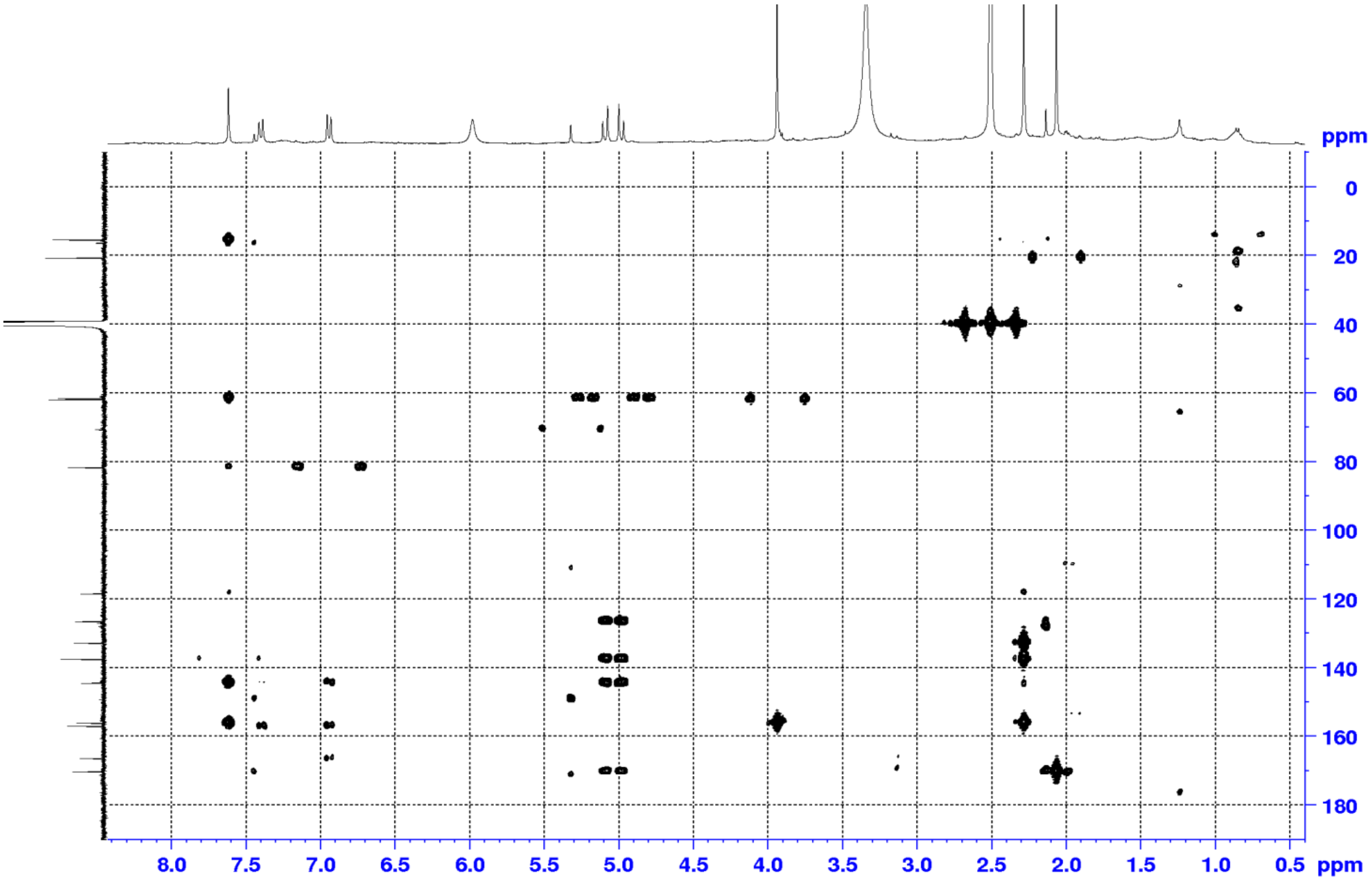

Figure S5: HMBC spectrum of **1** in DMSO-d_6

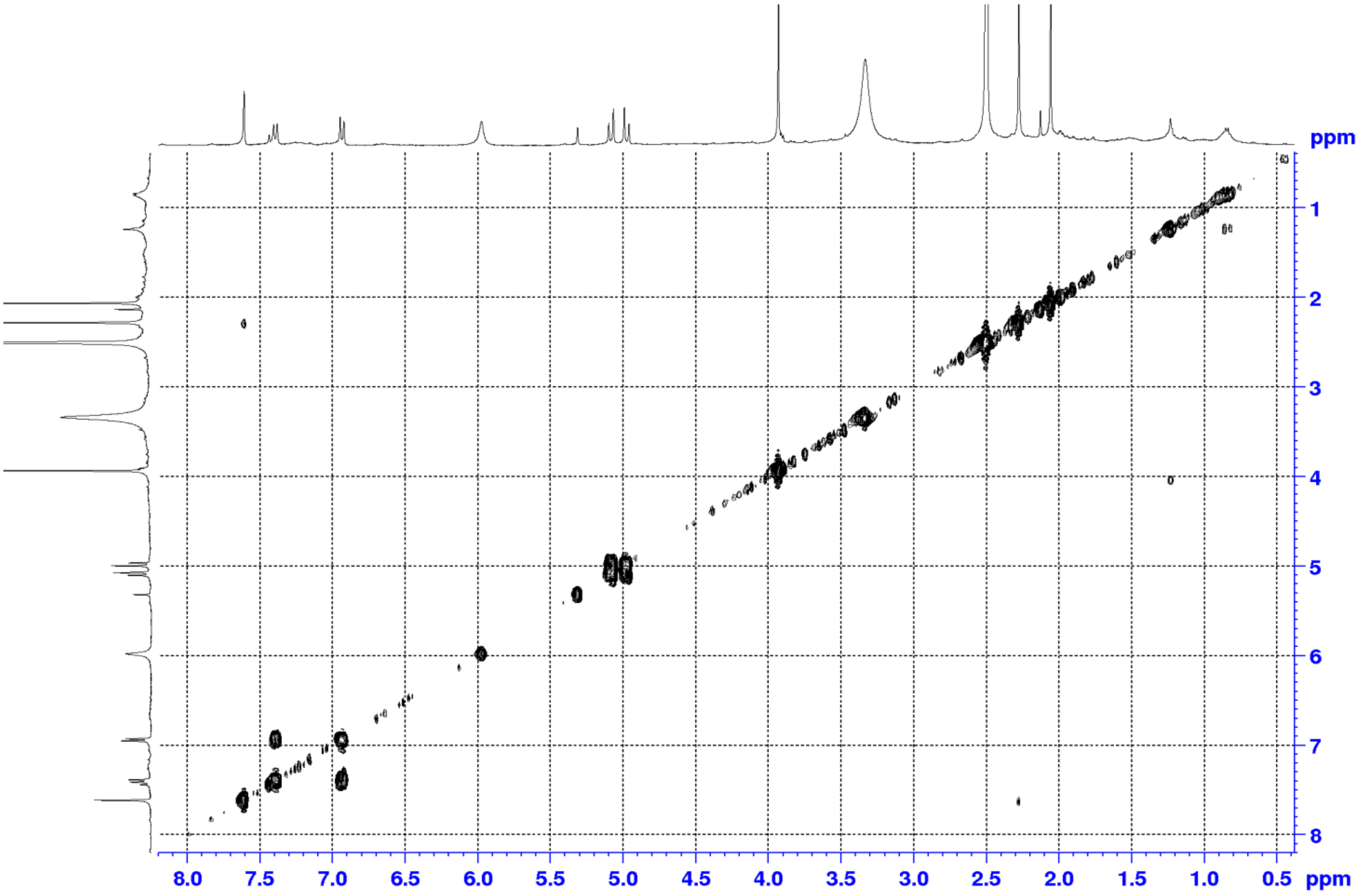

Figure S6: COSY spectrum of **1** in DMSO-d_6

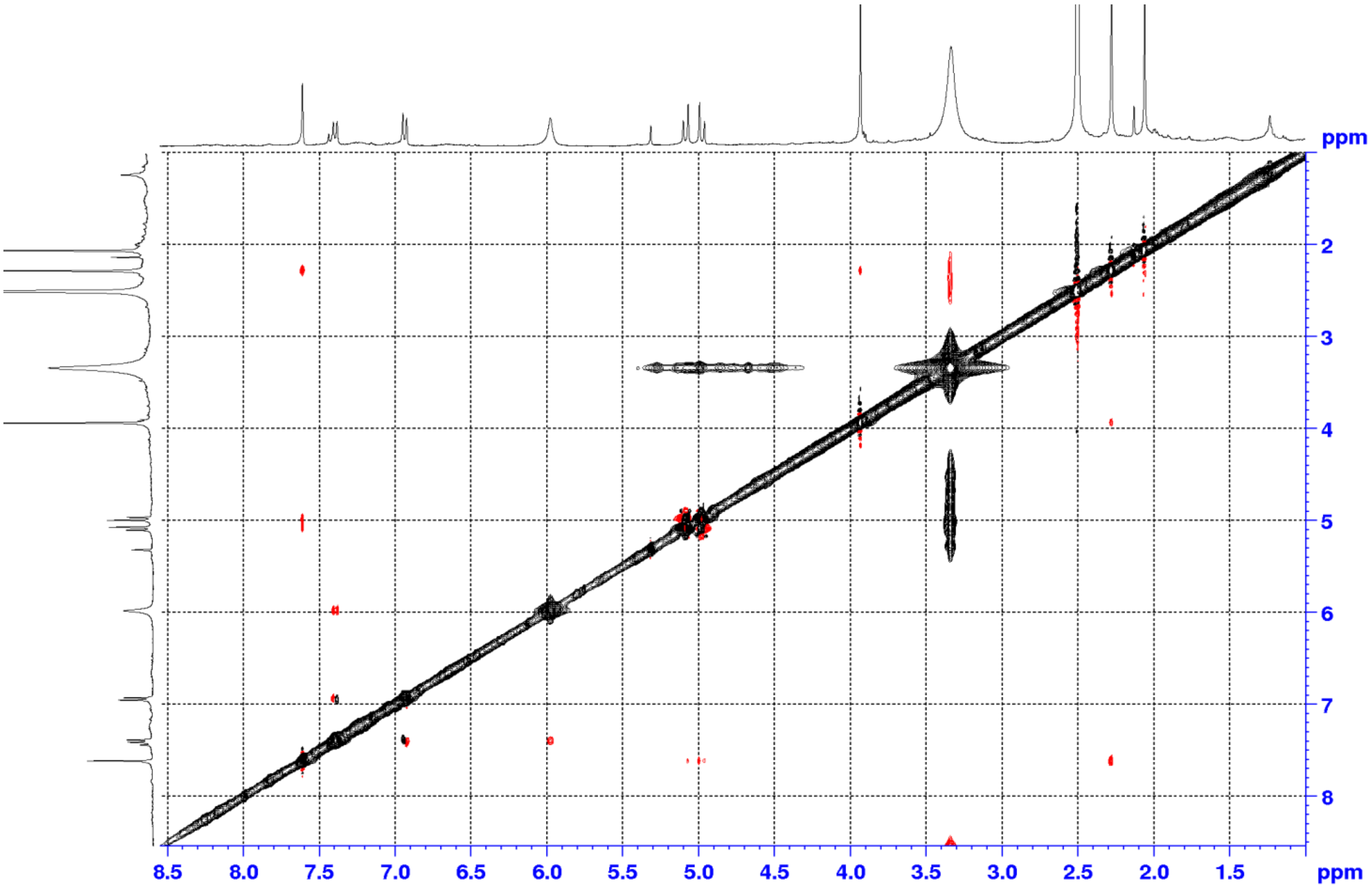

Figure S7: NOESY spectrum of **1** in DMSO-d_6

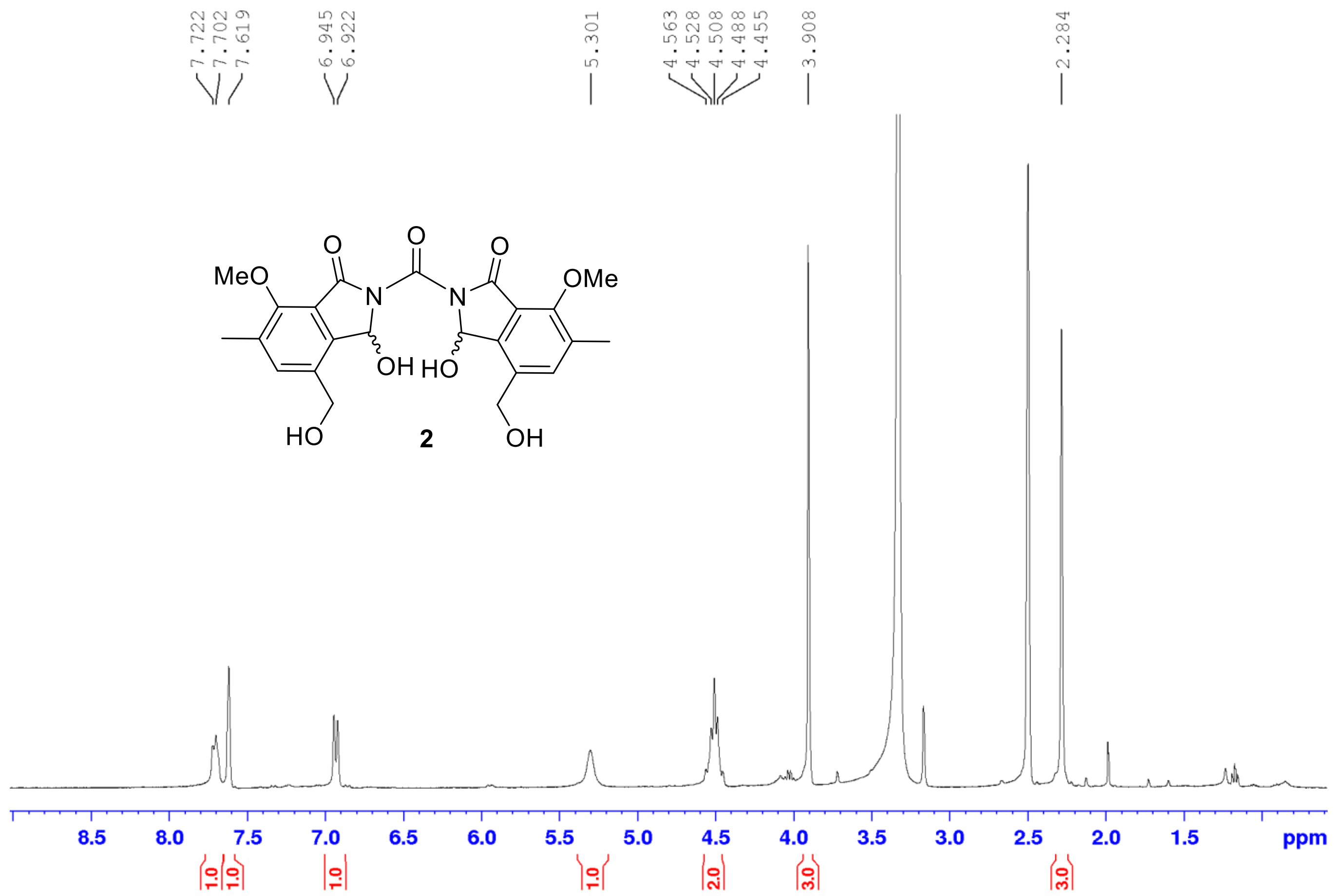

Figure S8: ^{1}H NMR spectrum of **2** in DMSO-d_6 (400 MHz)

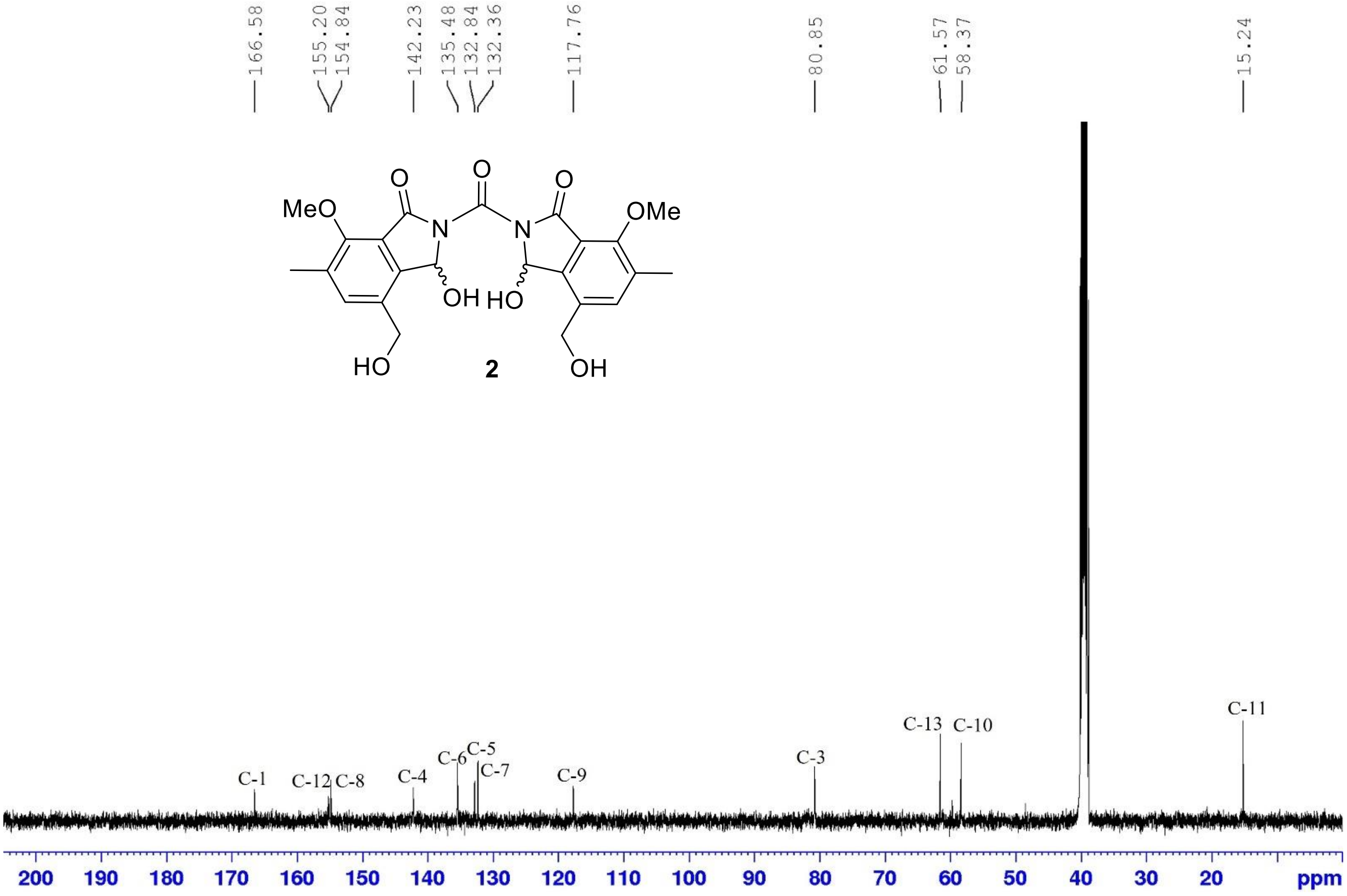

Figure S9: ^{13}C NMR spectrum of **2** in DMSO-d_6 (101 MHz)

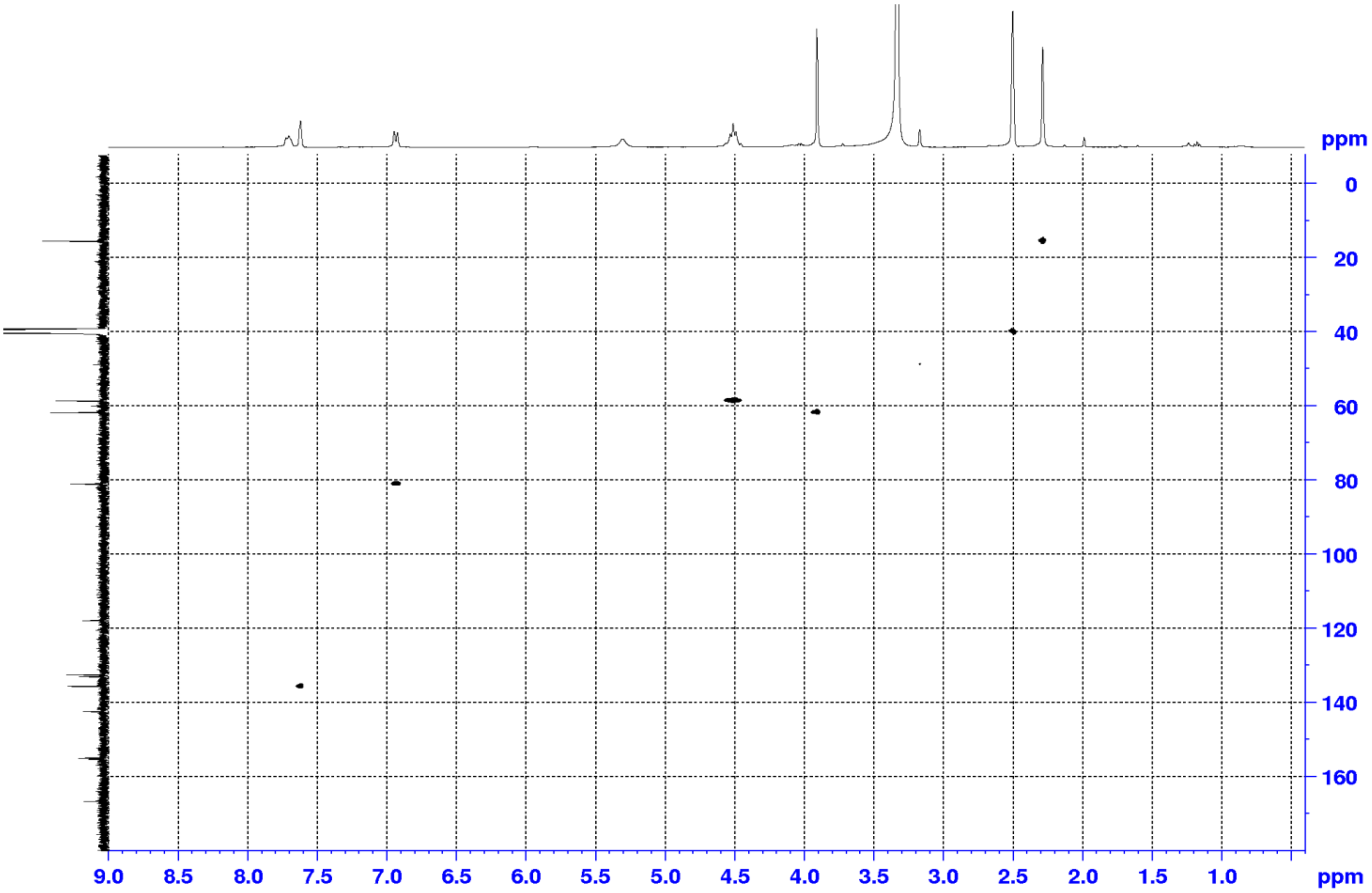

Figure S10: HSQC spectrum of **2** in DMSO-d_6

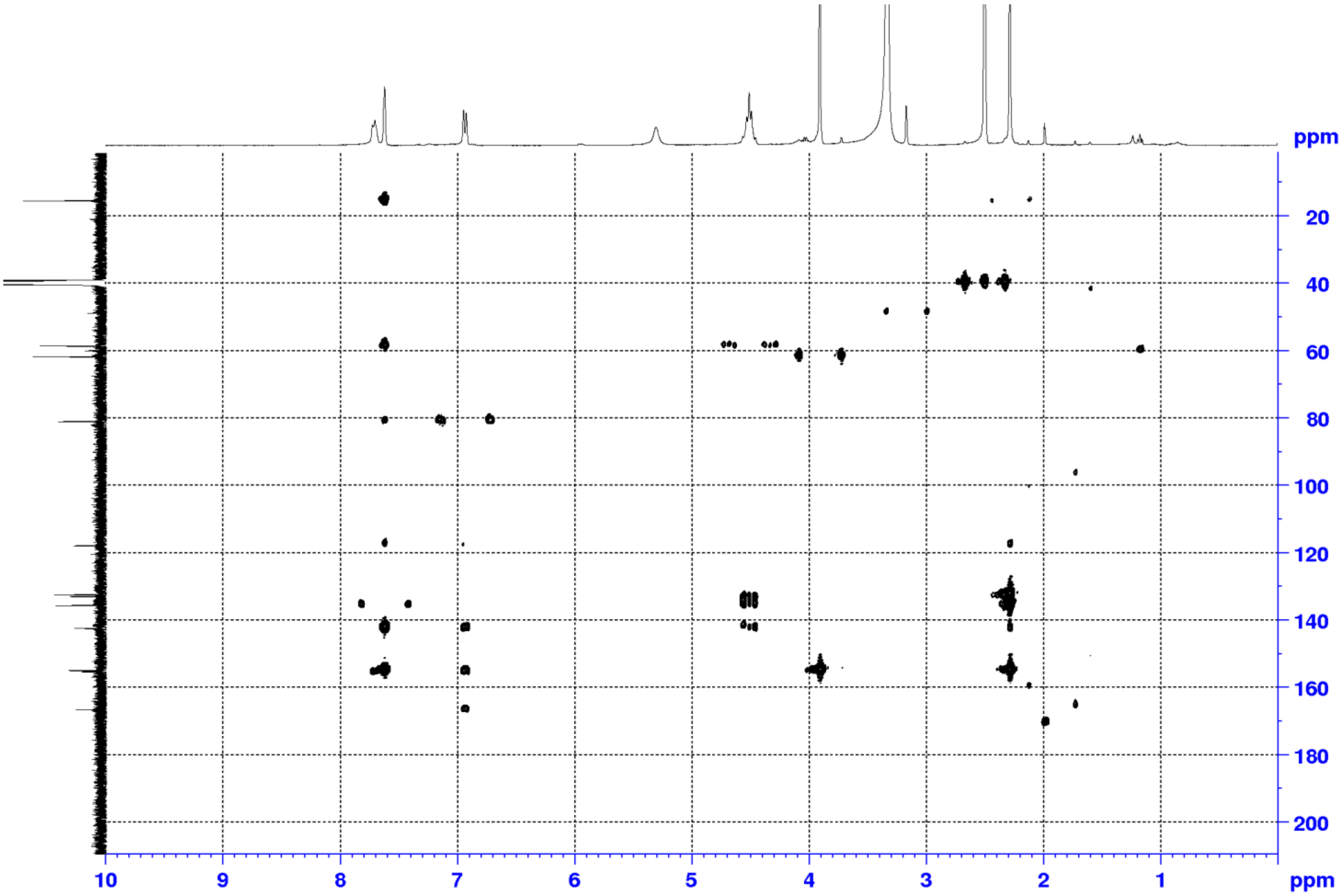

Figure S11: HMBC spectrum of **2** in DMSO-d_6

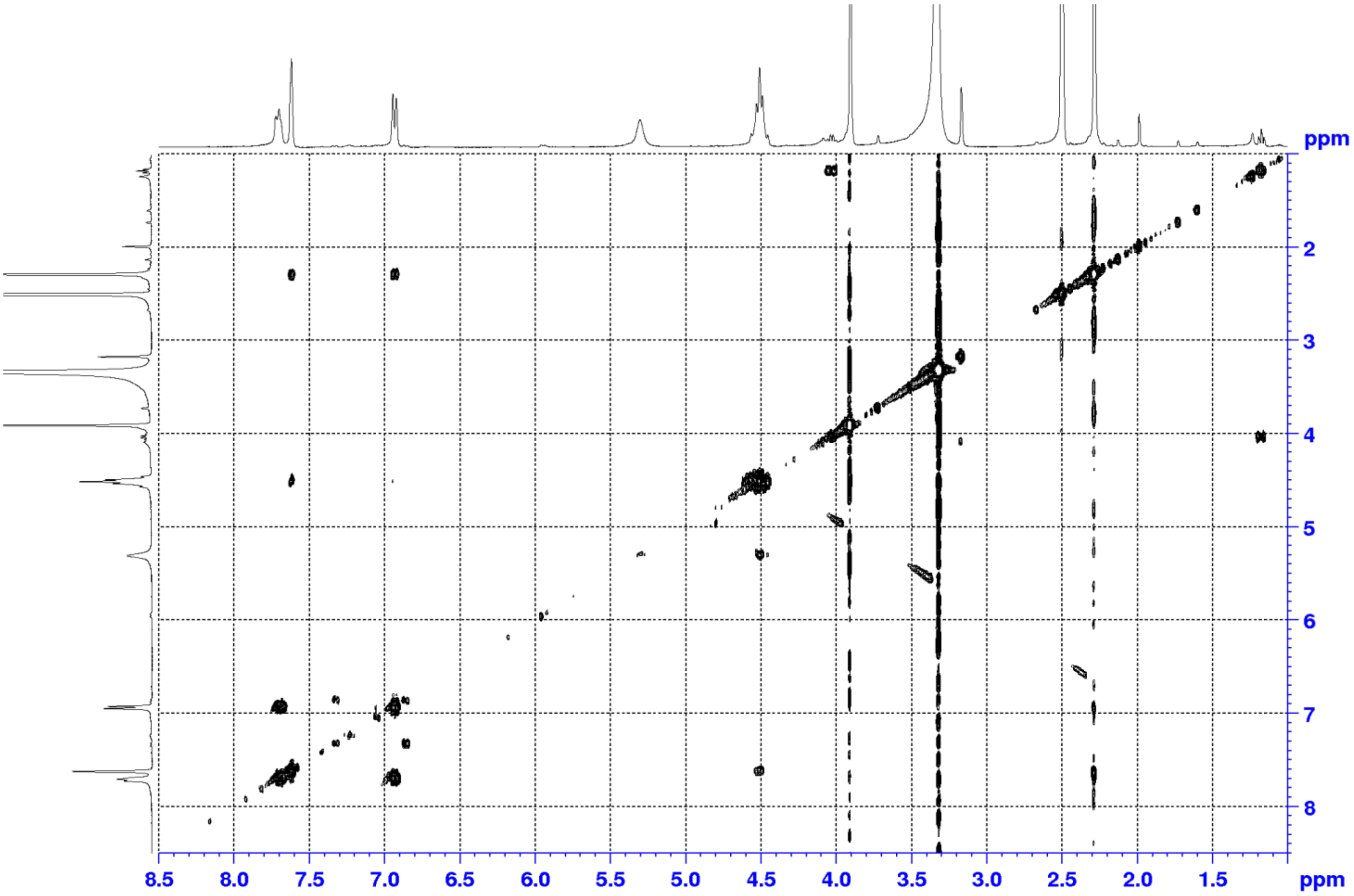

Figure S12: COSY spectrum of **2** in DMSO-d_6

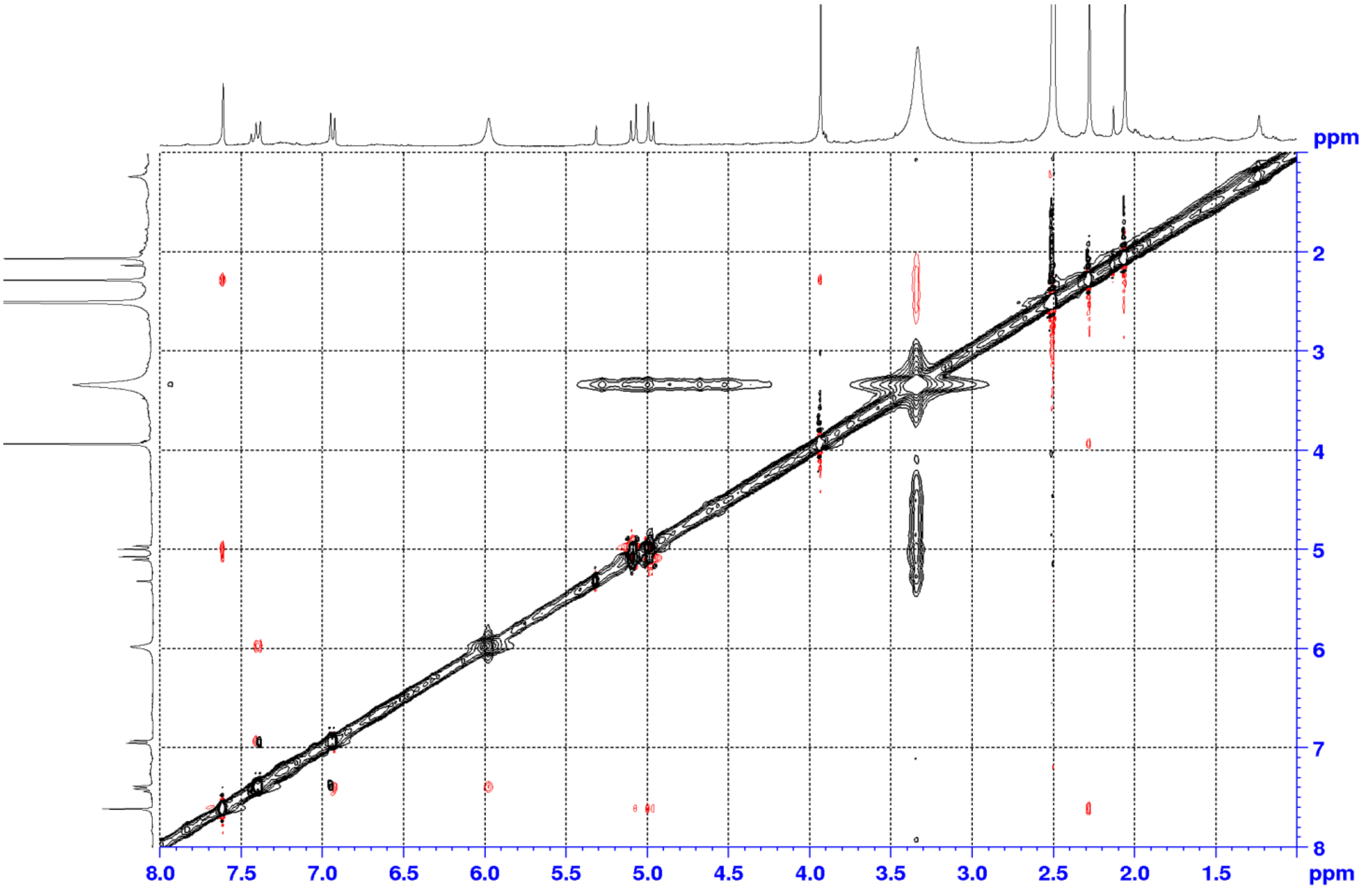

Figure S13: NOESY spectrum of **2** in DMSO-d_6

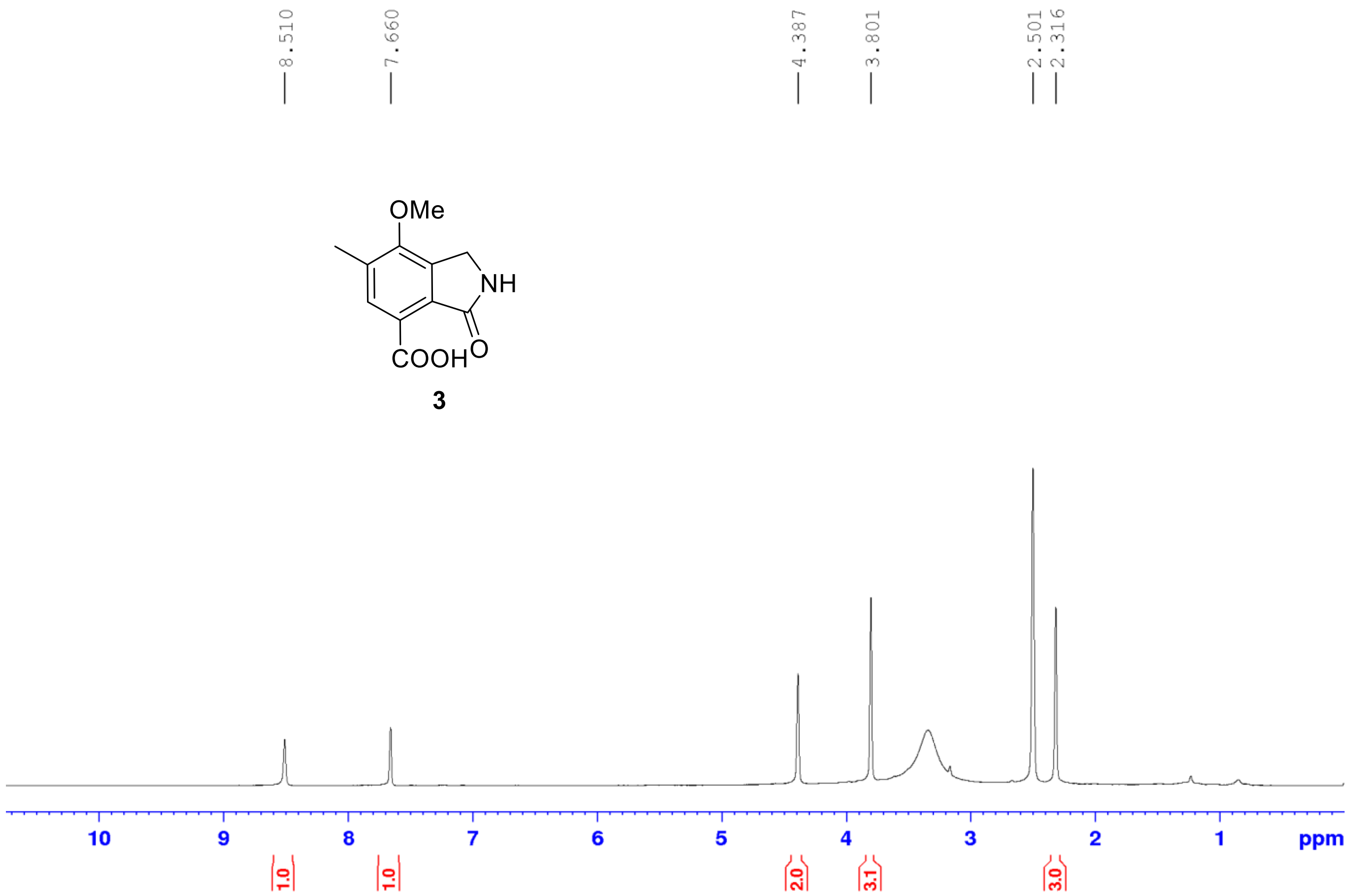

Figure S14: ^{1}H NMR spectrum of **3** in DMSO-d_6 (400 MHz)

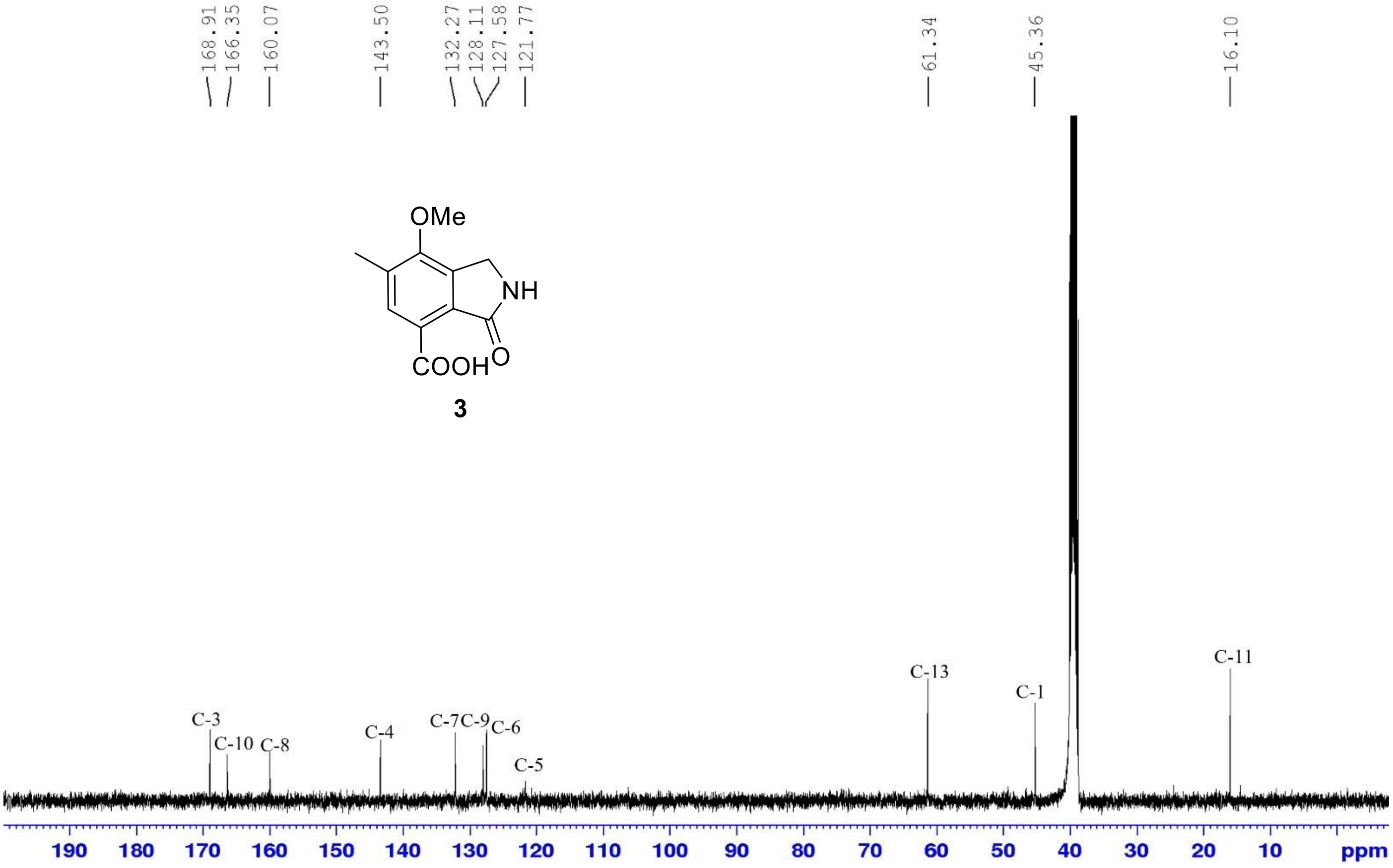

Figure S15: ^{13}C NMR spectrum of **3** in DMSO-d_6 (101 MHz)

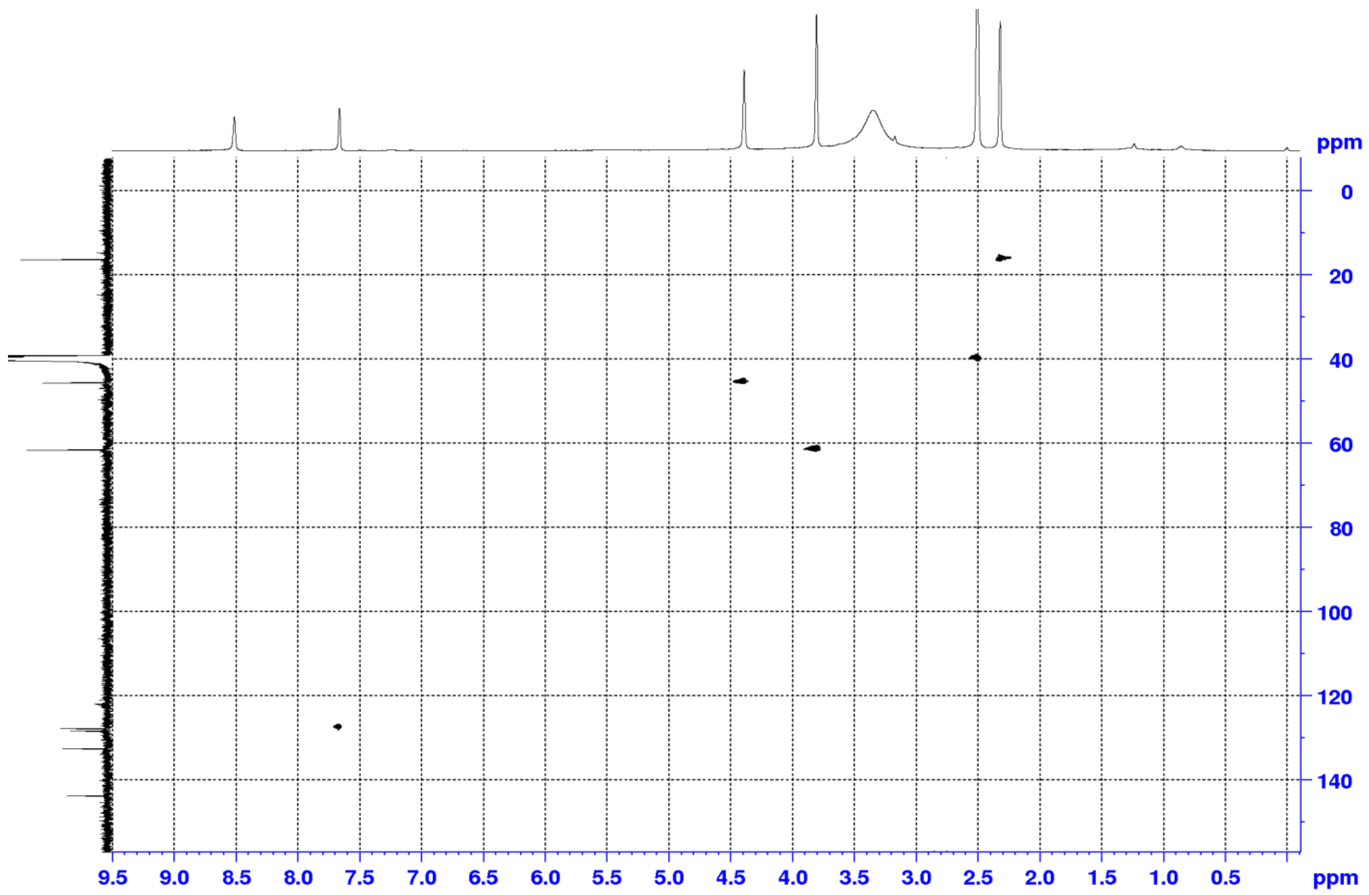

Figure S16: HSQC spectrum of **3** in DMSO-d_6

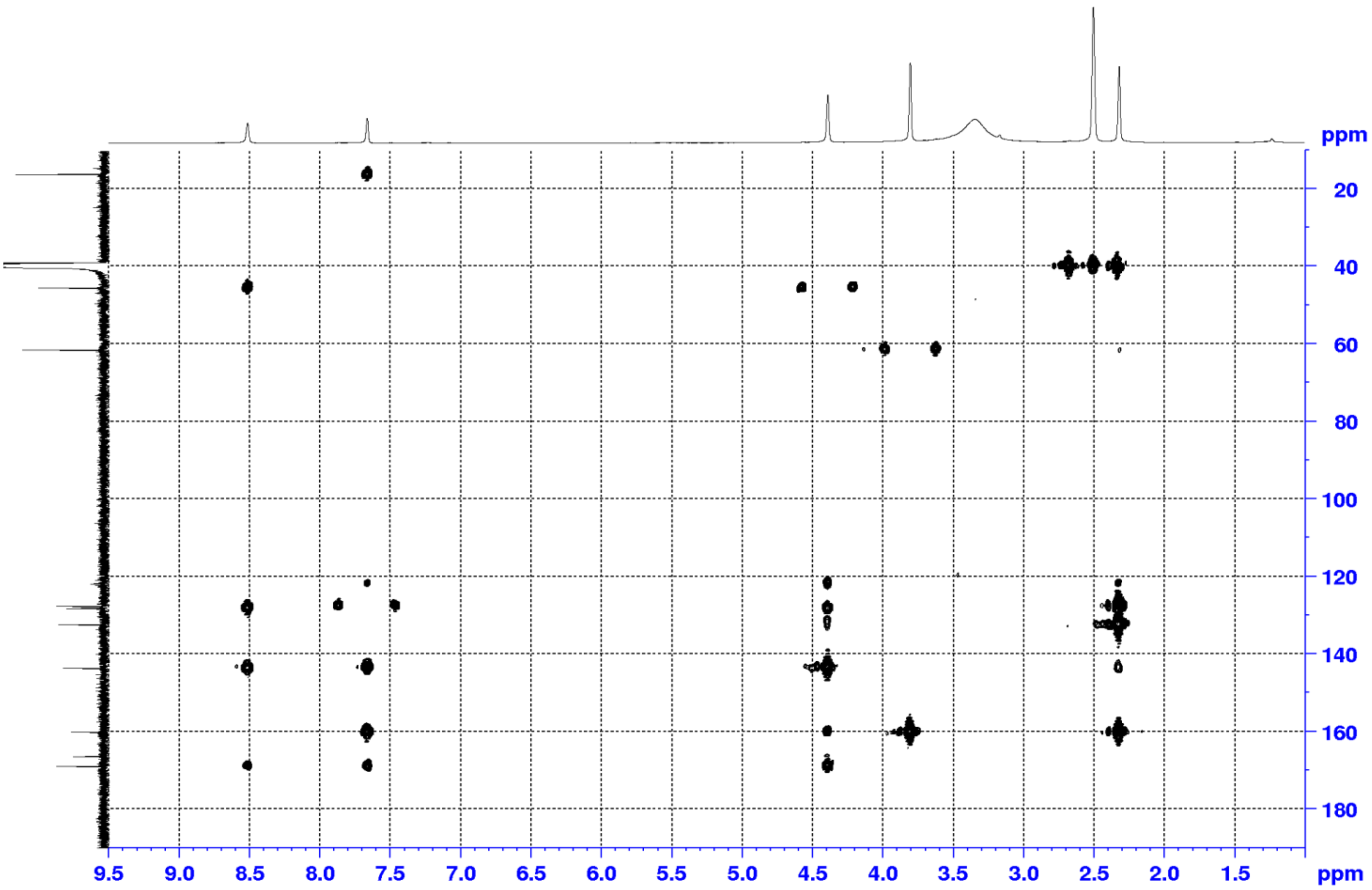

Figure S17: HMBC spectrum of **3** in DMSO-d_6

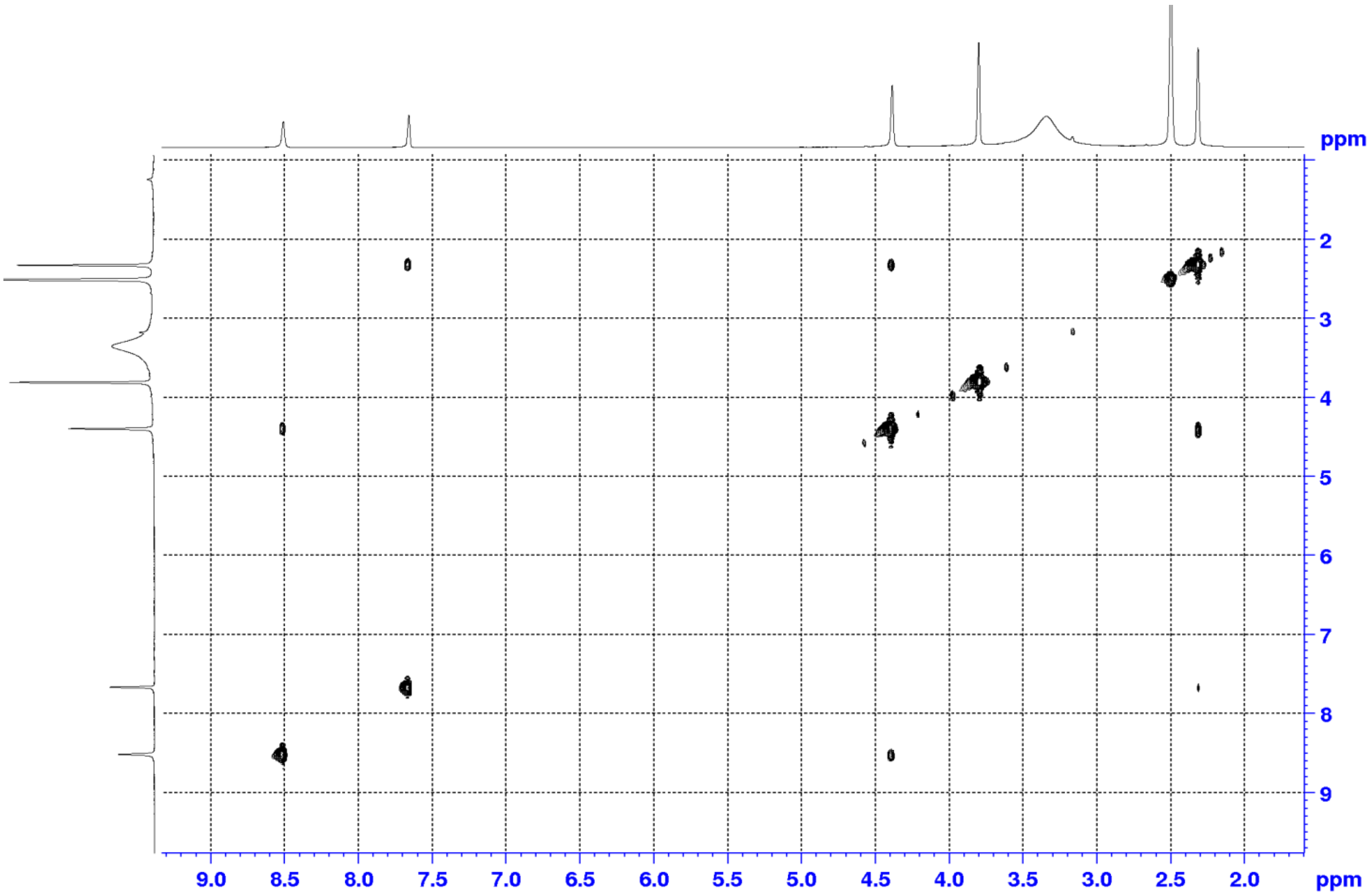

Figure S18: COSY spectrum of **3** in DMSO-d_6

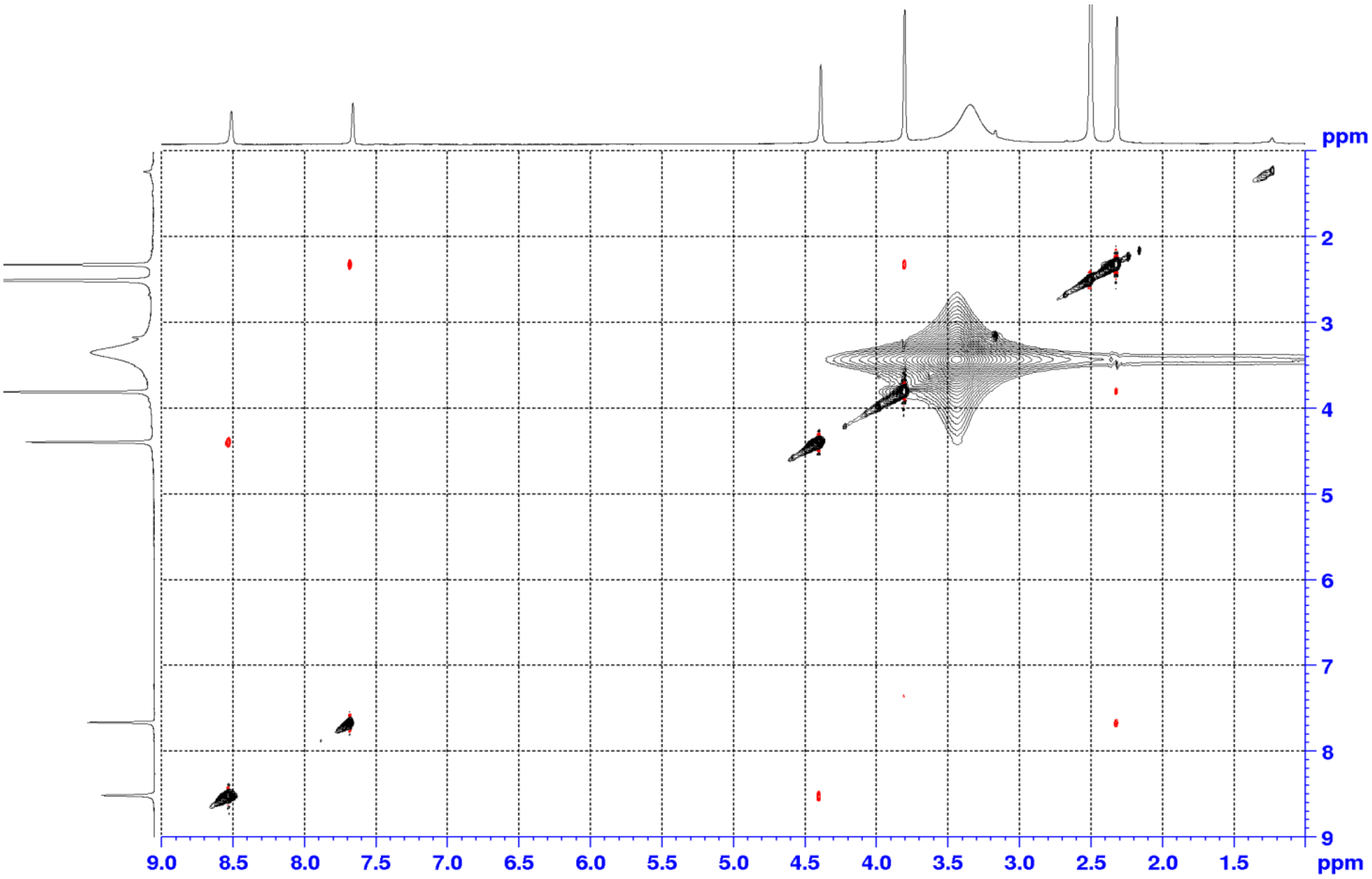

Figure S19: NOESY spectrum of **3** in DMSO-d_6

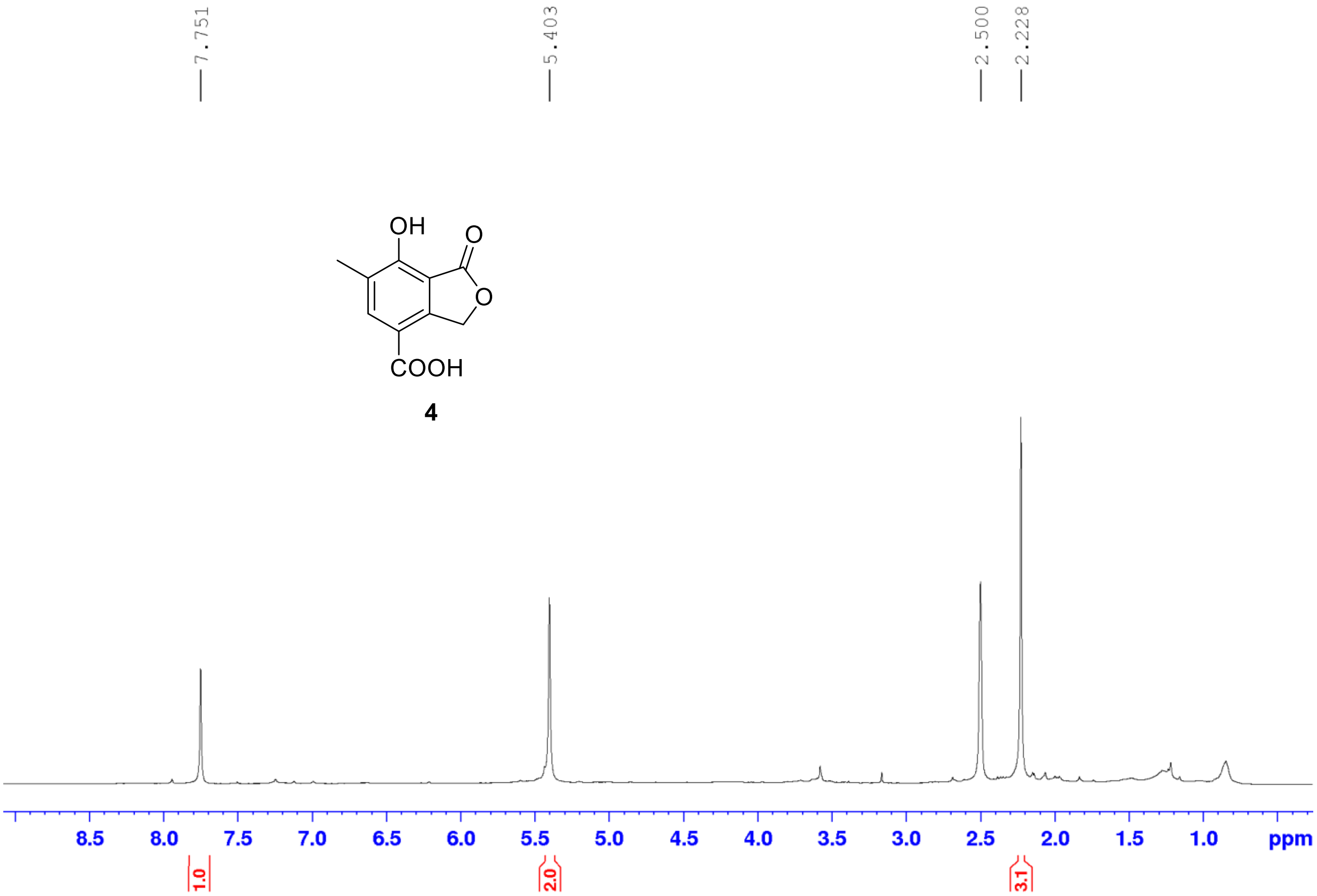

4

Figure S20: ^{1}H NMR spectrum of **4** in DMSO-d_6 (400 MHz)

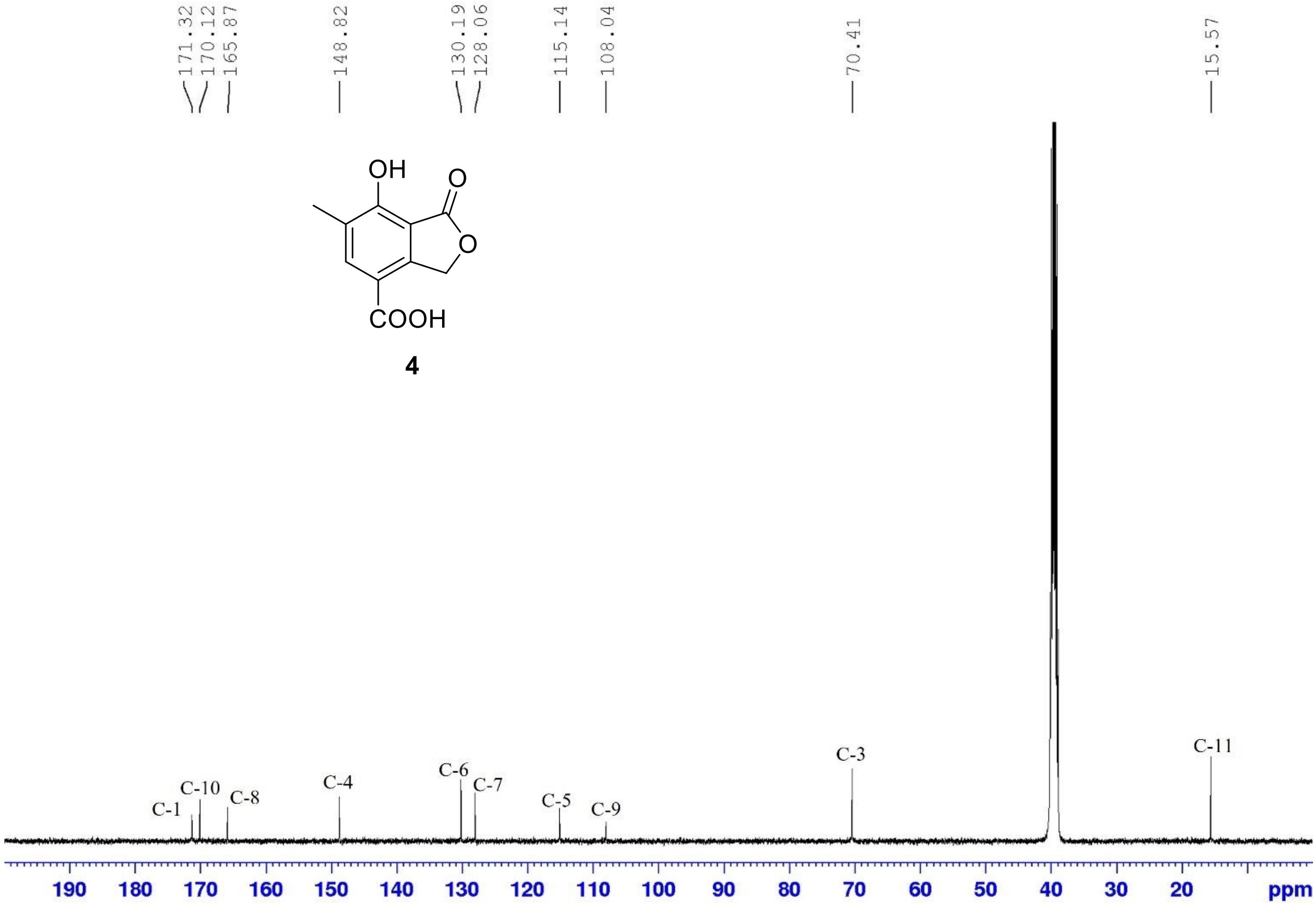

4

Figure S21: ^{13}C NMR spectrum of **4** in DMSO-d_6 (101 MHz)

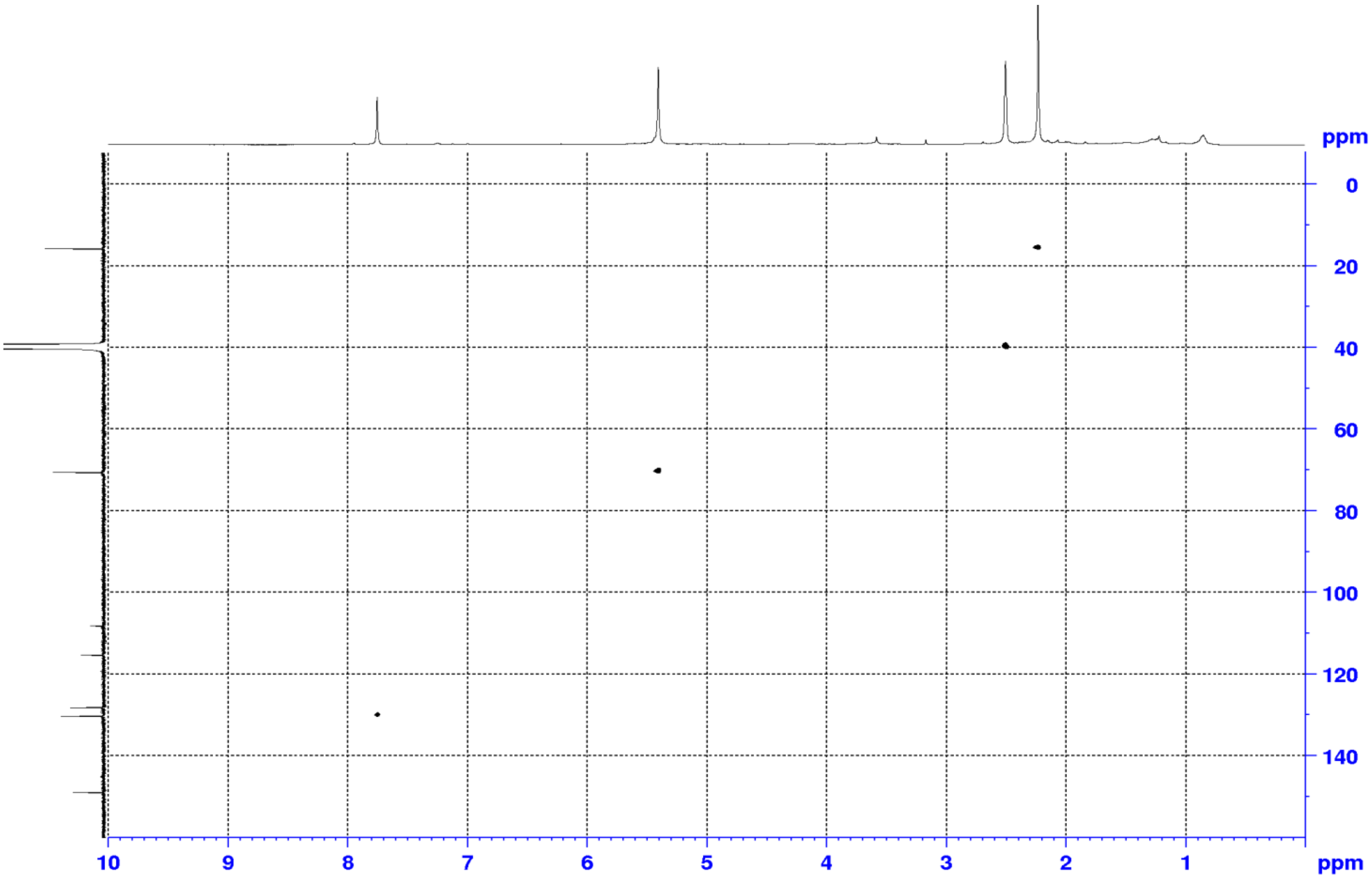

Figure S22: HSQC spectrum of **4** in DMSO-d_6

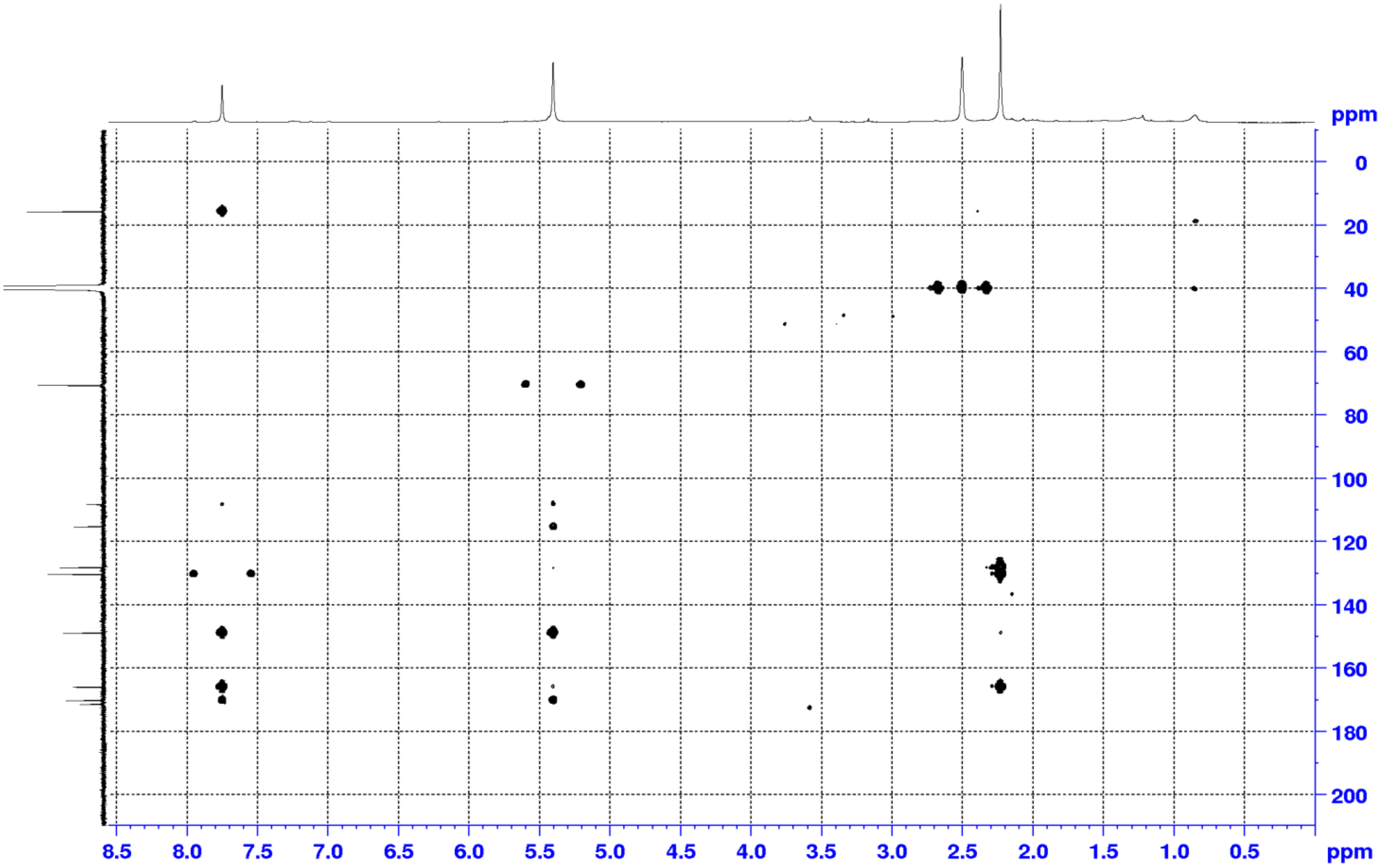

Figure S23: HMBC spectrum of **4** in DMSO-d_6

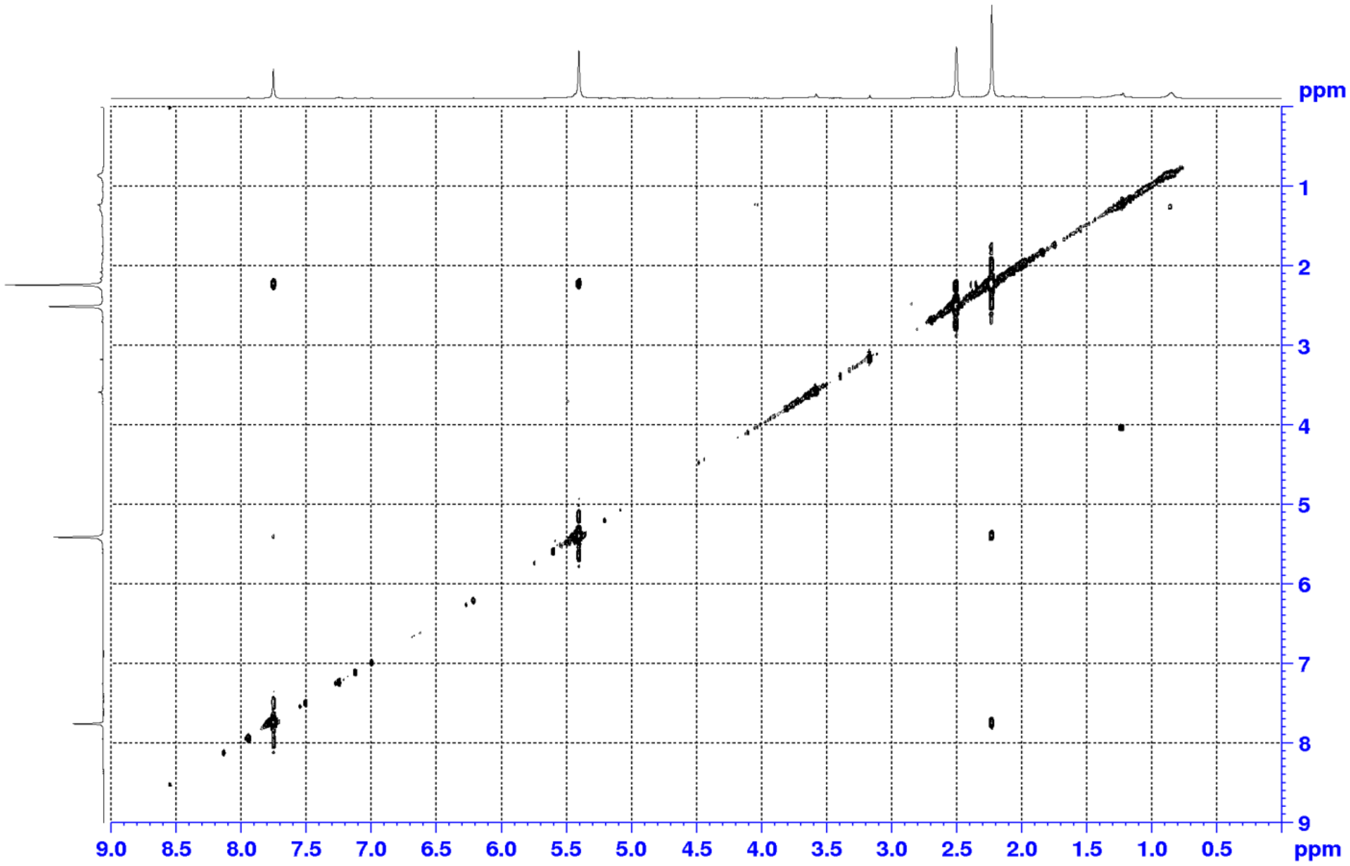

Figure S24: COSY spectrum of **4** in DMSO-d_6

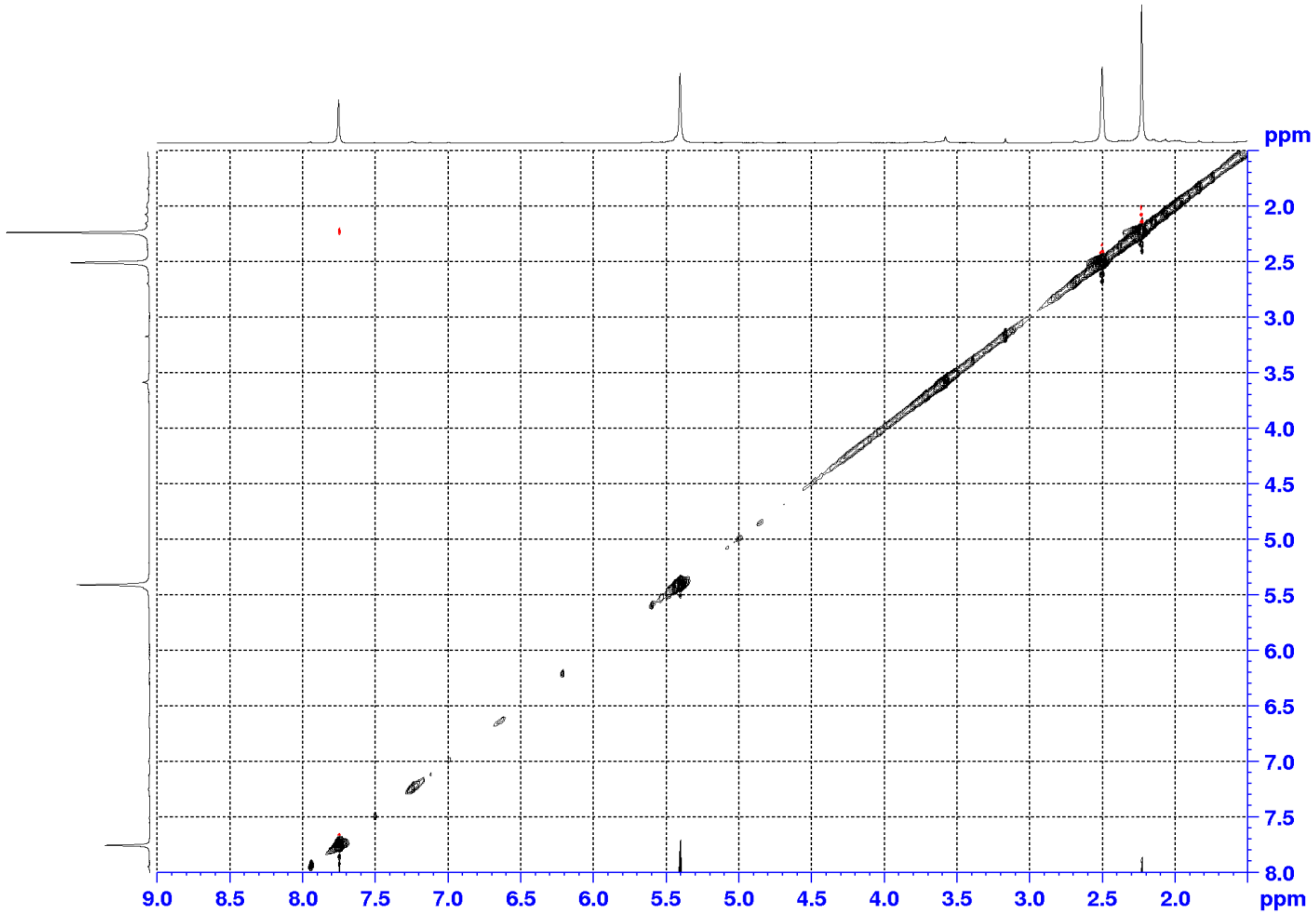

Figure S25: NOESY spectrum of **4** in DMSO-d_6

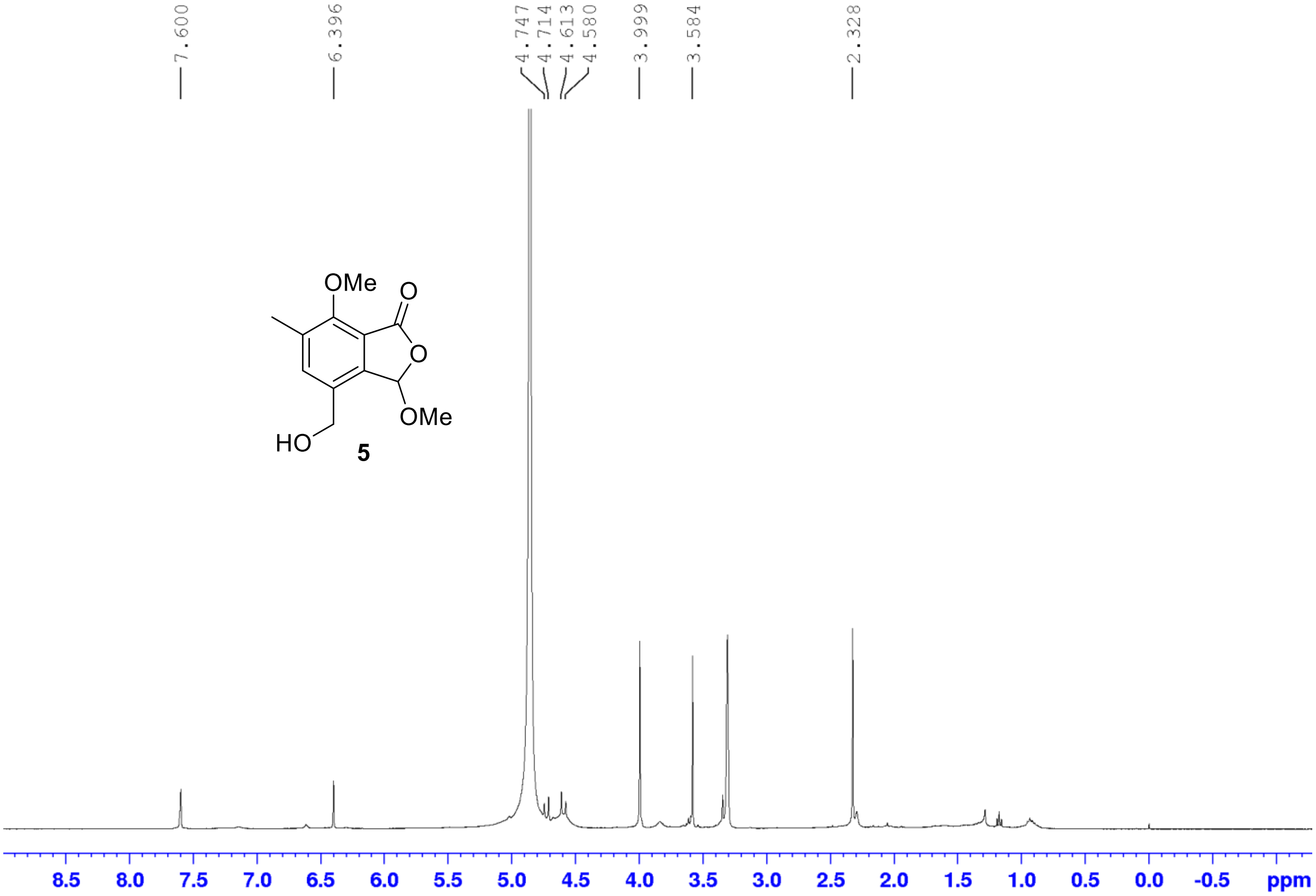

Figure S26: ^{1}H NMR spectrum of **5** in methanol-d_4 (400 MHz)

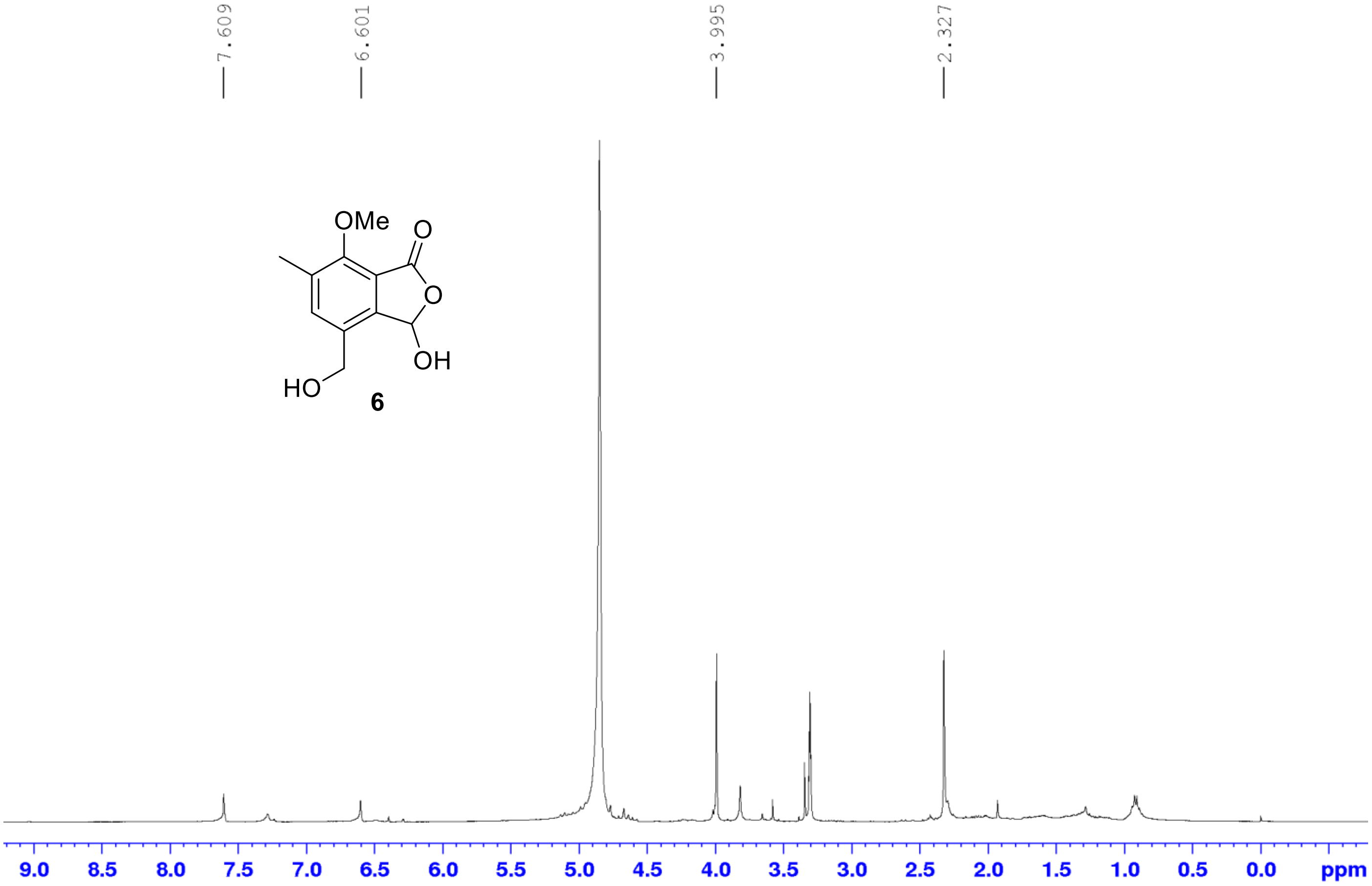

Figure S27: ^{1}H NMR spectrum of **6** in methanol-d_4 (400 MHz)

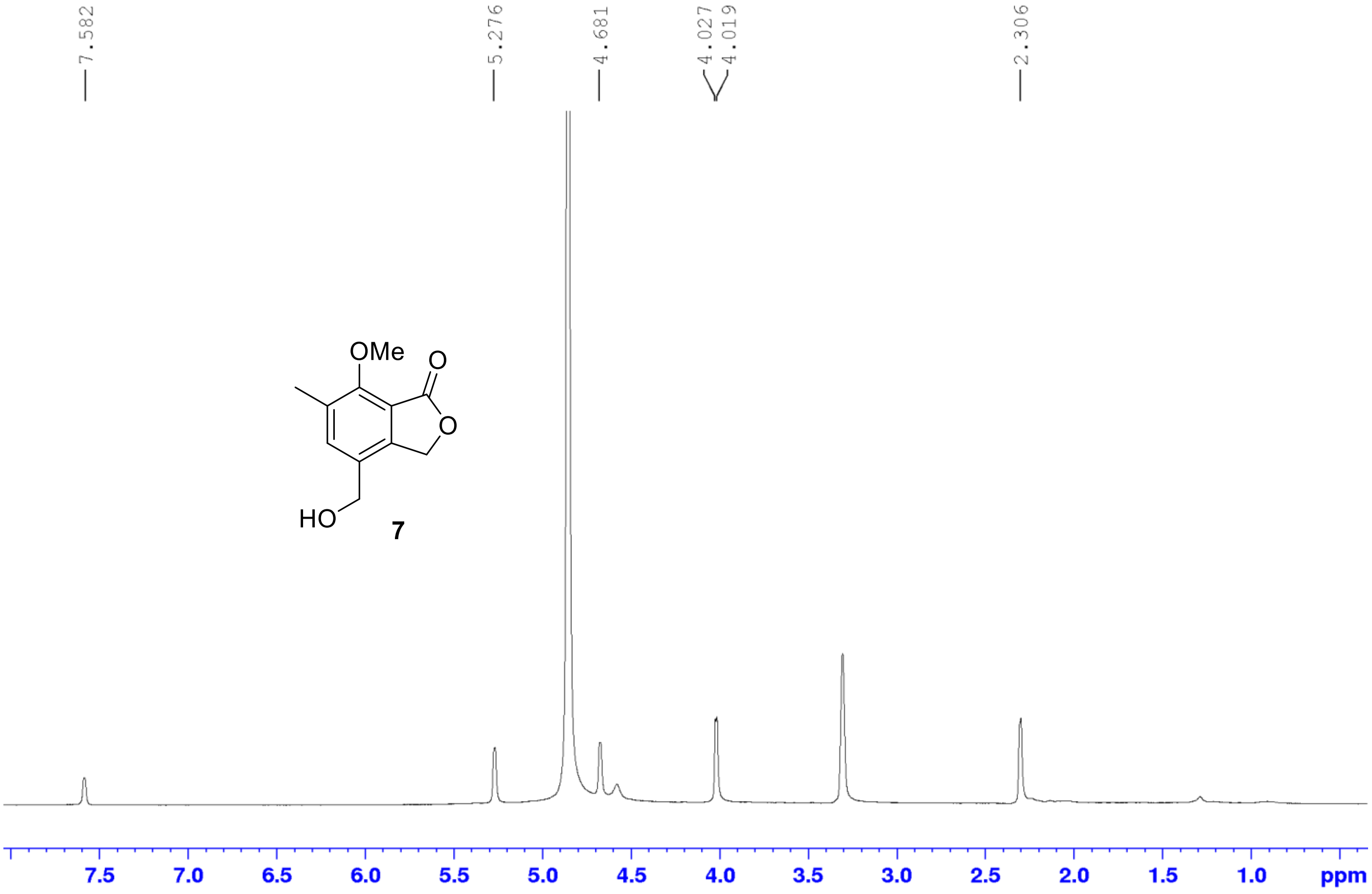

Figure S28: ^{1}H NMR spectrum of **7** in methanol-d_4 (400 MHz)

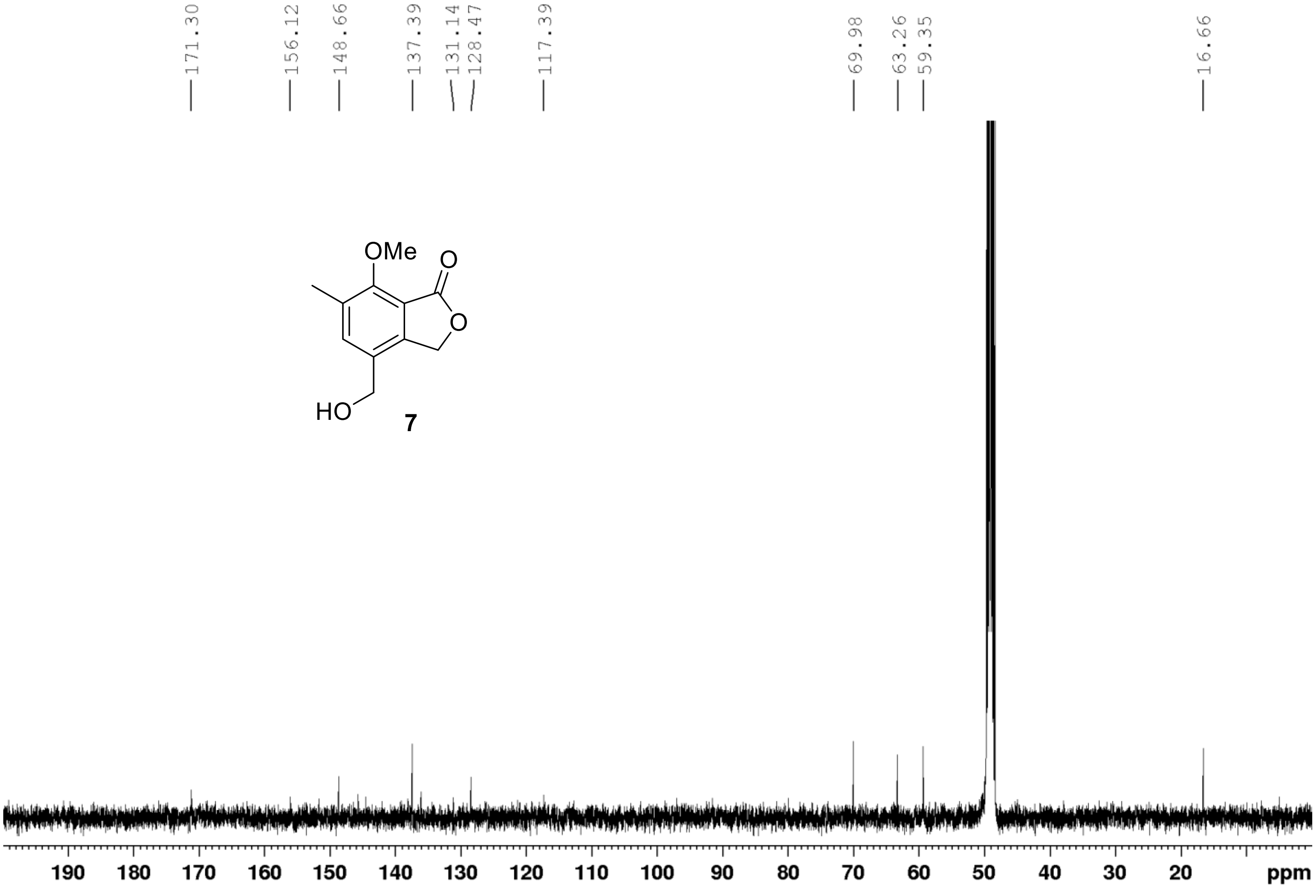

Figure S29: ^{13}C NMR spectrum of **7** in methanol-d_4 (101 MHz)

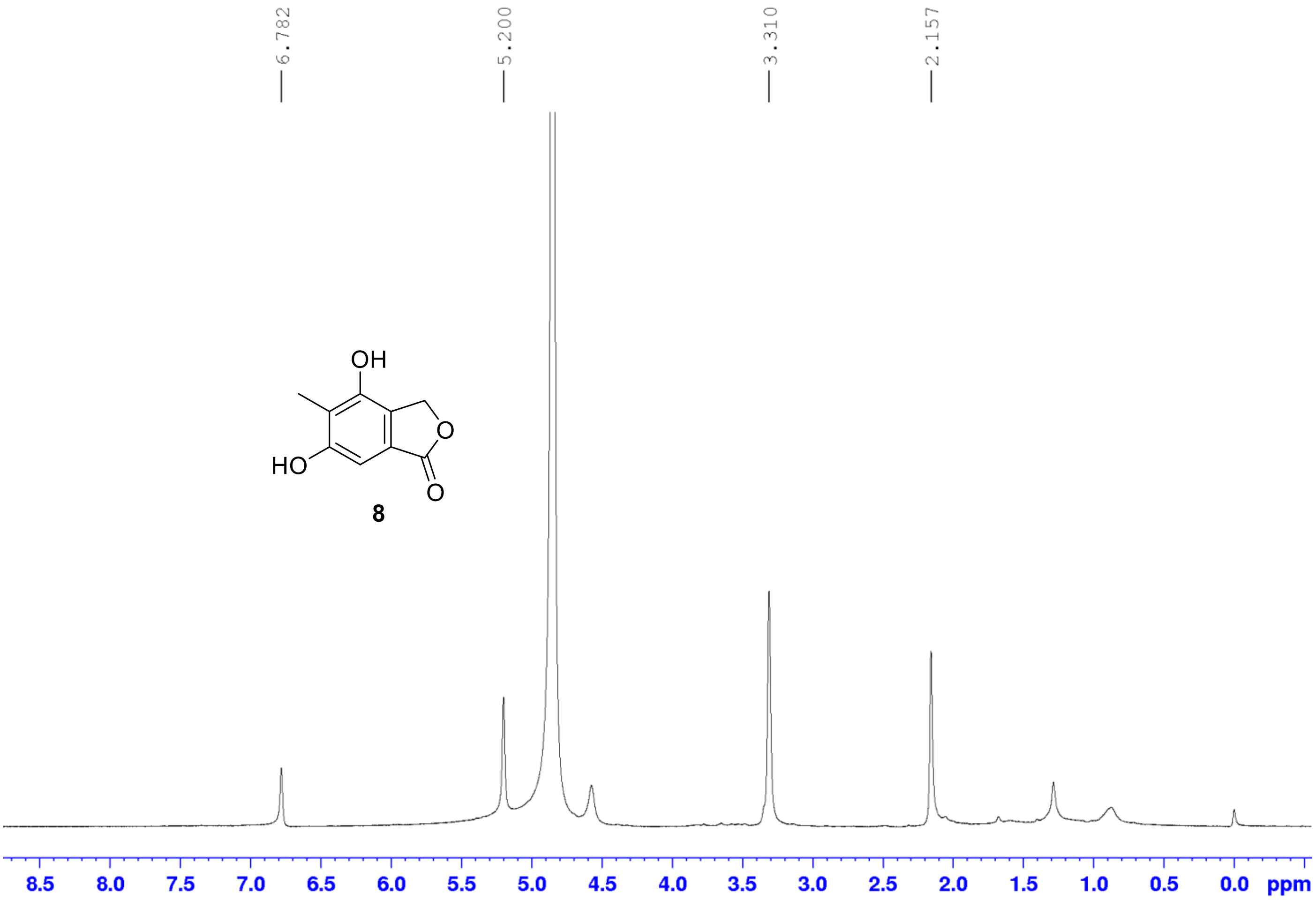

Figure S30: ^{1}H NMR spectrum of **8** in methanol-d_4 (400 MHz)

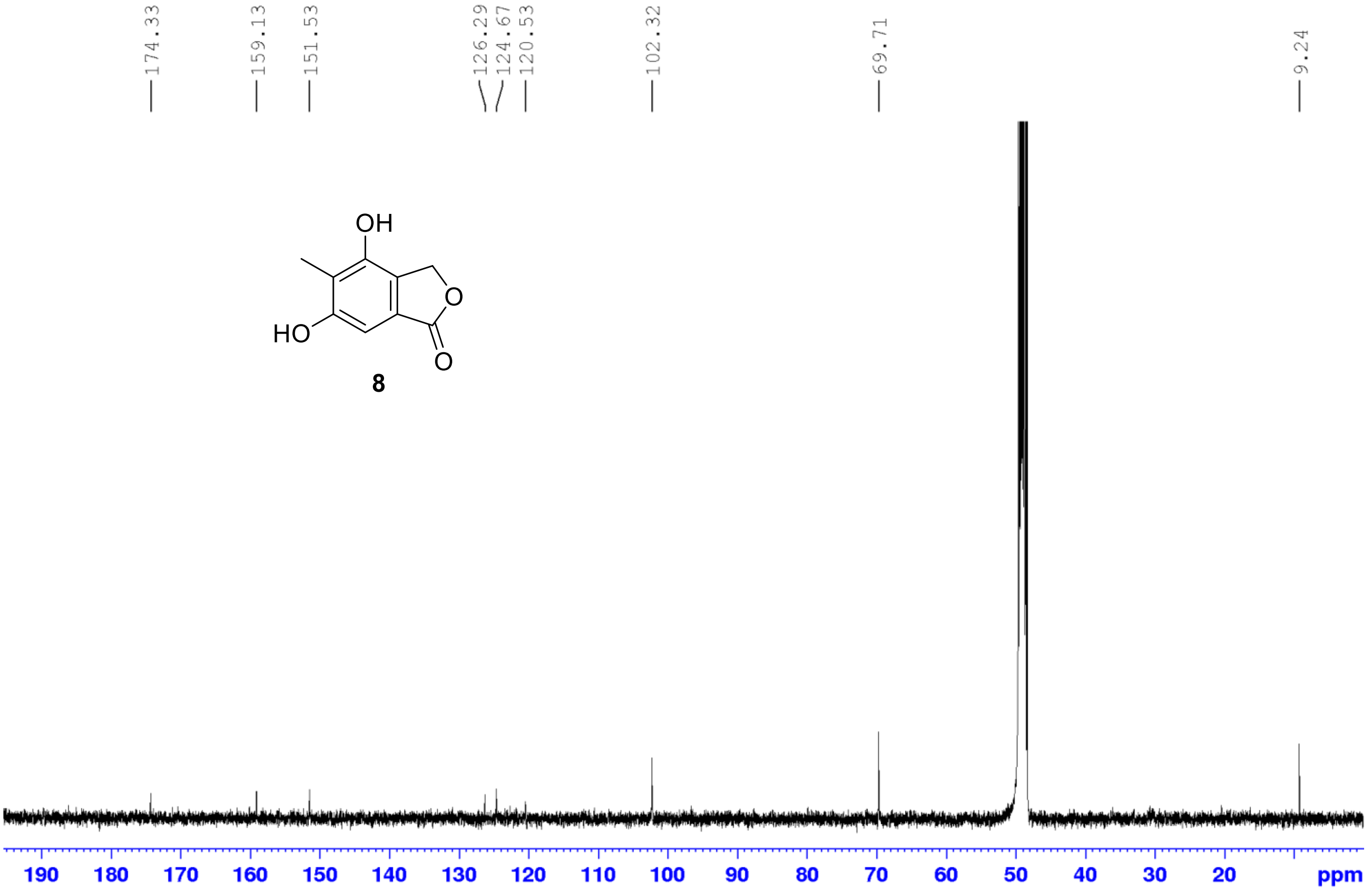

Figure S31: ^{13}C NMR spectrum of **8** in methanol-d_4 (101 MHz)

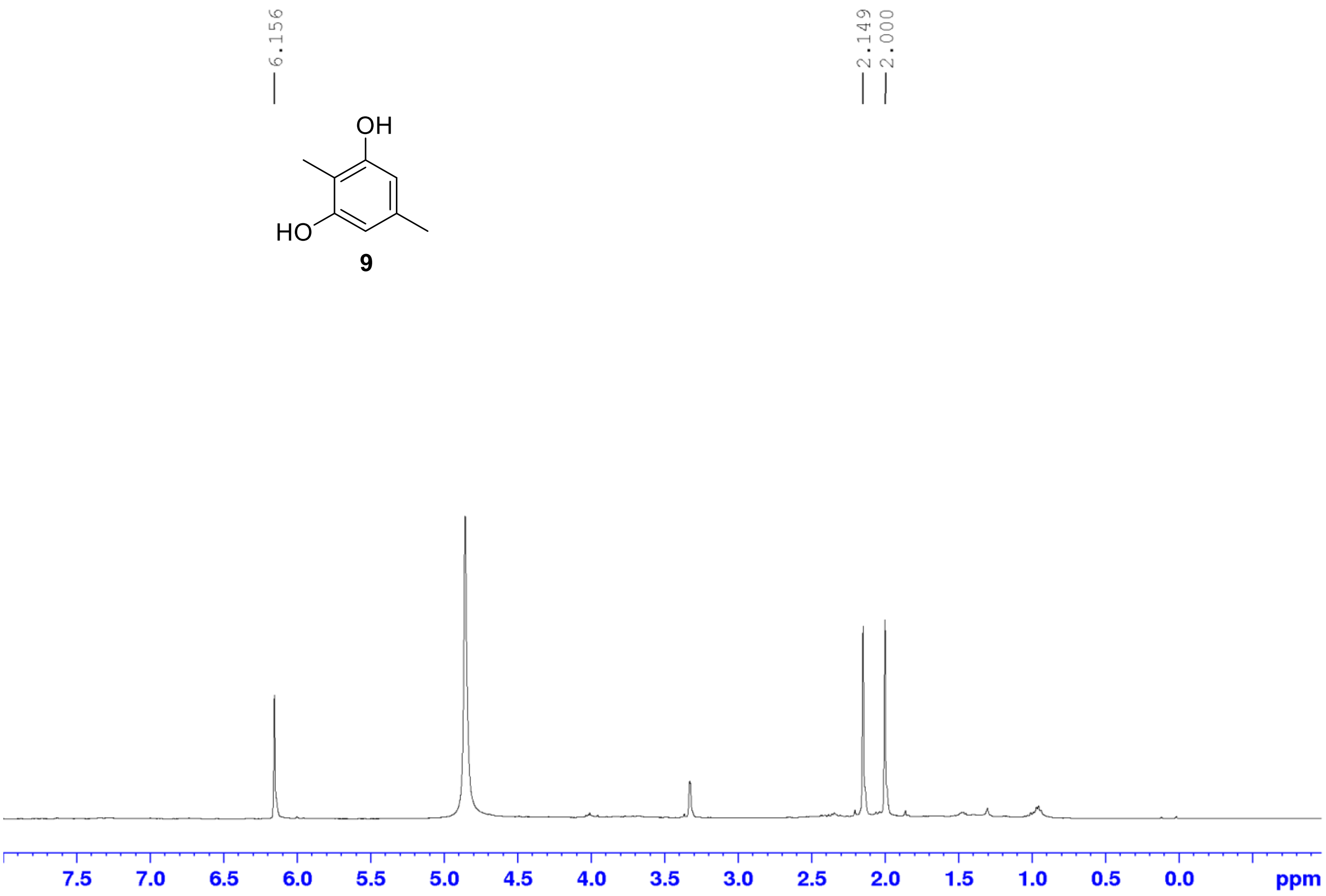

Figure S32: ^{1}H NMR spectrum of **9** in methanol-d_4 (400 MHz)

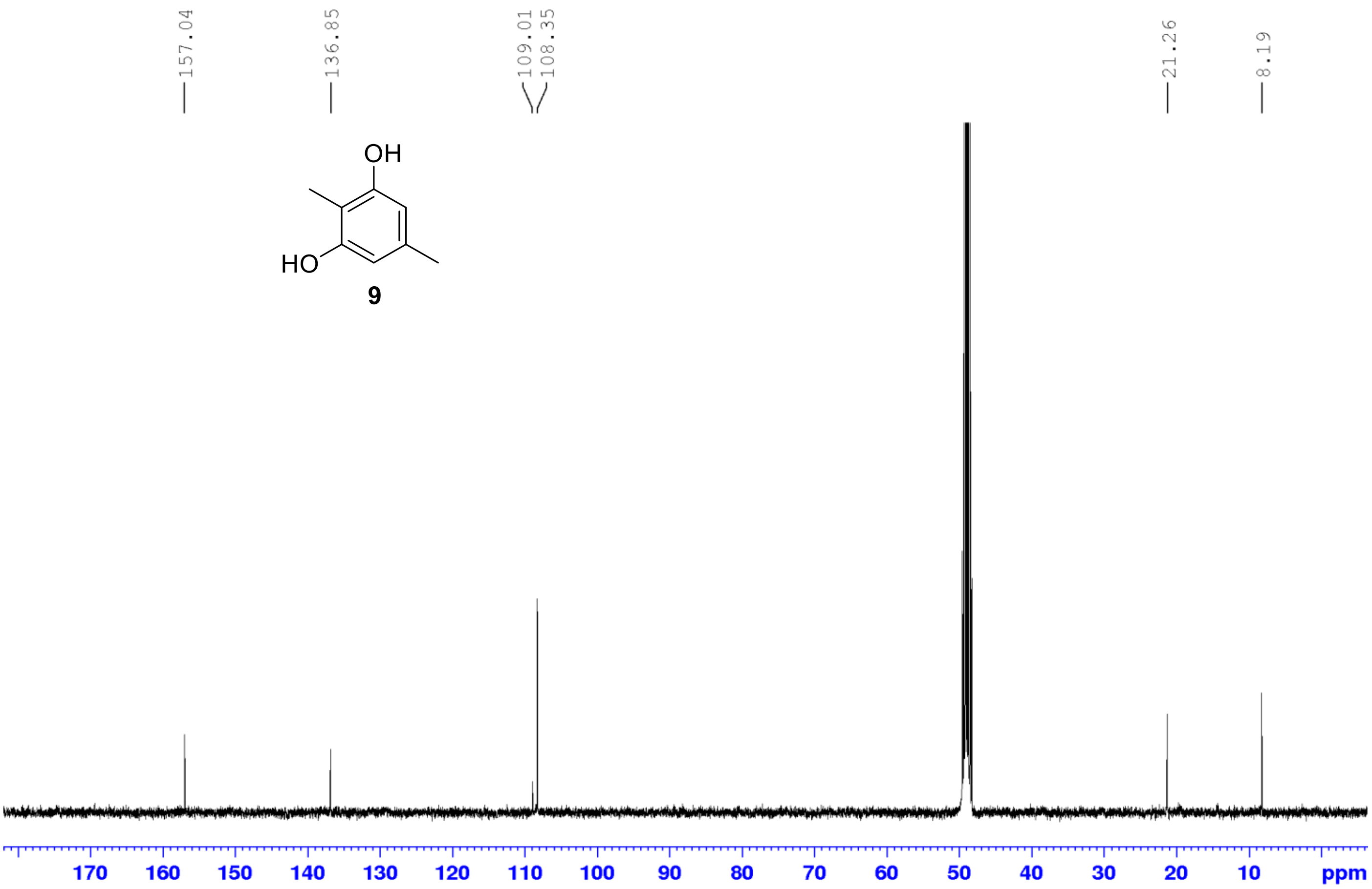

Figure S33: ^{13}C NMR spectrum of **9** in methanol-d_4 (101 MHz)

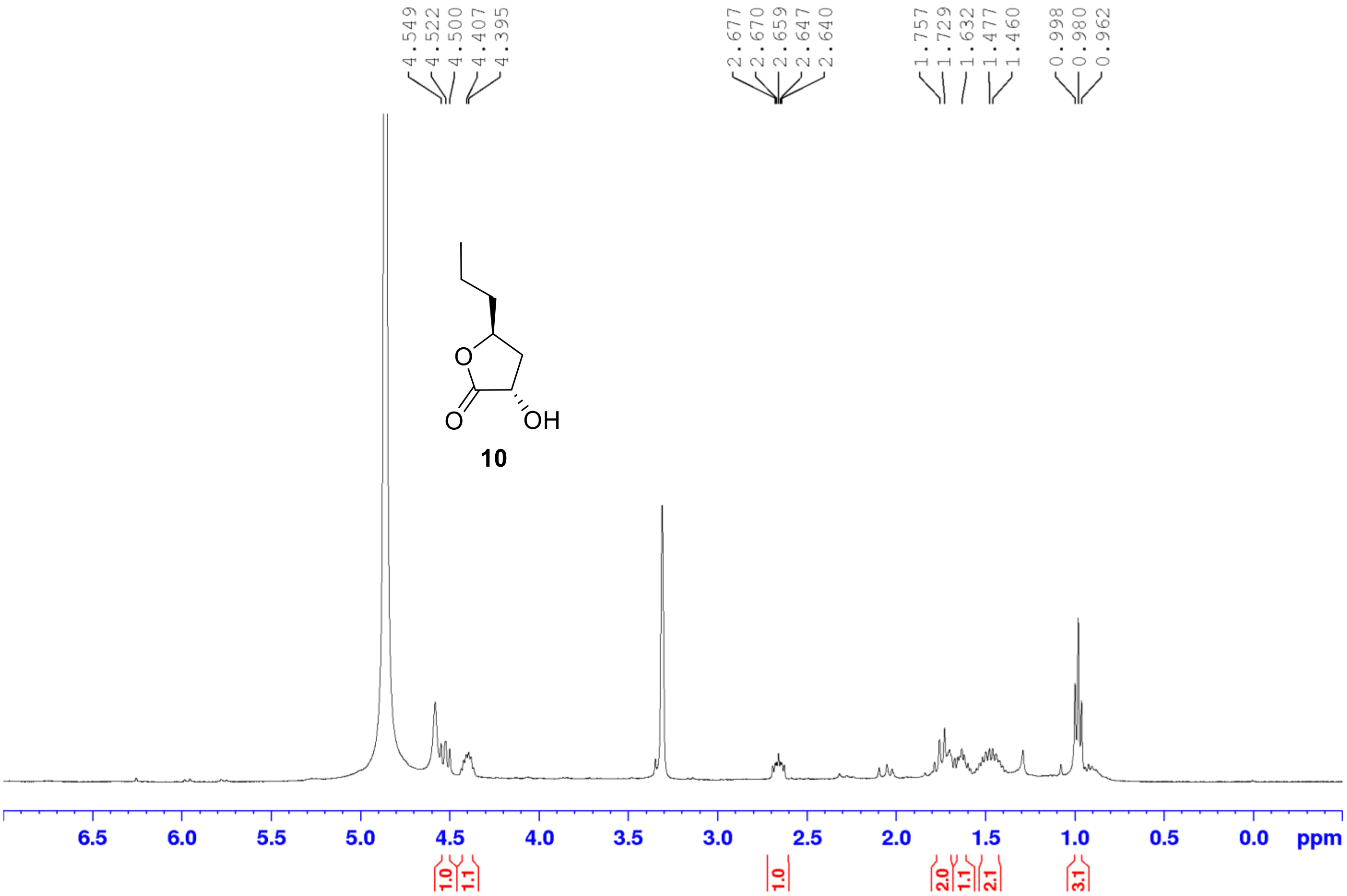

Figure S34: ^{1}H NMR spectrum of **10** in methanol-d_4 (400 MHz)

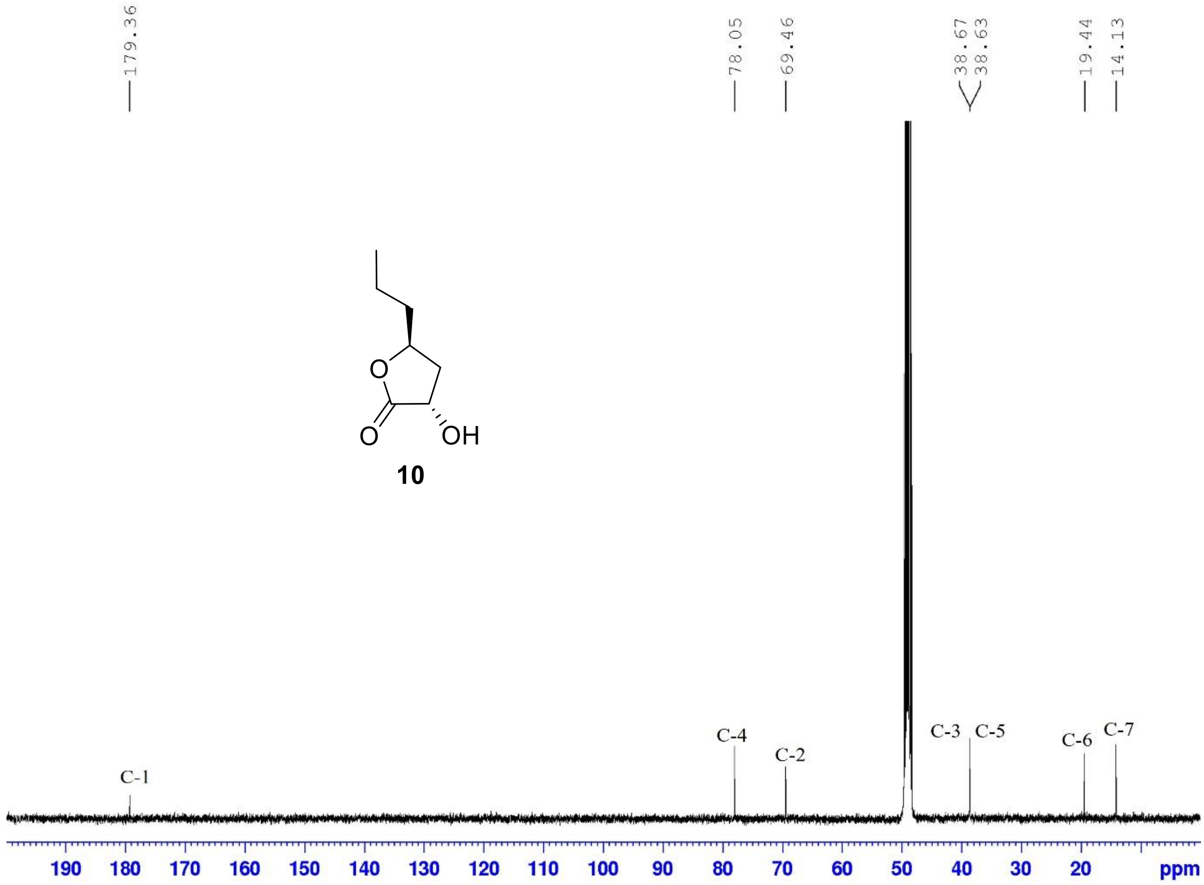

Figure S35: ^{13}C NMR spectrum of **10** in methanol-d_4 (101 MHz)

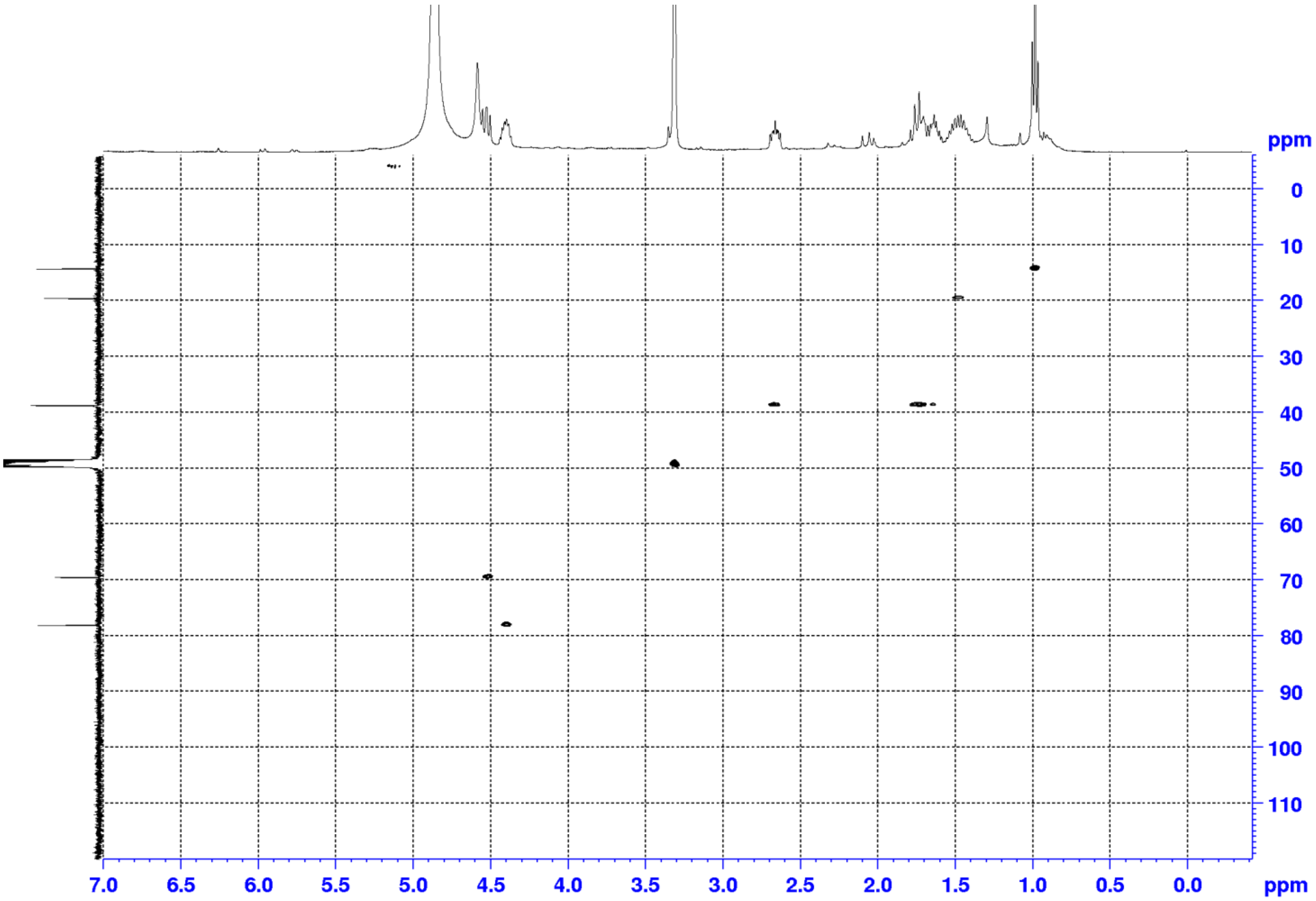

Figure S36: HSQC spectrum of **10** in methanol-d_4

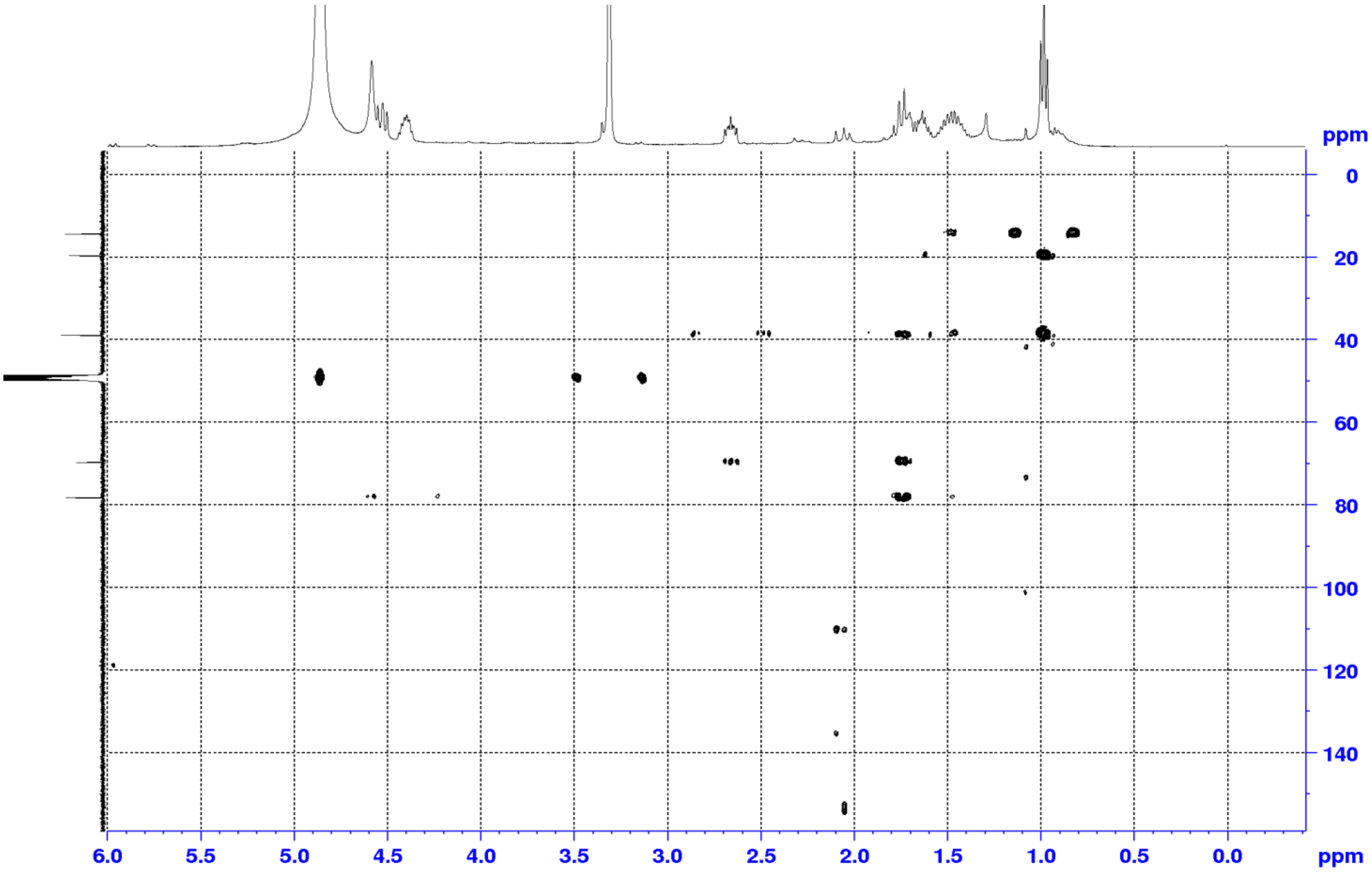

Figure S37: HMBC spectrum of **10** in methanol-d_4

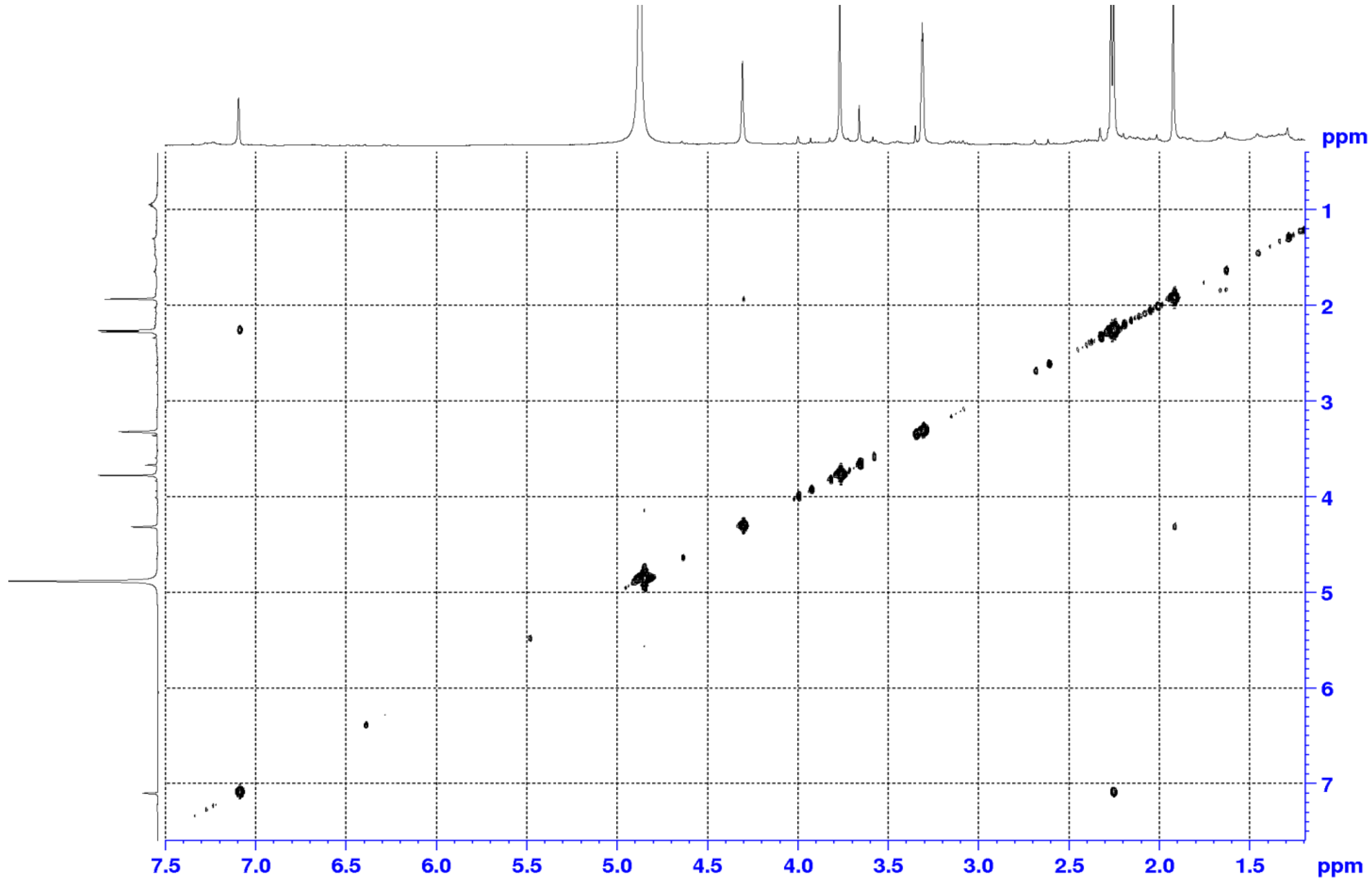

Figure S38: COSY spectrum of **10** in methanol-d_4

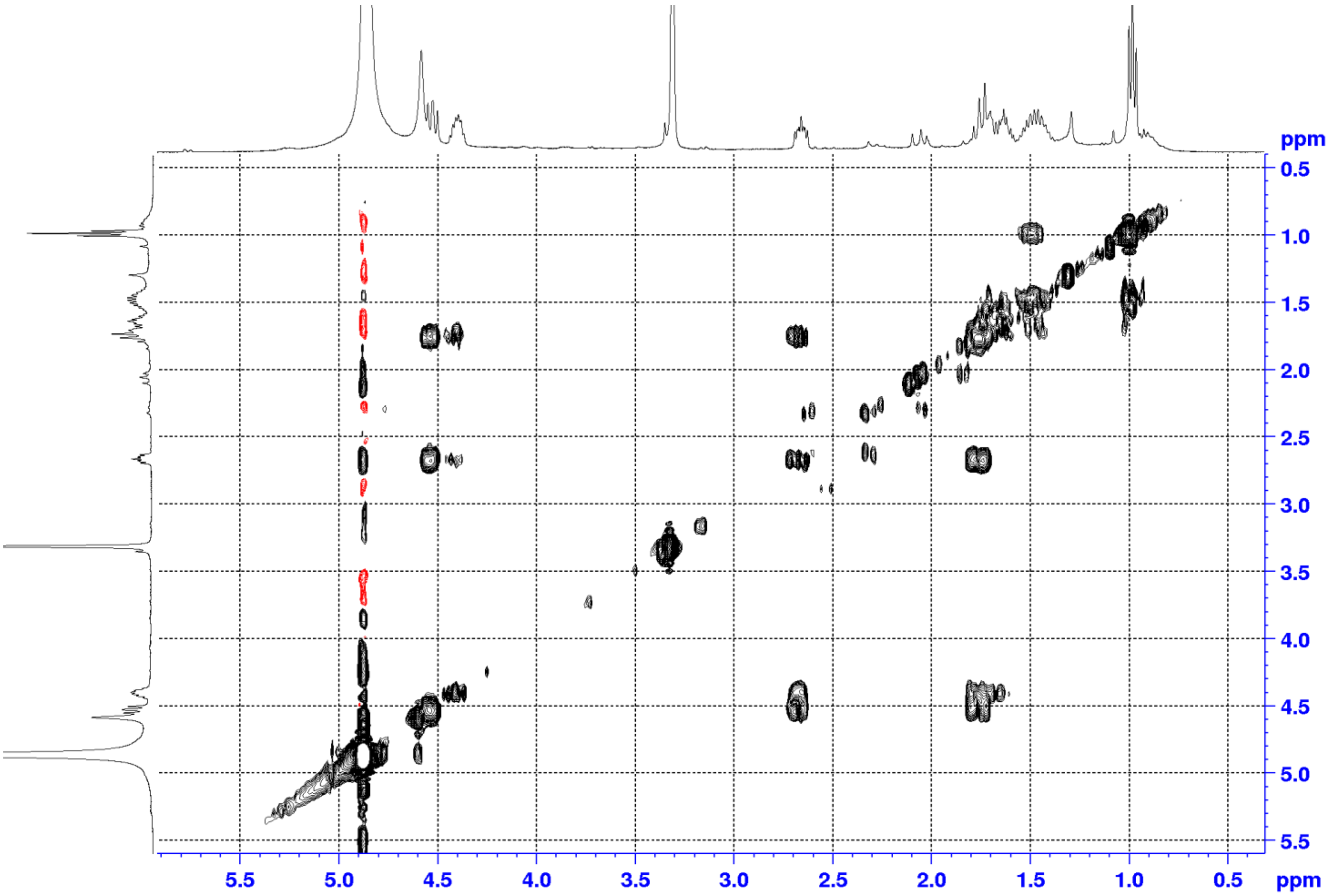

Figure S39: NOESY spectrum of **10** in methanol-d_4

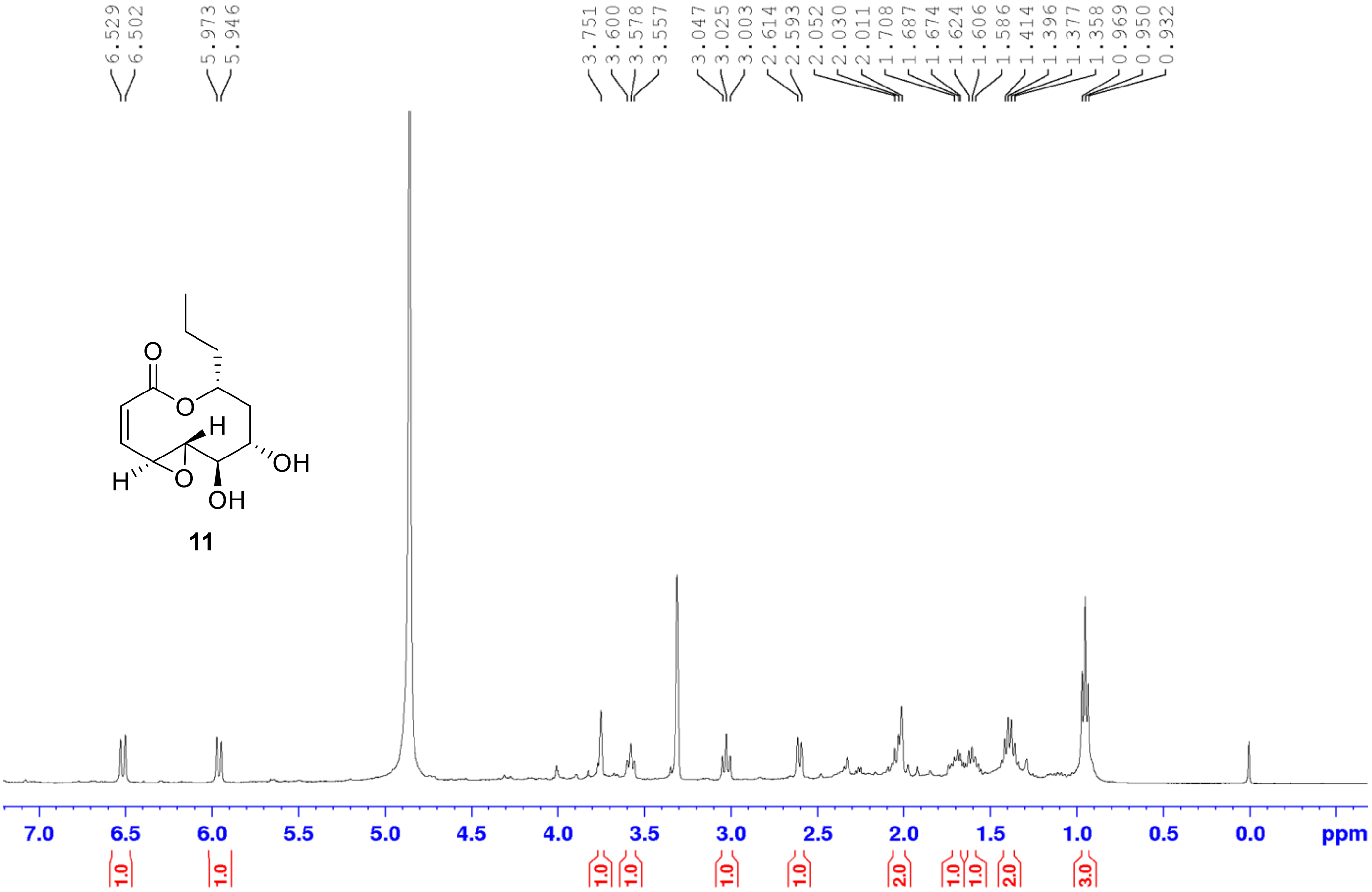

Figure S40: ^{1}H NMR spectrum of **11** in methanol-d_4 (400 MHz)

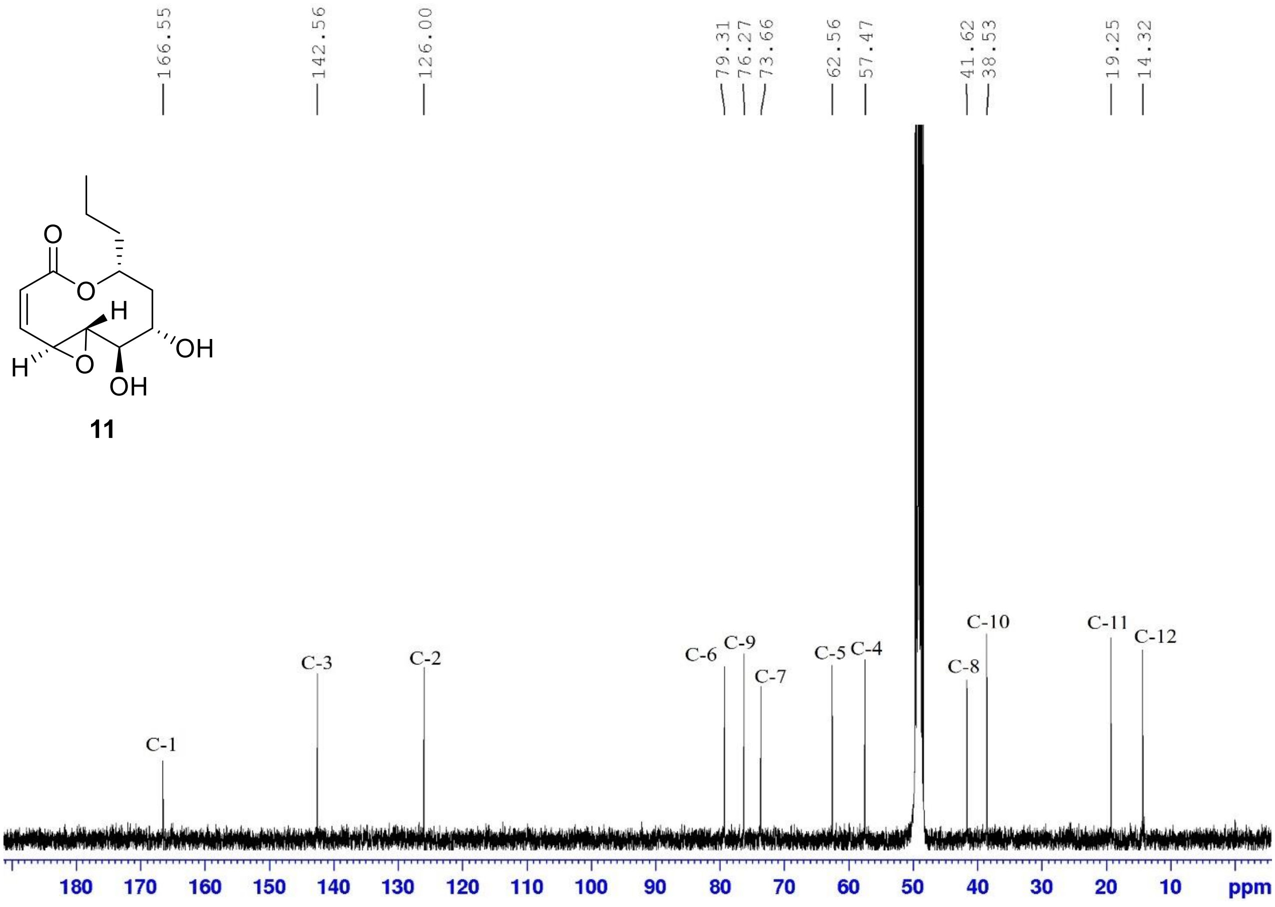

Figure S41: ^{13}C NMR spectrum of **11** in methanol-d_4 (101 MHz)

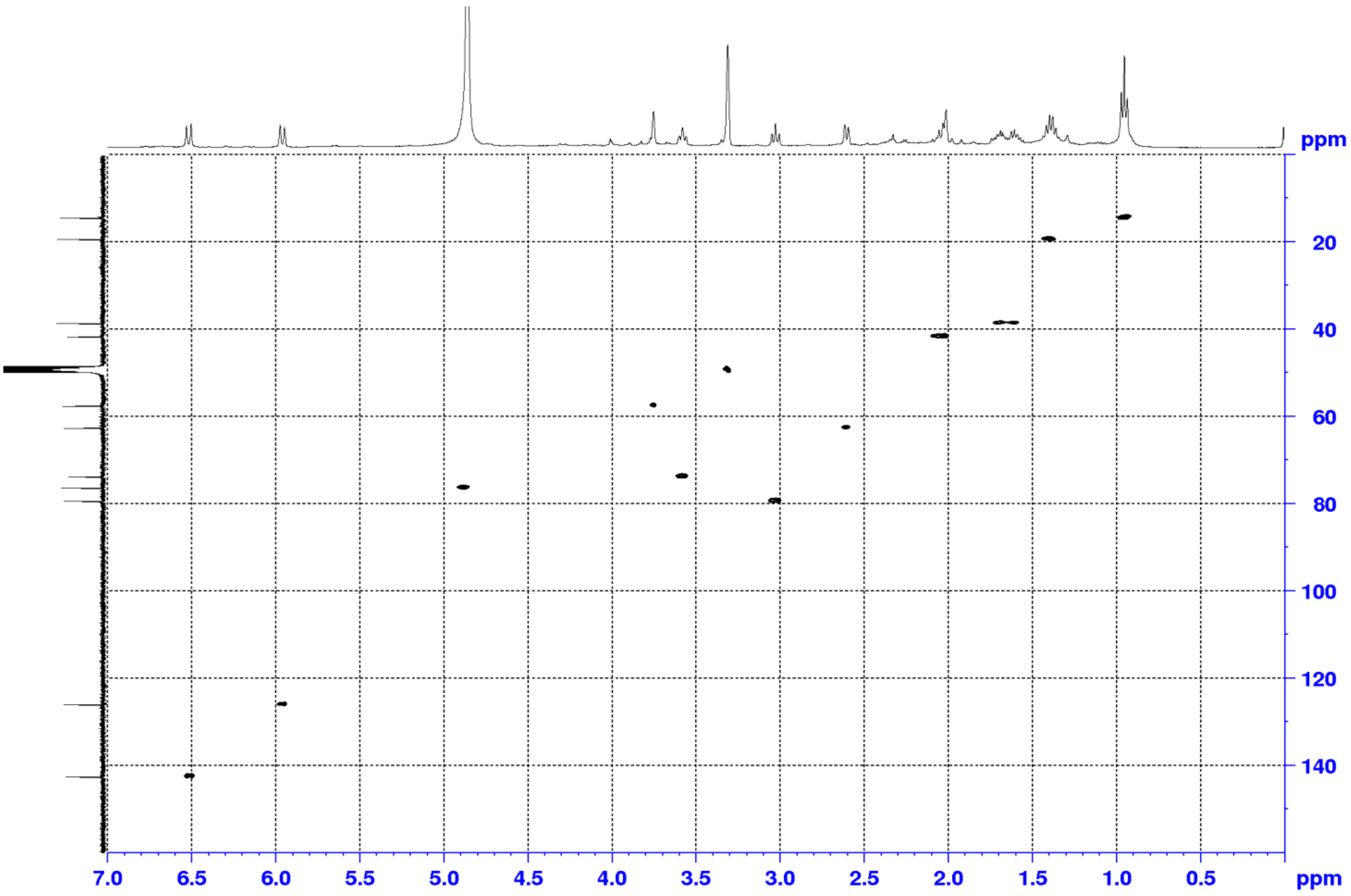

Figure S42: HSQC spectrum of **11** in methanol-d_4

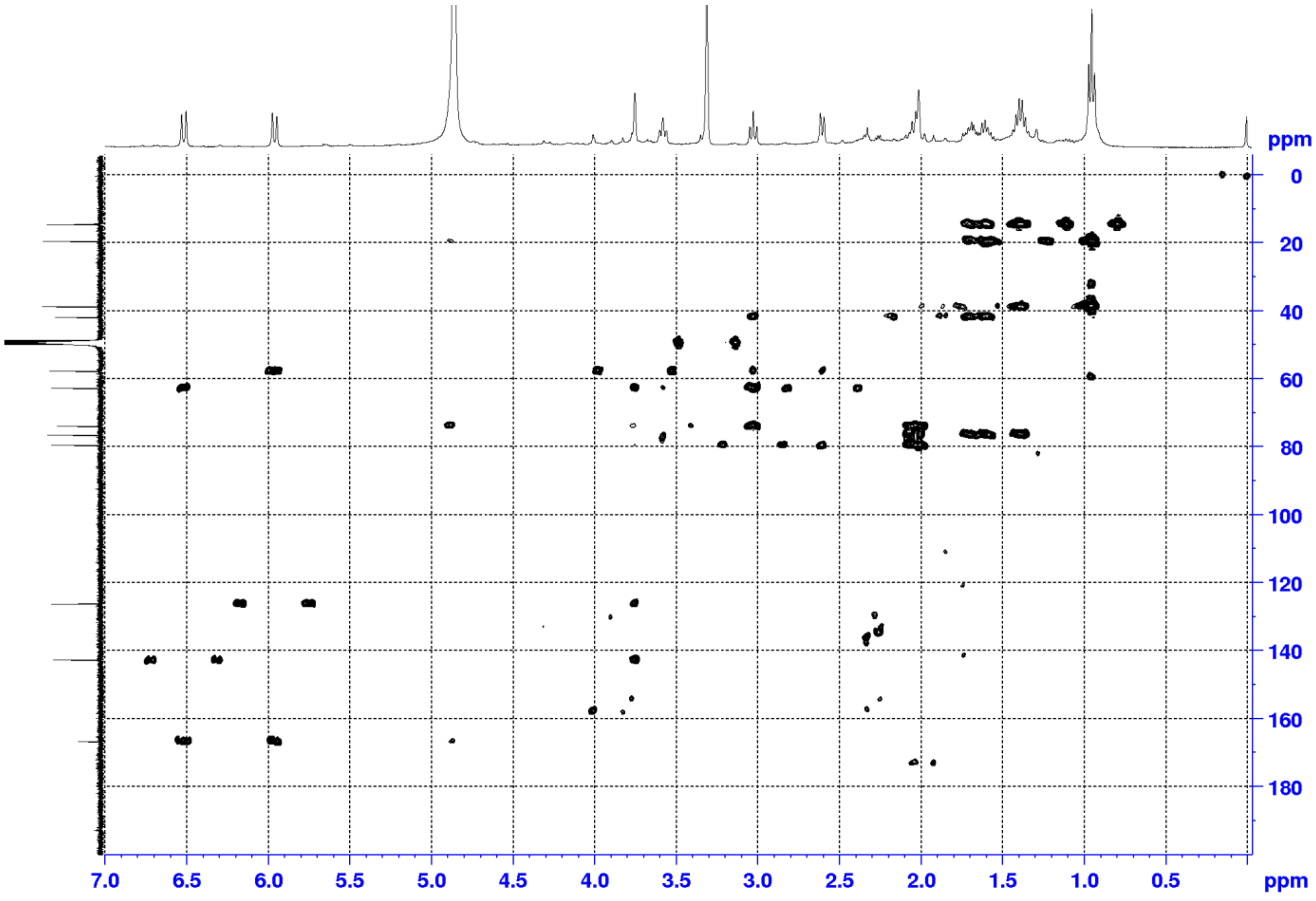

Figure S43: HMBC spectrum of **11** in methanol-d_4

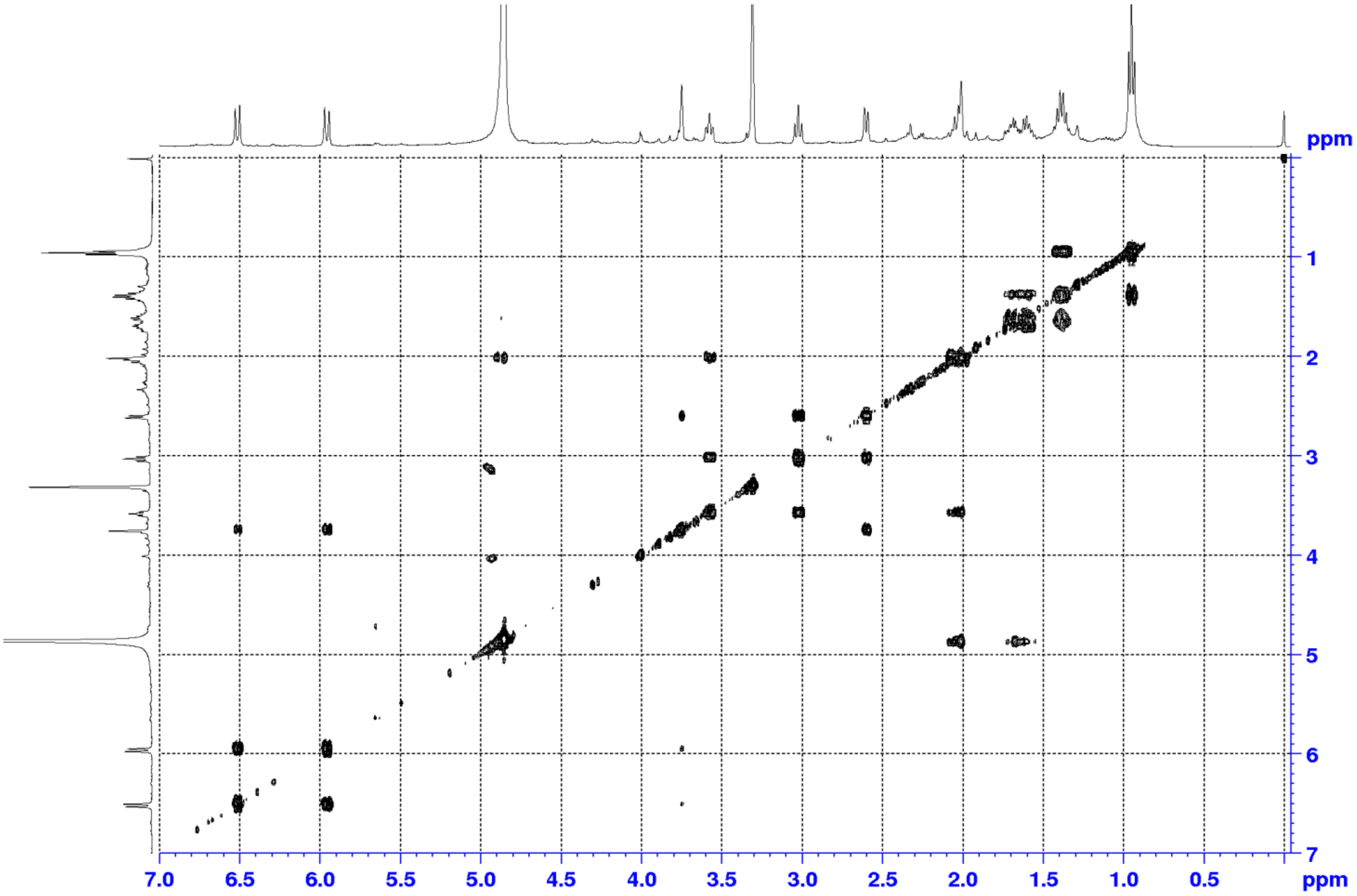

Figure S44: COSY spectrum of **11** in methanol-d_4

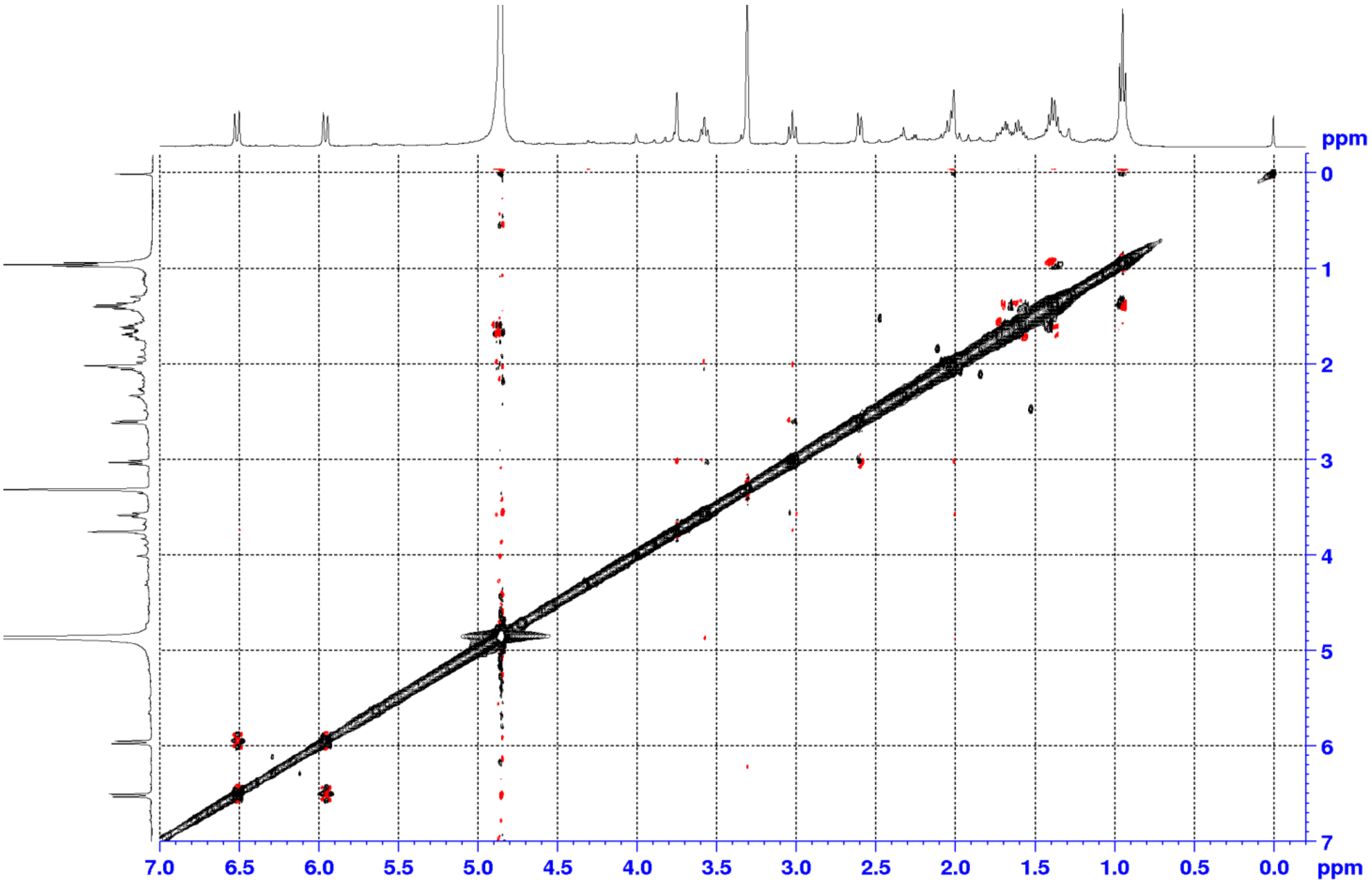

Figure S45: NOESY spectrum of **11** in methanol-d_4

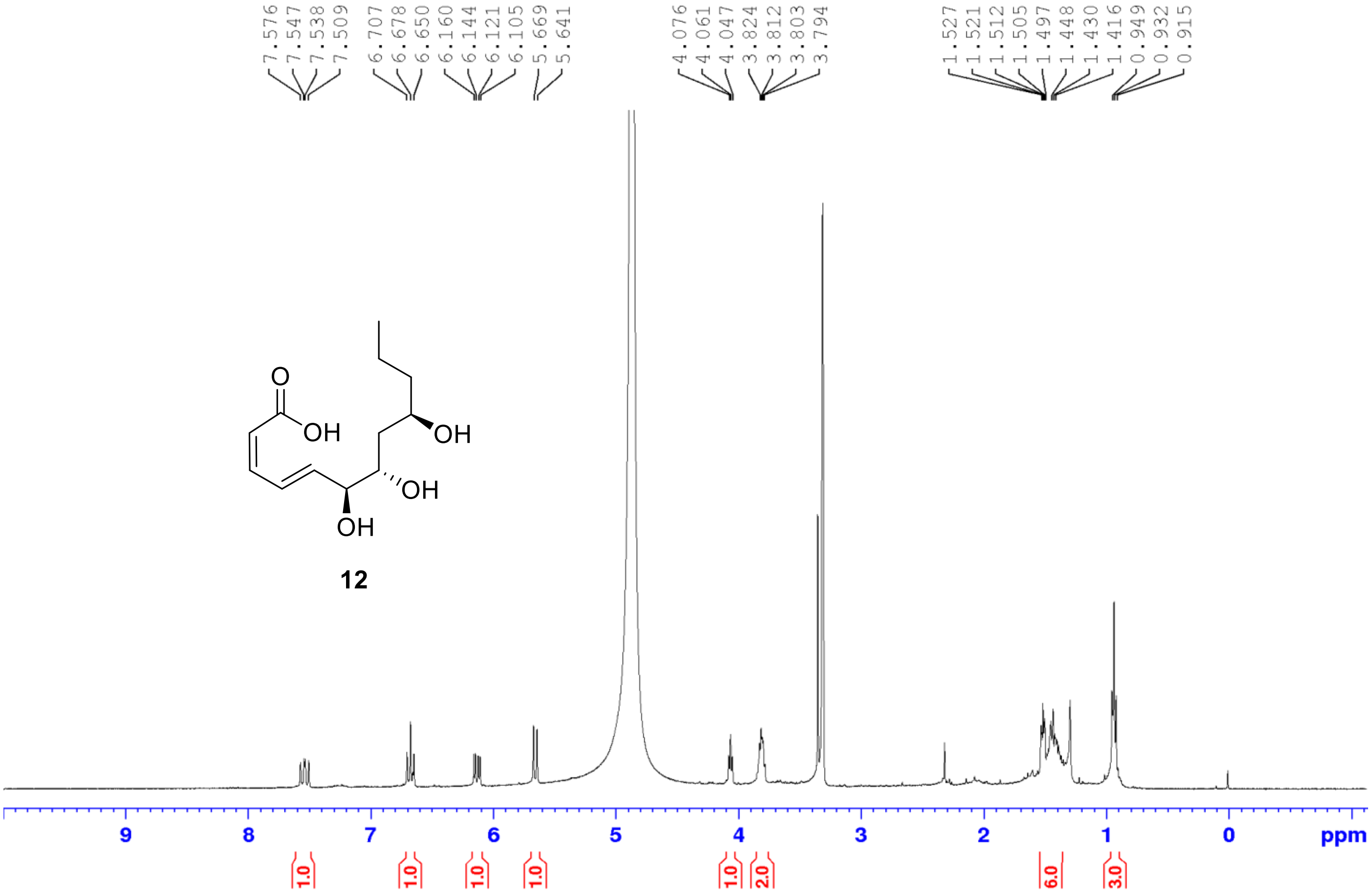

Figure S46: ^{1}H NMR spectrum of **12** in methanol-d_4 (400 MHz)

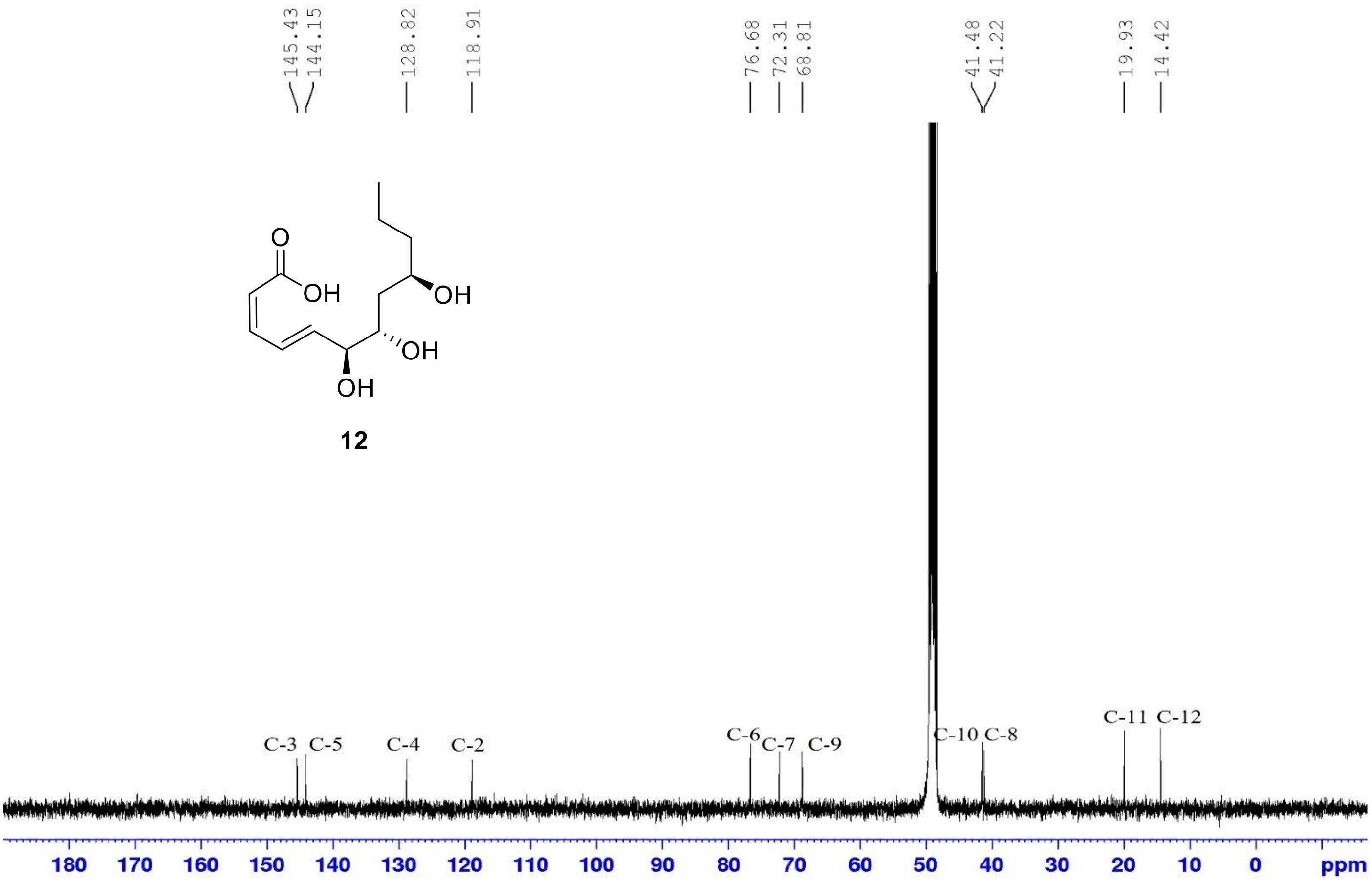

Figure S47: ^{13}C NMR spectrum of **12** in methanol-d_4 (101 MHz)

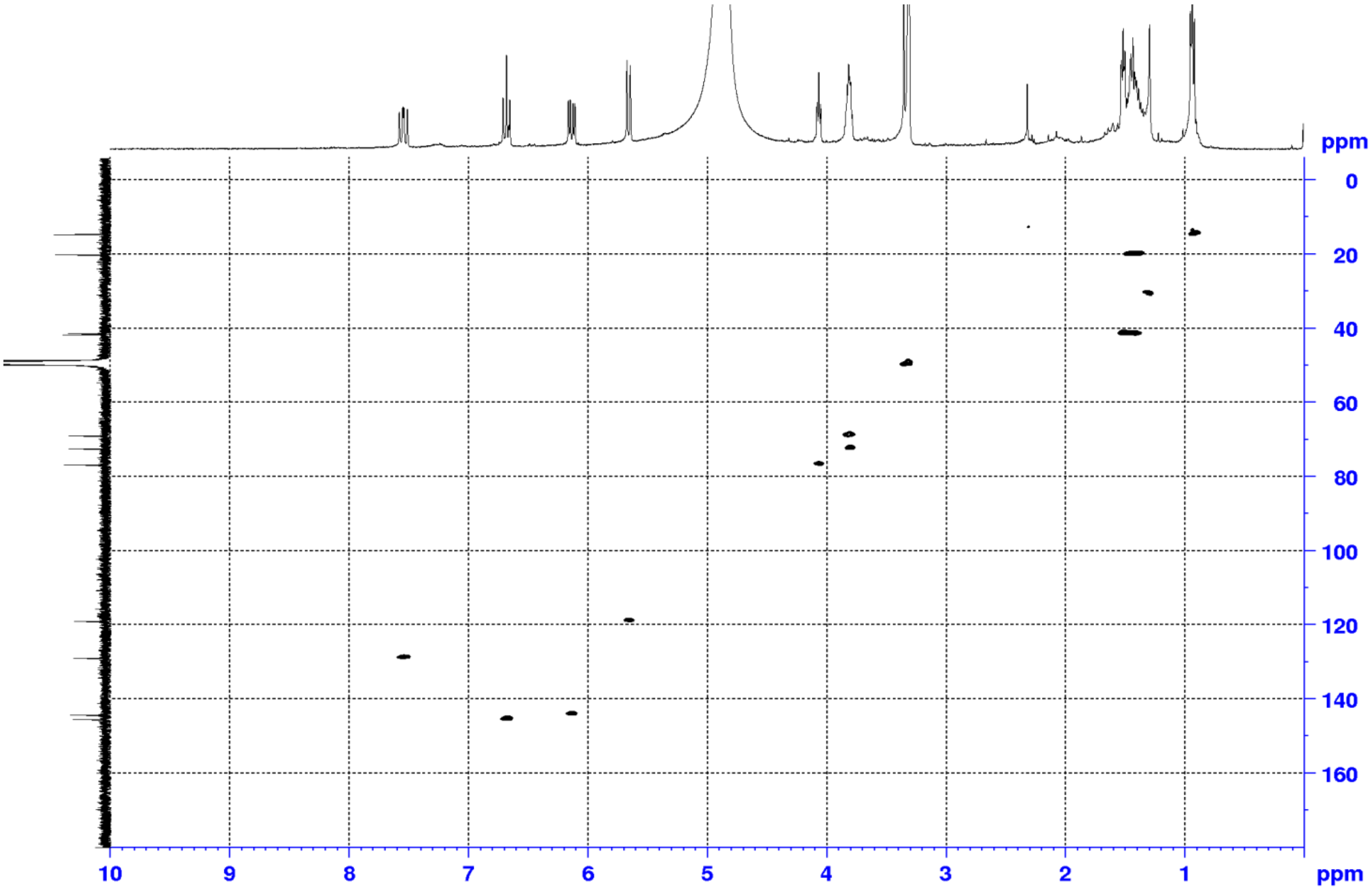

Figure S48: HSQC spectrum of **12** in methanol-d_4

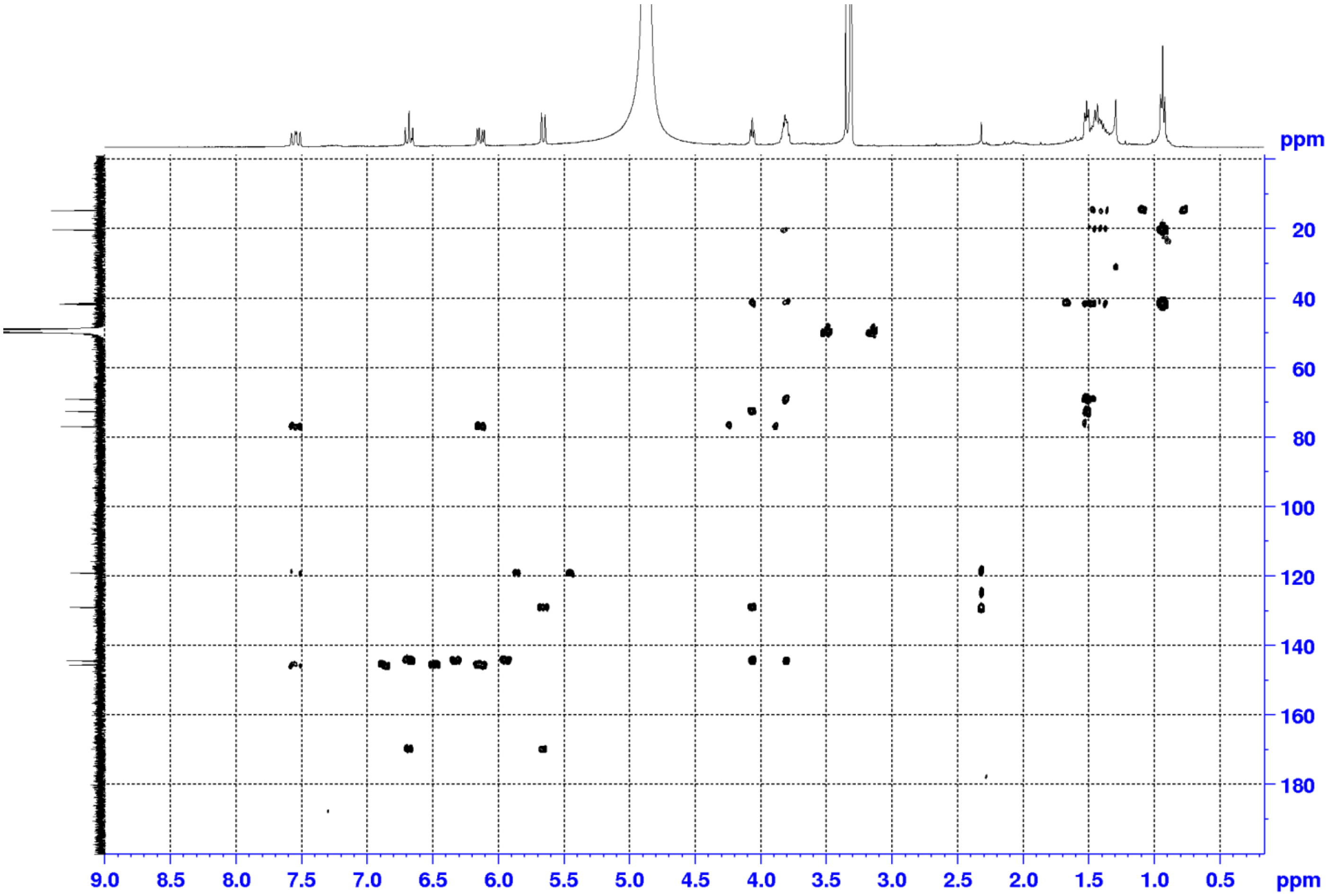

Figure S49: HMBC spectrum of **12** in methanol-d_4

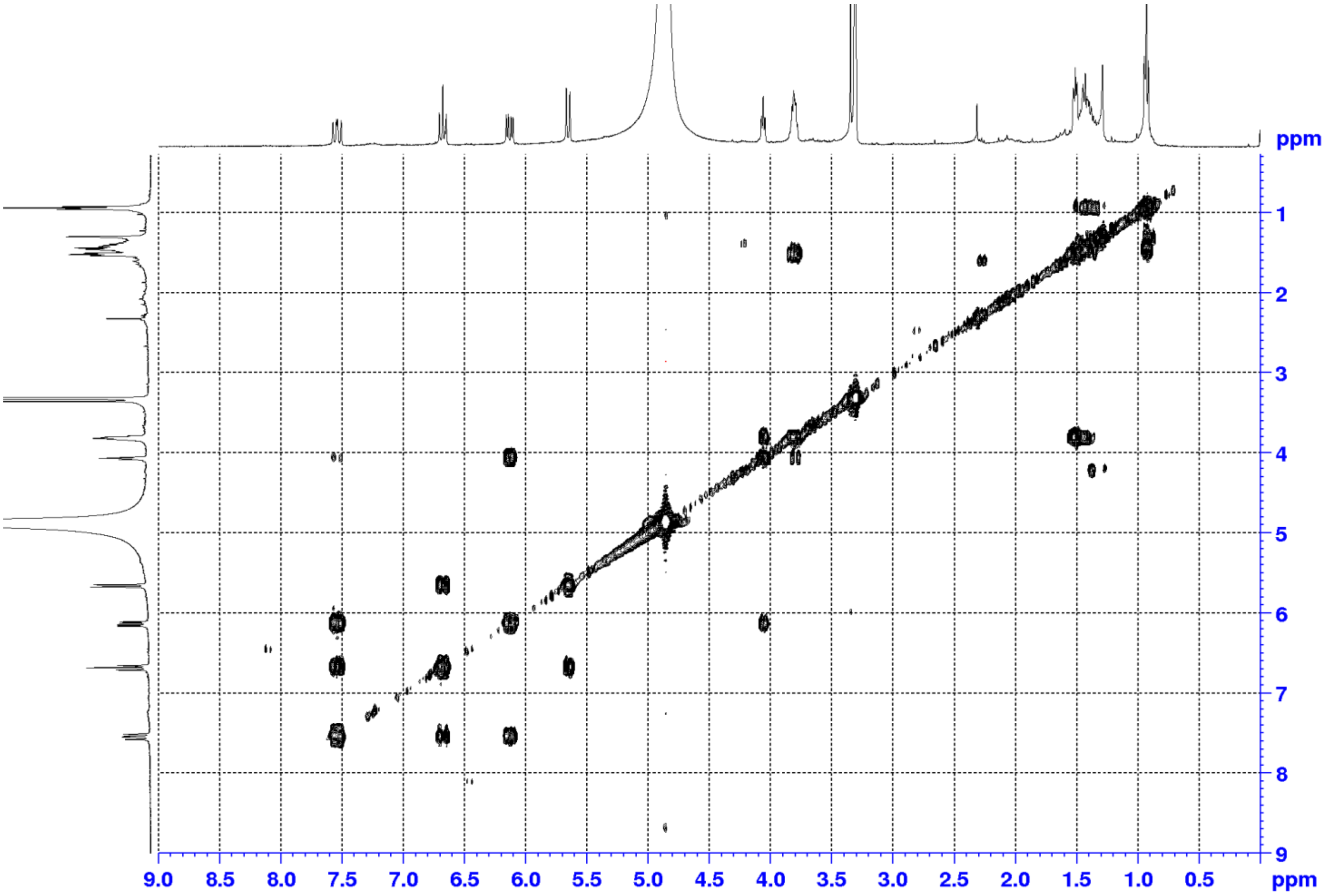

Figure S50: COSY spectrum of **12** in methanol-d_4

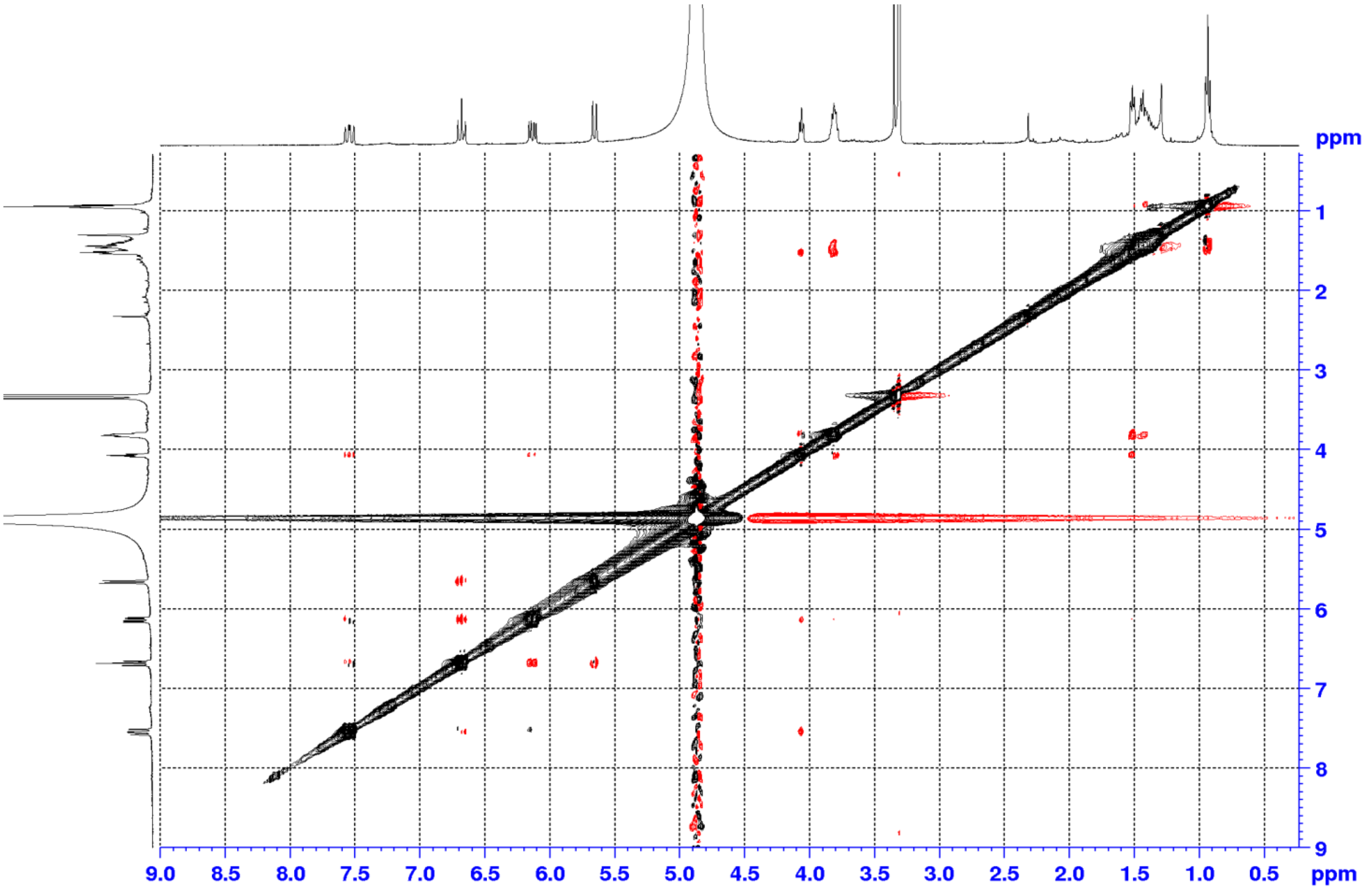

Figure S51: NOESY spectrum of **12** in methanol-d_4

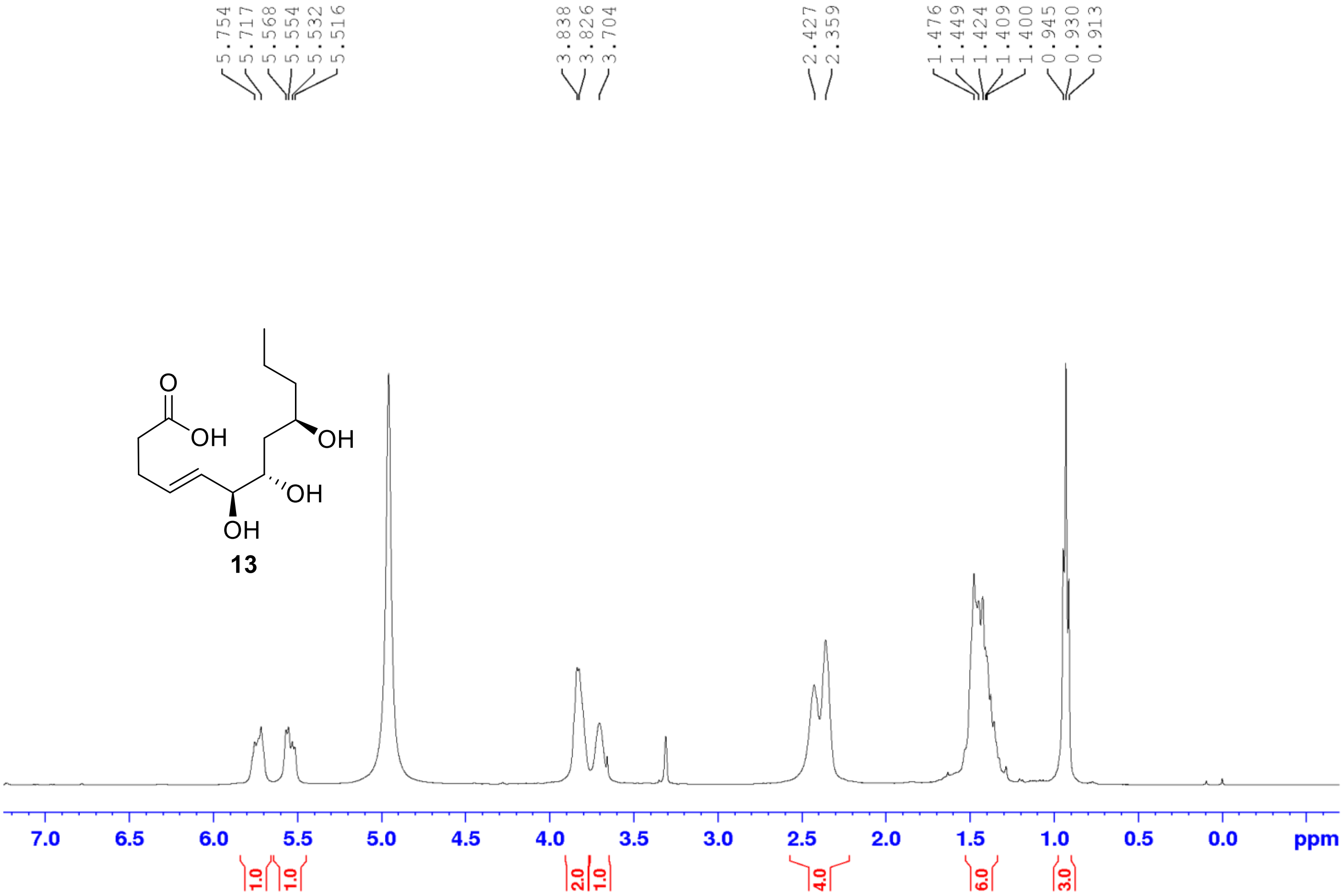

Figure S52: ^{1}H NMR spectrum of **13** in methanol-d_4 (400 MHz)

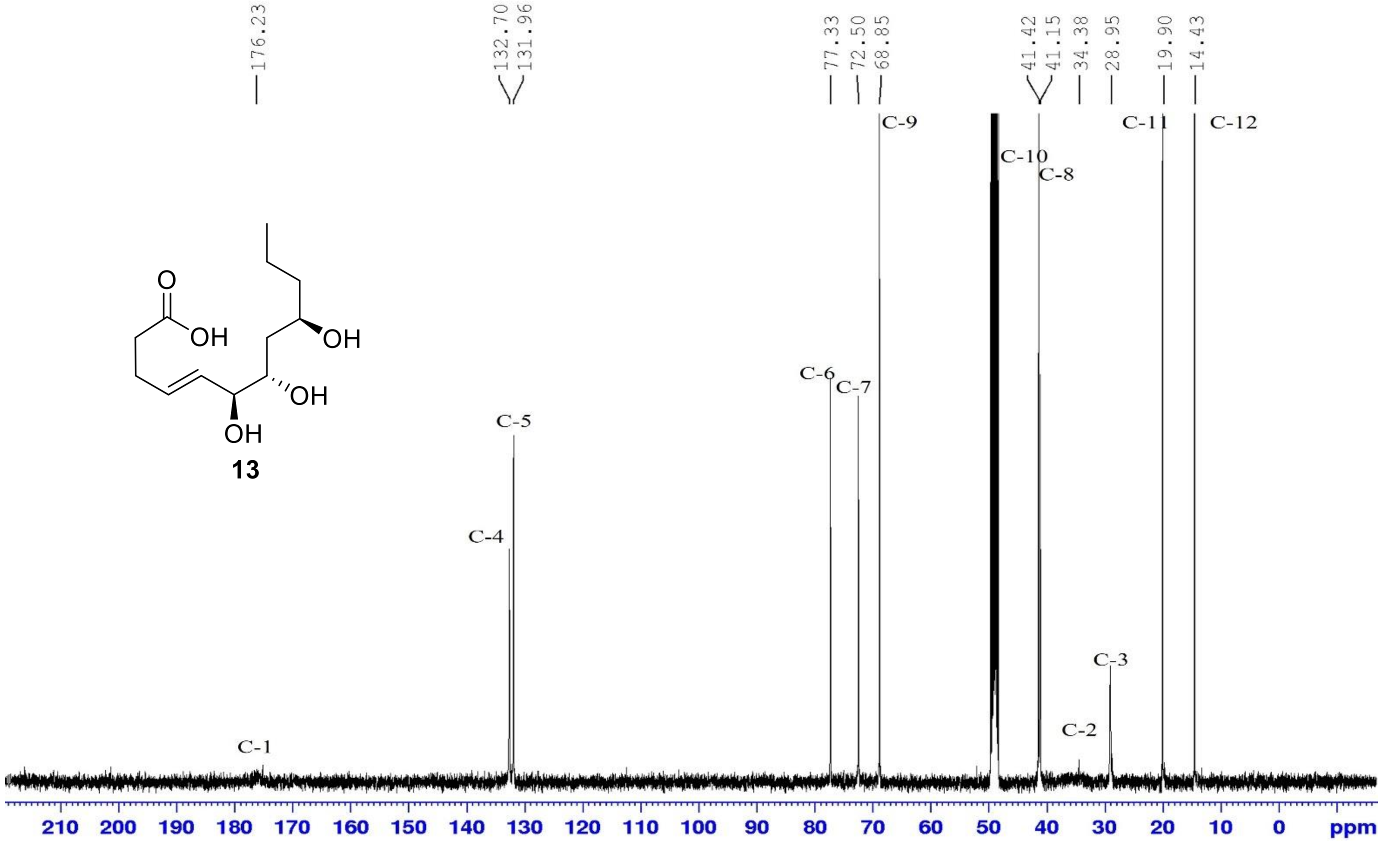

Figure S53: ^{13}C NMR spectrum of **13** in methanol-d_4 (101 MHz)

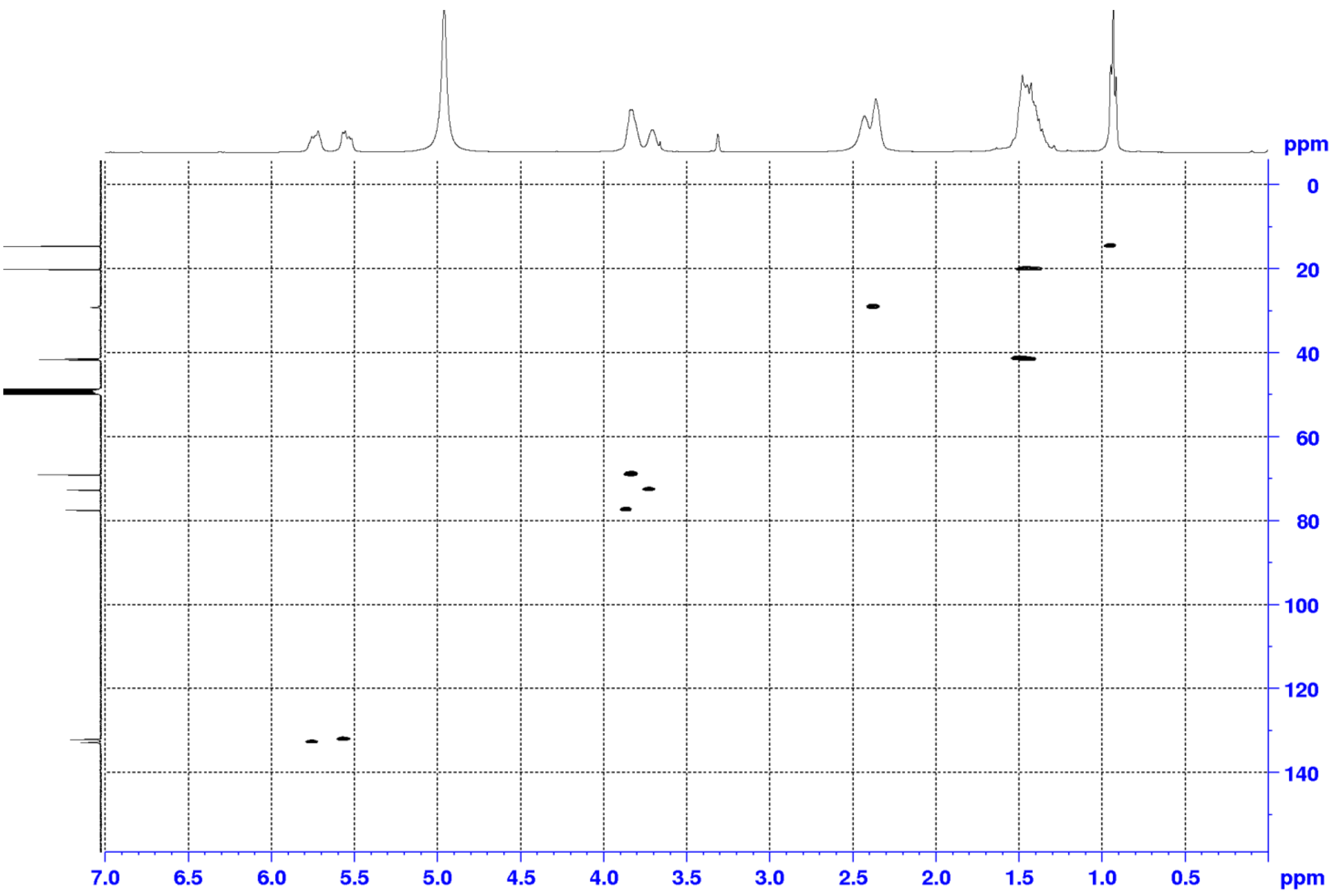

Figure S54: HSQC spectrum of **13** in methanol-d_4

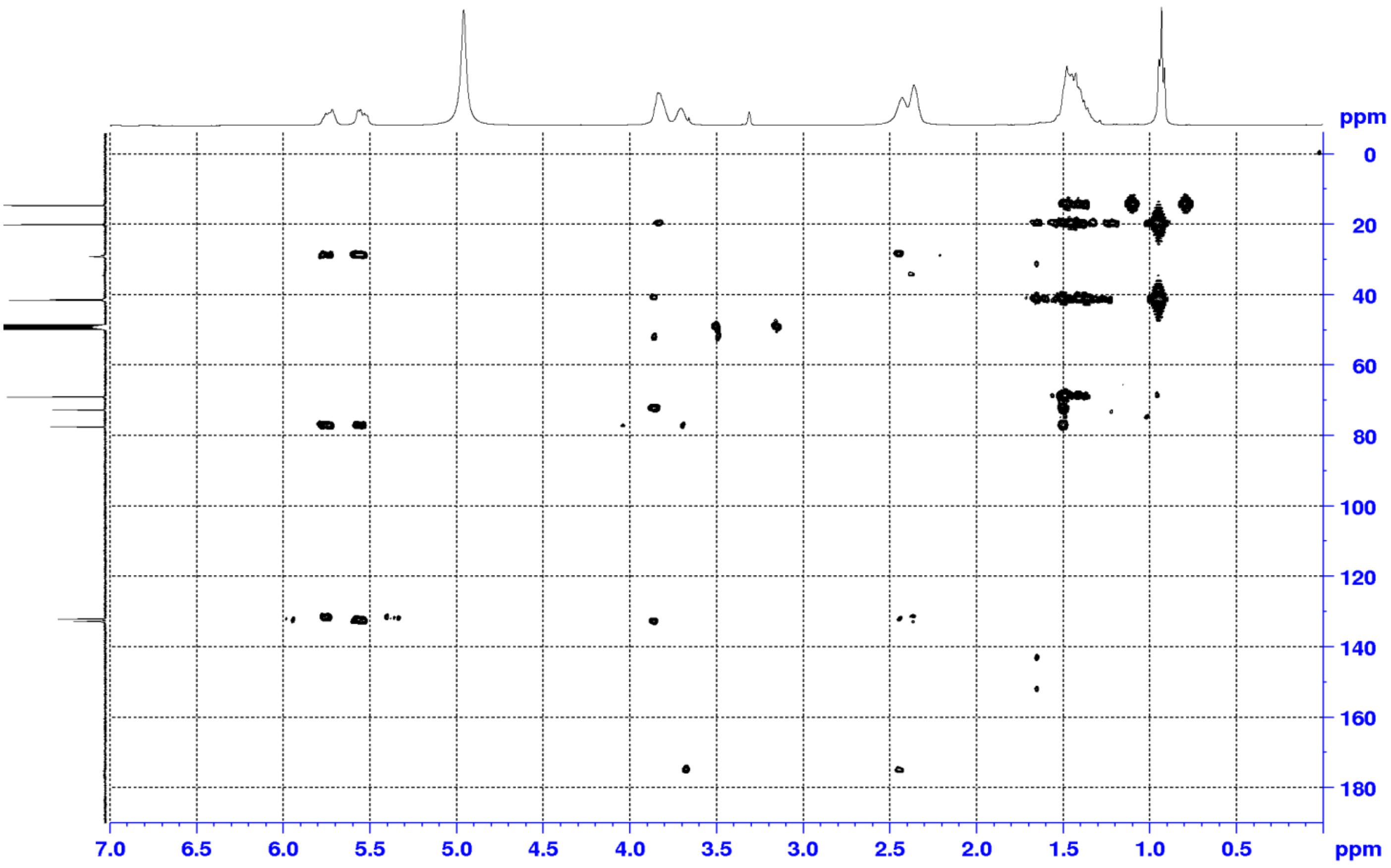

Figure S55: HMBC spectrum of **13** in methanol-d_4

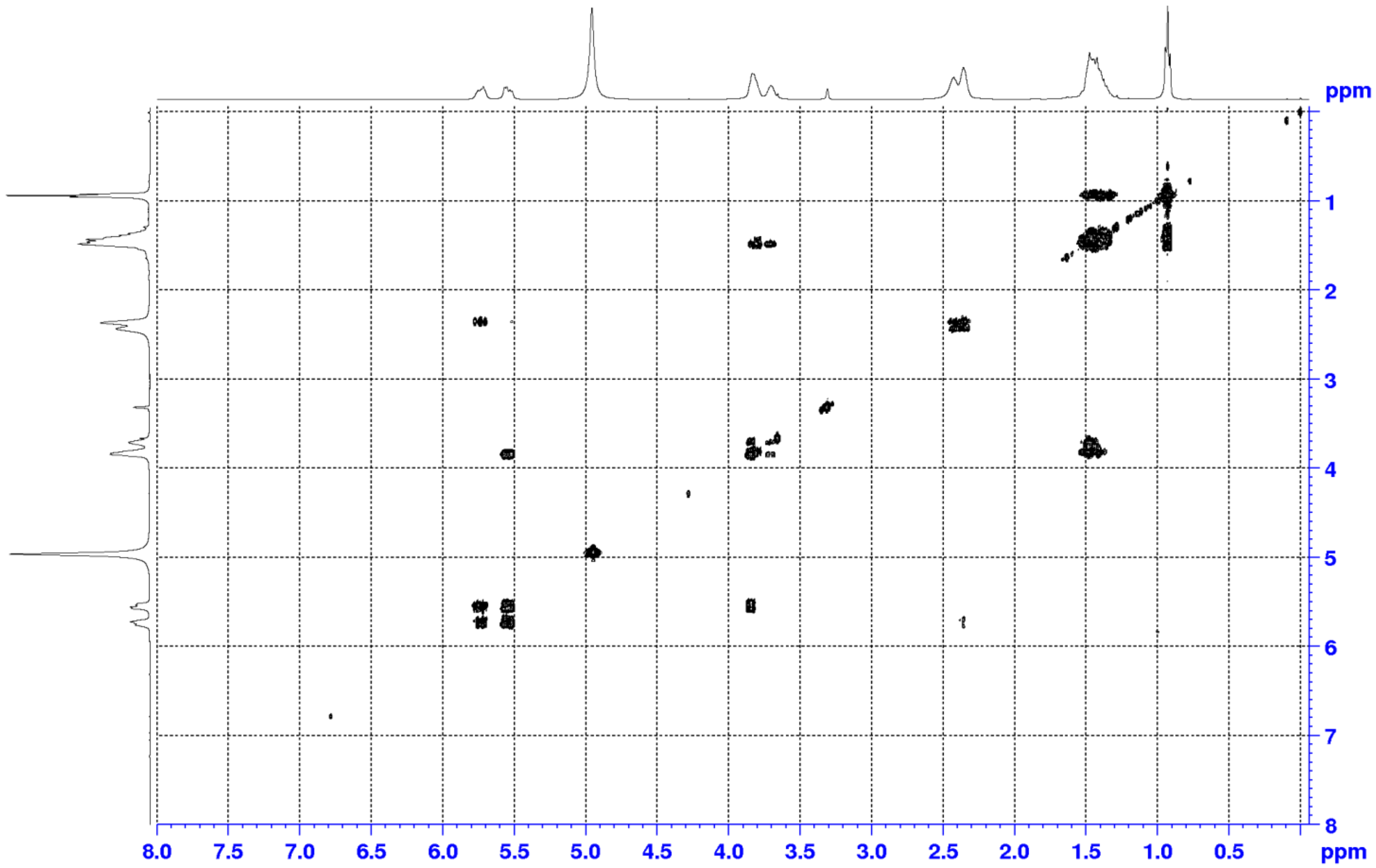

Figure S56: COSY spectrum of **13** in methanol-d_4

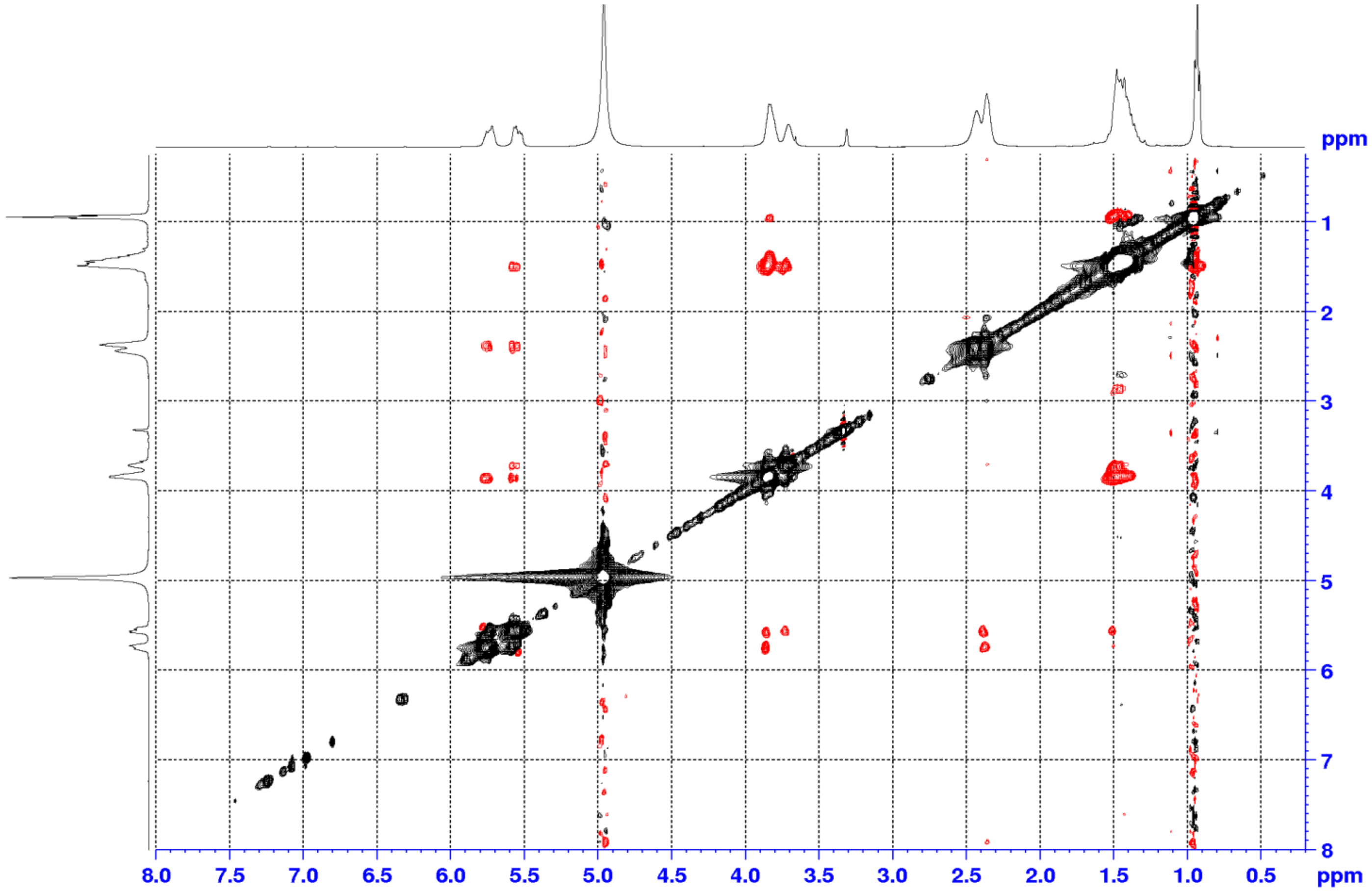

Figure S57: NOESY spectrum of **13** in methanol-d_4

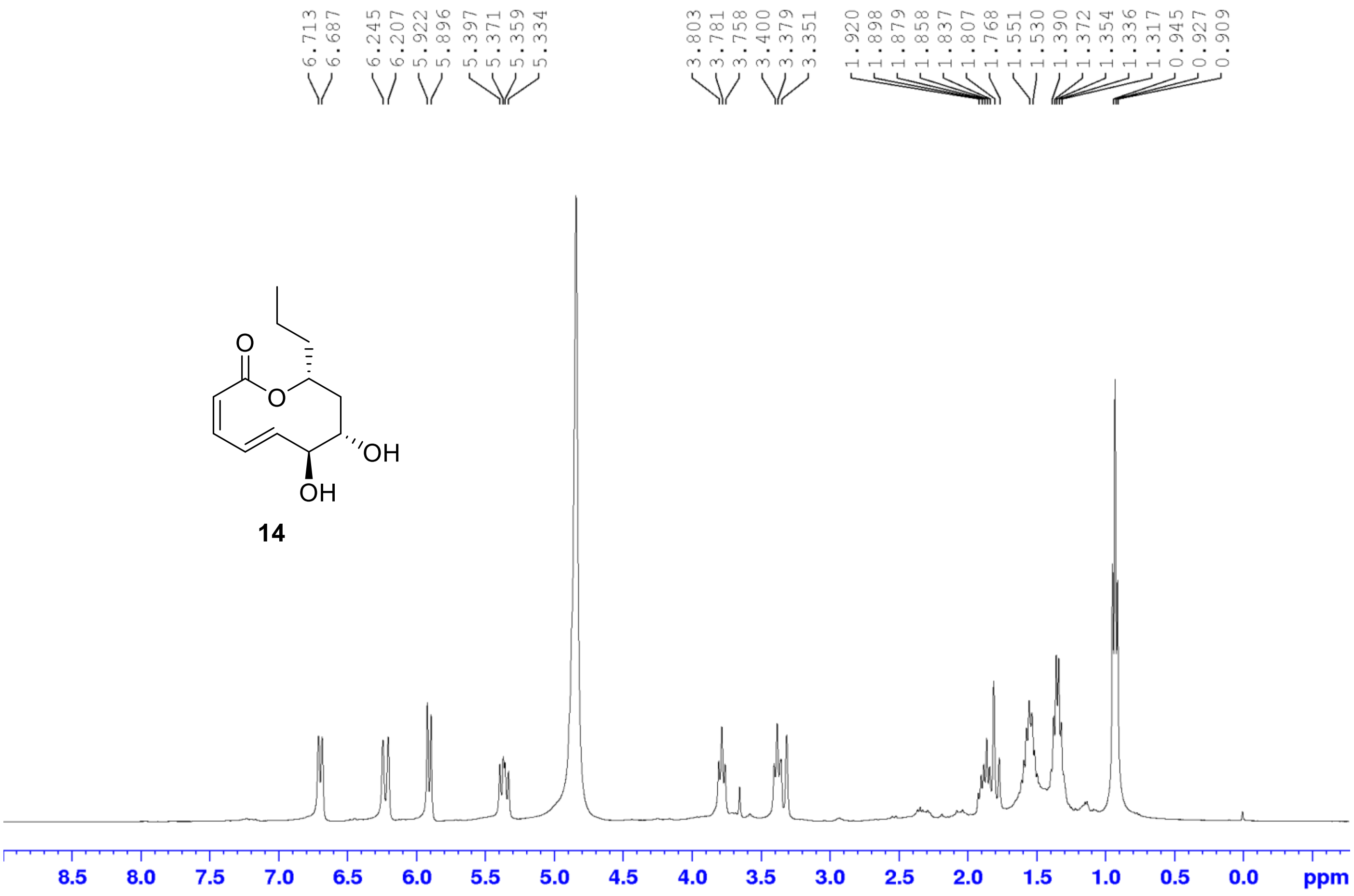

Figure S58: ^{1}H NMR spectrum of **14** in methanol-d_4 (400 MHz)

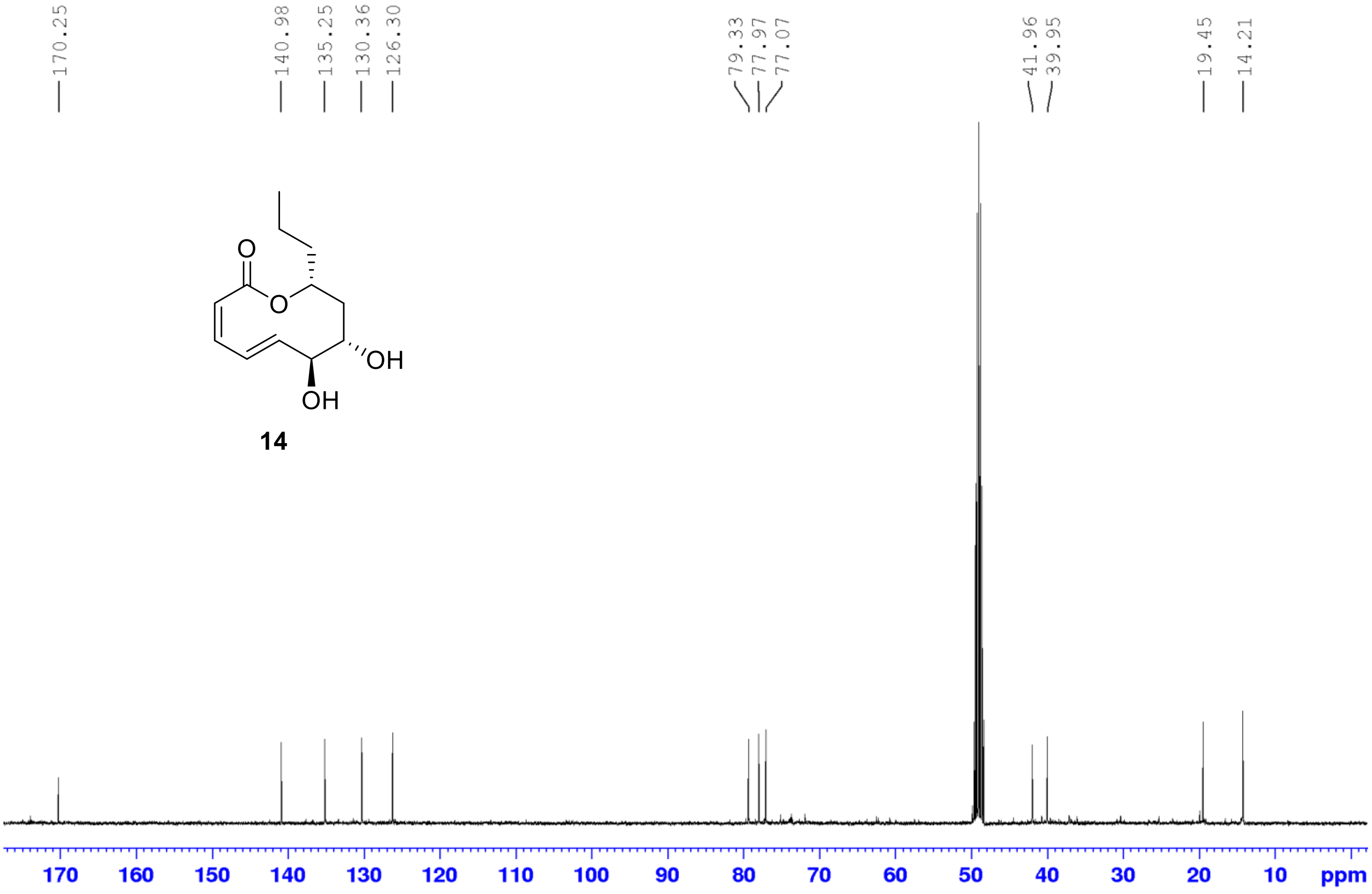

Figure S59: ^{13}C NMR spectrum of **14** in methanol-d_4 (101 MHz)

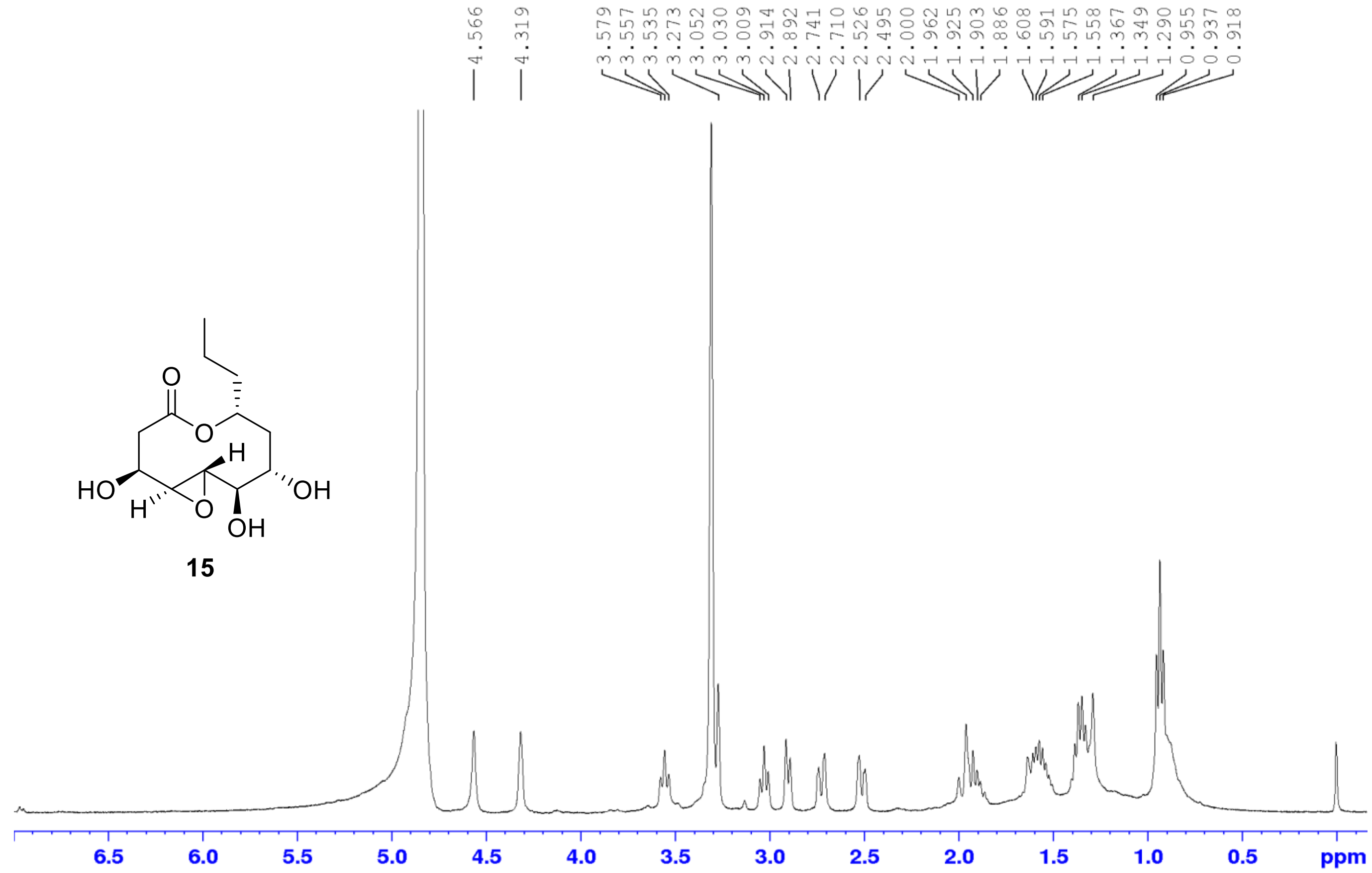

Figure S60: ^{1}H NMR spectrum of **15** in methanol-d_4 (400 MHz)

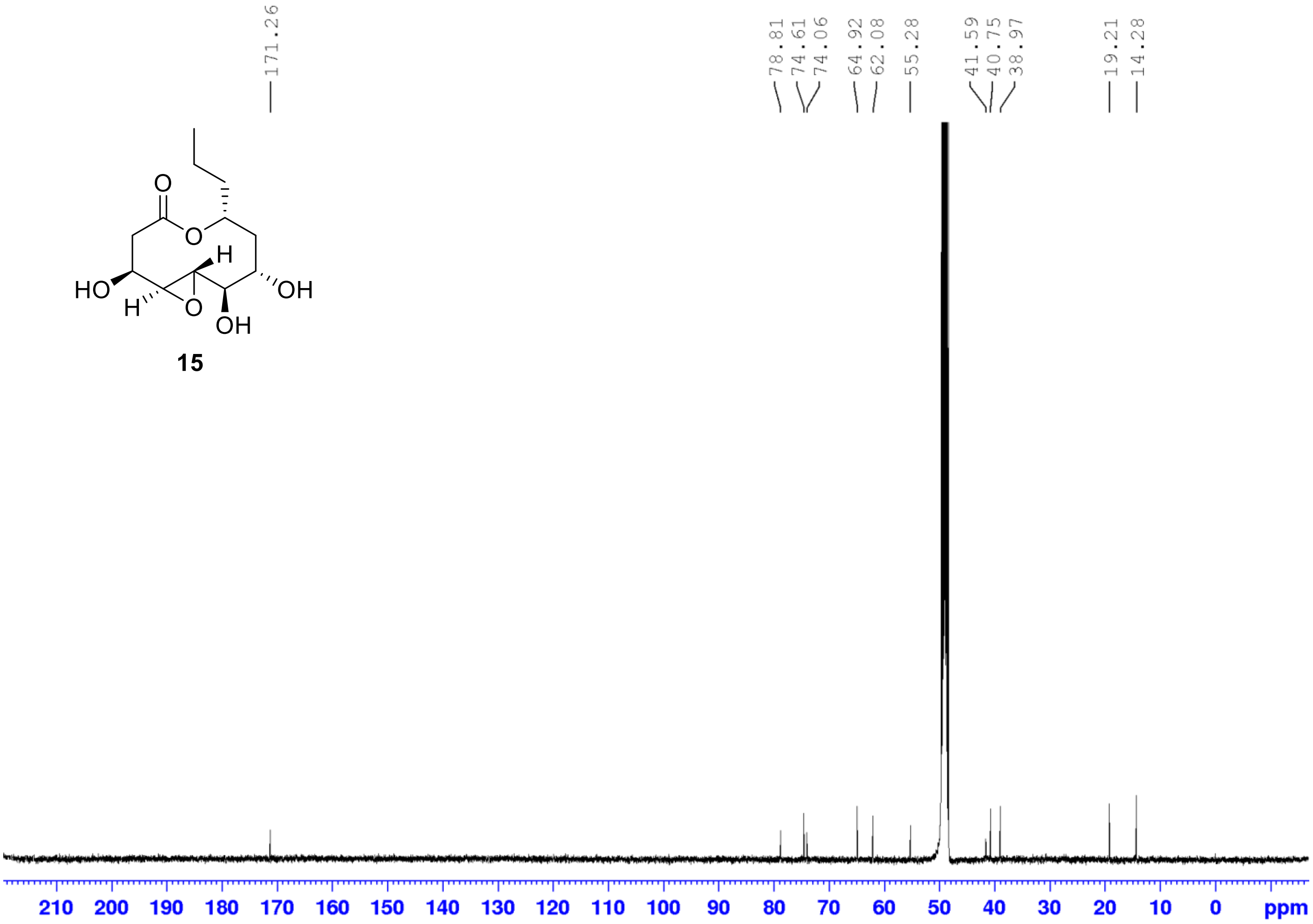

Figure S61: ^{13}C NMR spectrum of **15** in methanol-d_4 (101 MHz)

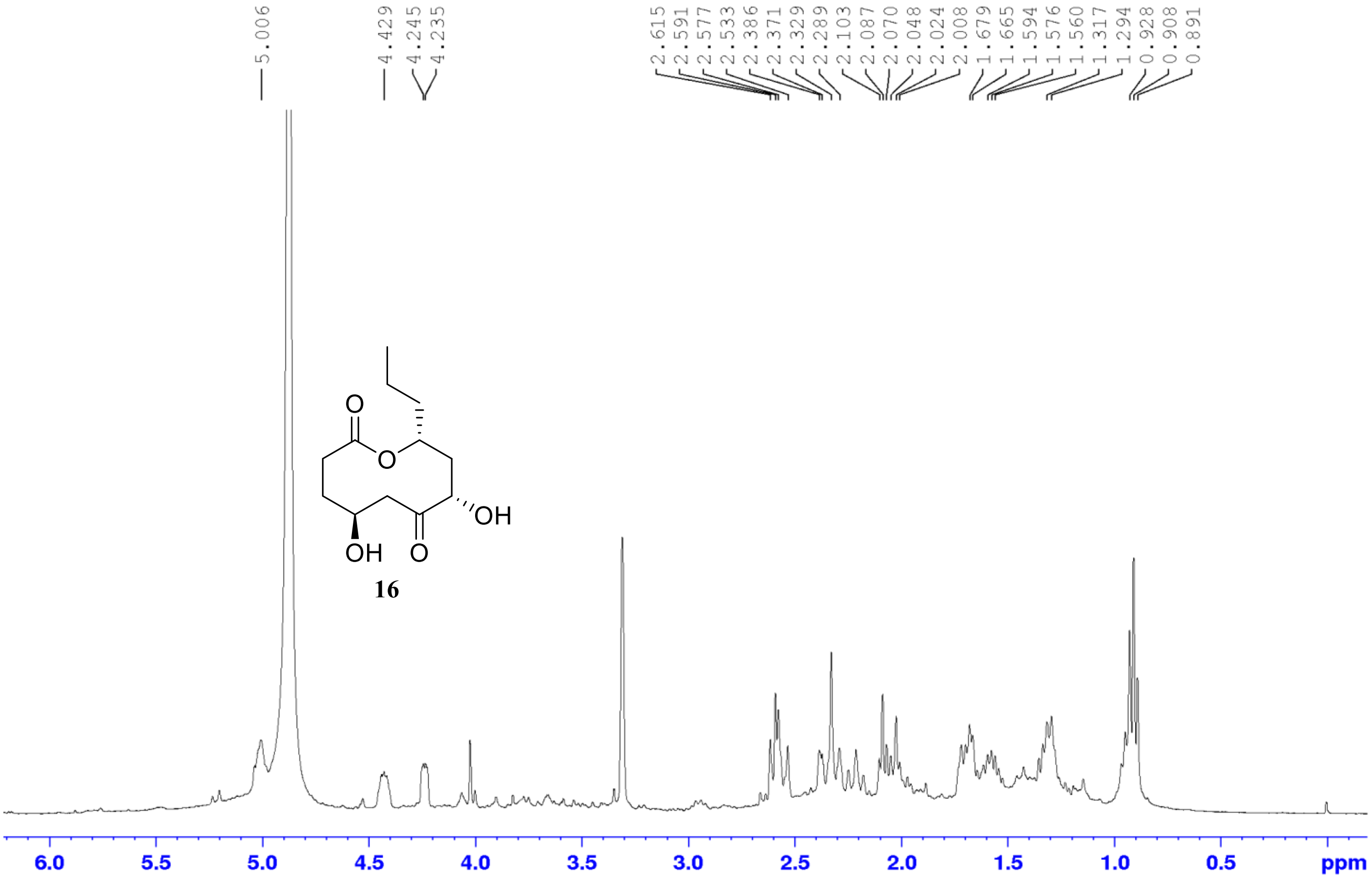

Figure S62: ^{1}H NMR spectrum of **16** in methanol-d_4 (400 MHz)

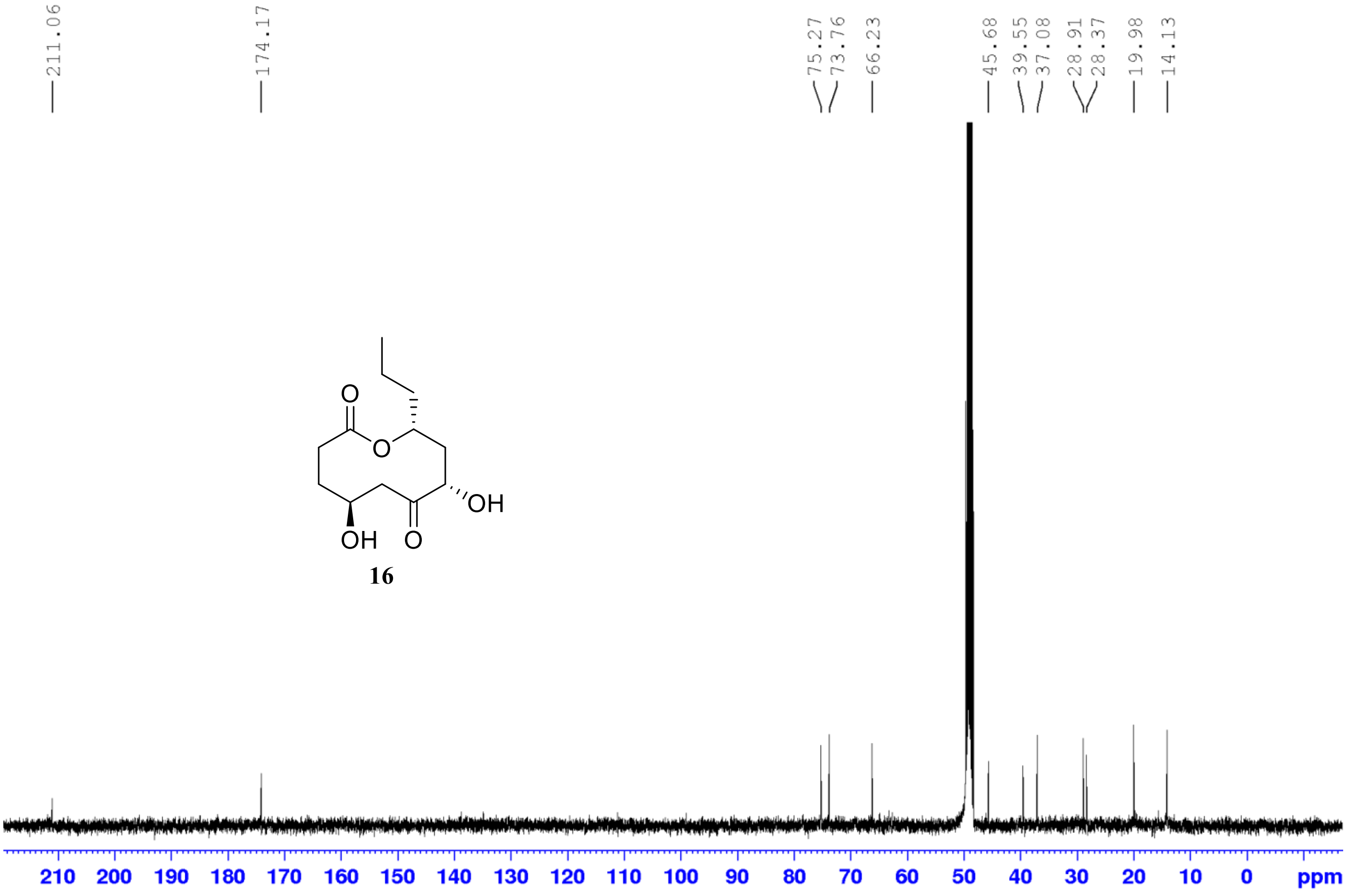

Figure S63: ^{13}C NMR spectrum of **16** in methanol-d_4 (101 MHz)

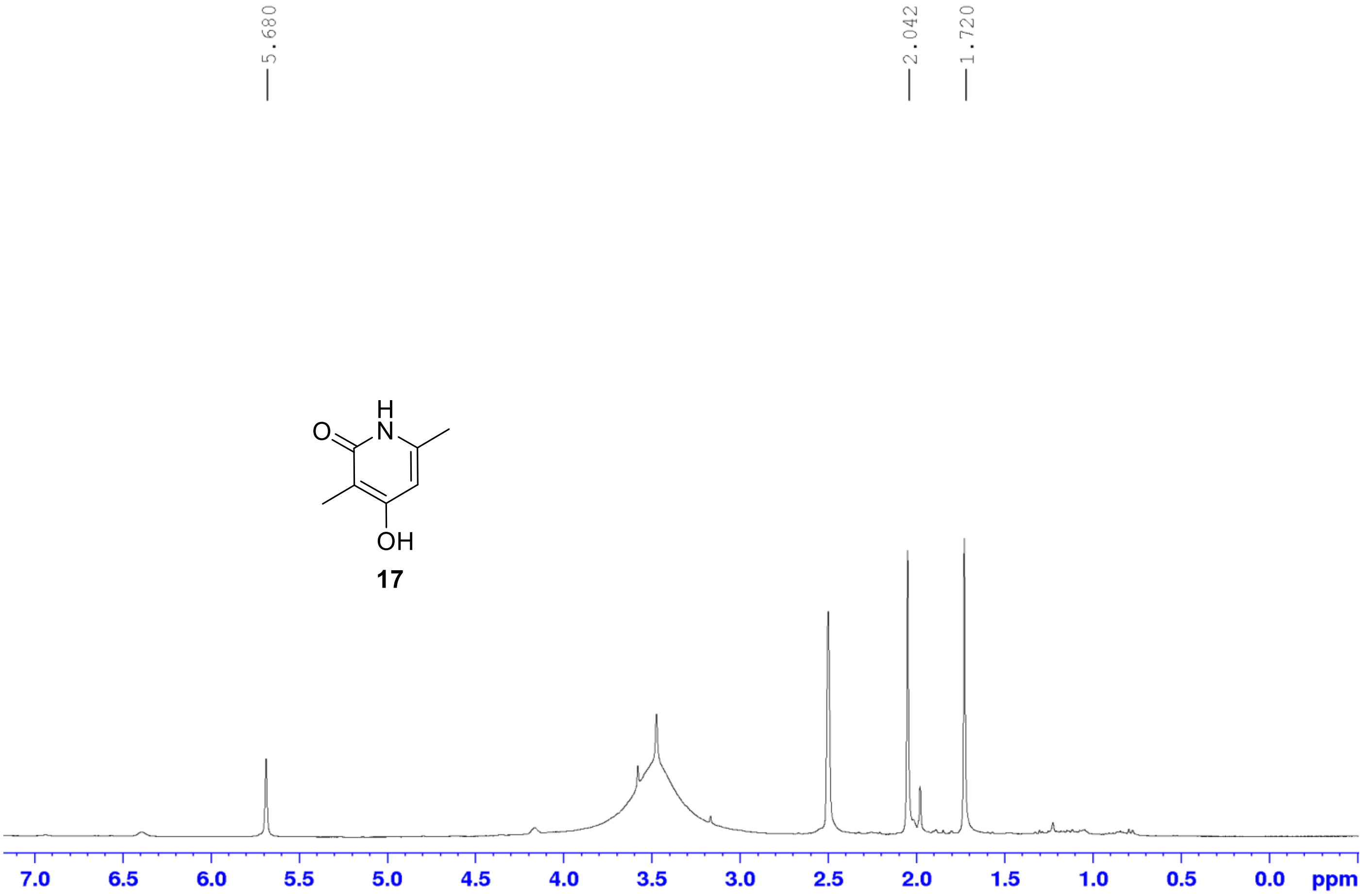

Figure S64: ^{1}H NMR spectrum of **17** in DMSO-d_6 (400 MHz)

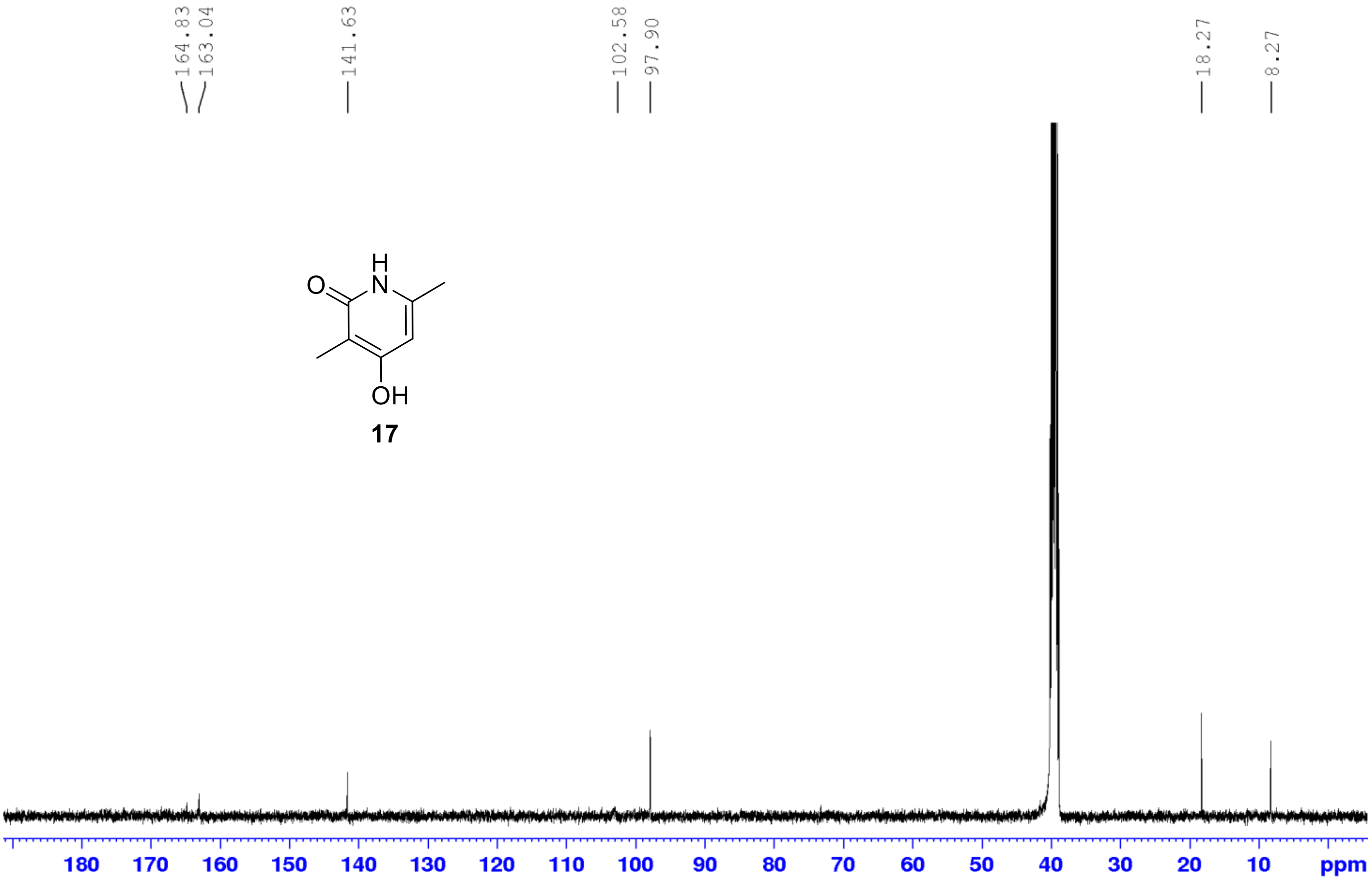

Figure S65: ^{13}C NMR spectrum of **17** in DMSO-d_6 (101 MHz)

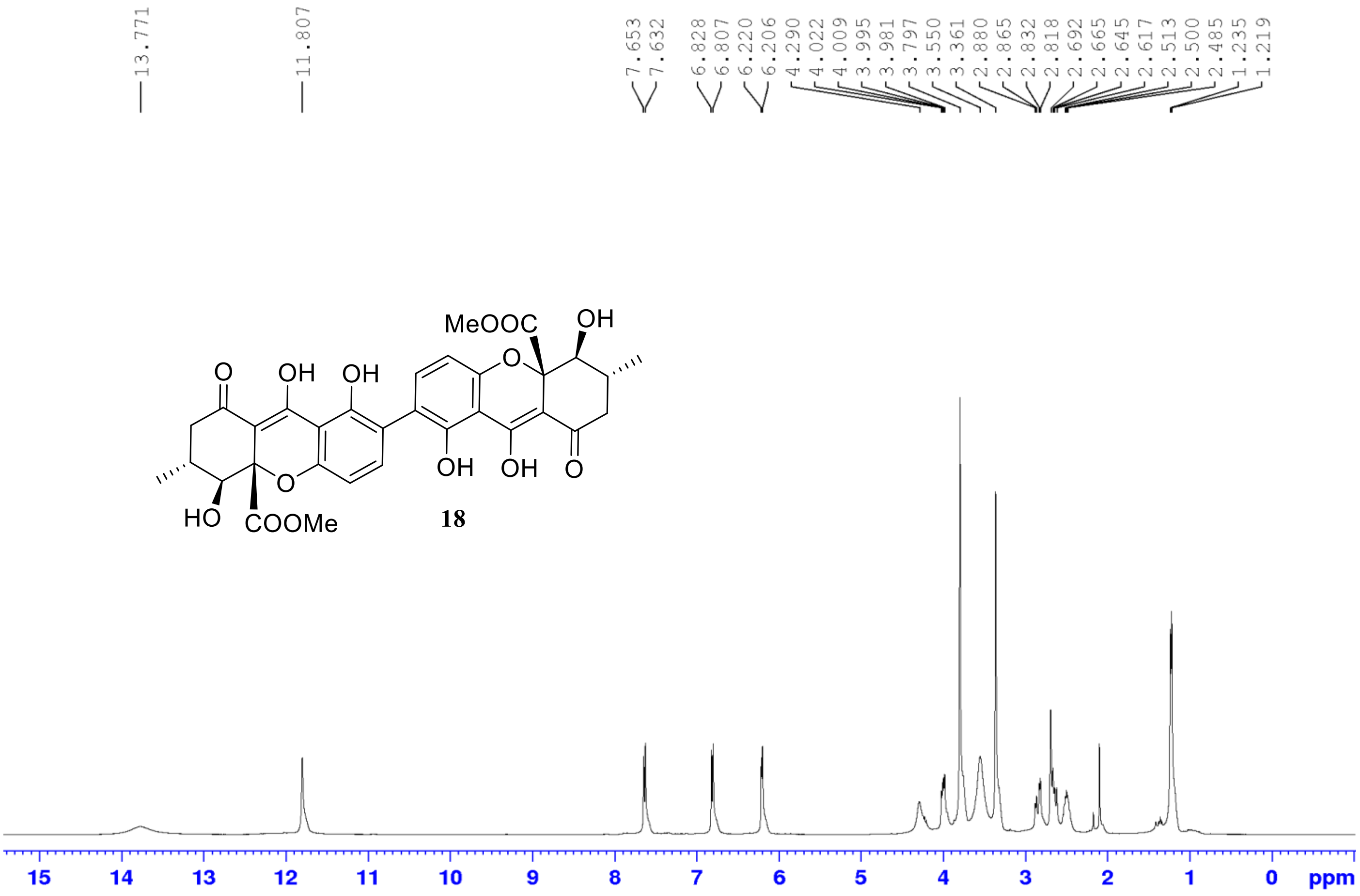

Figure S66: ^{1}H NMR spectrum of **18** in DMSO-d_6 (400 MHz)

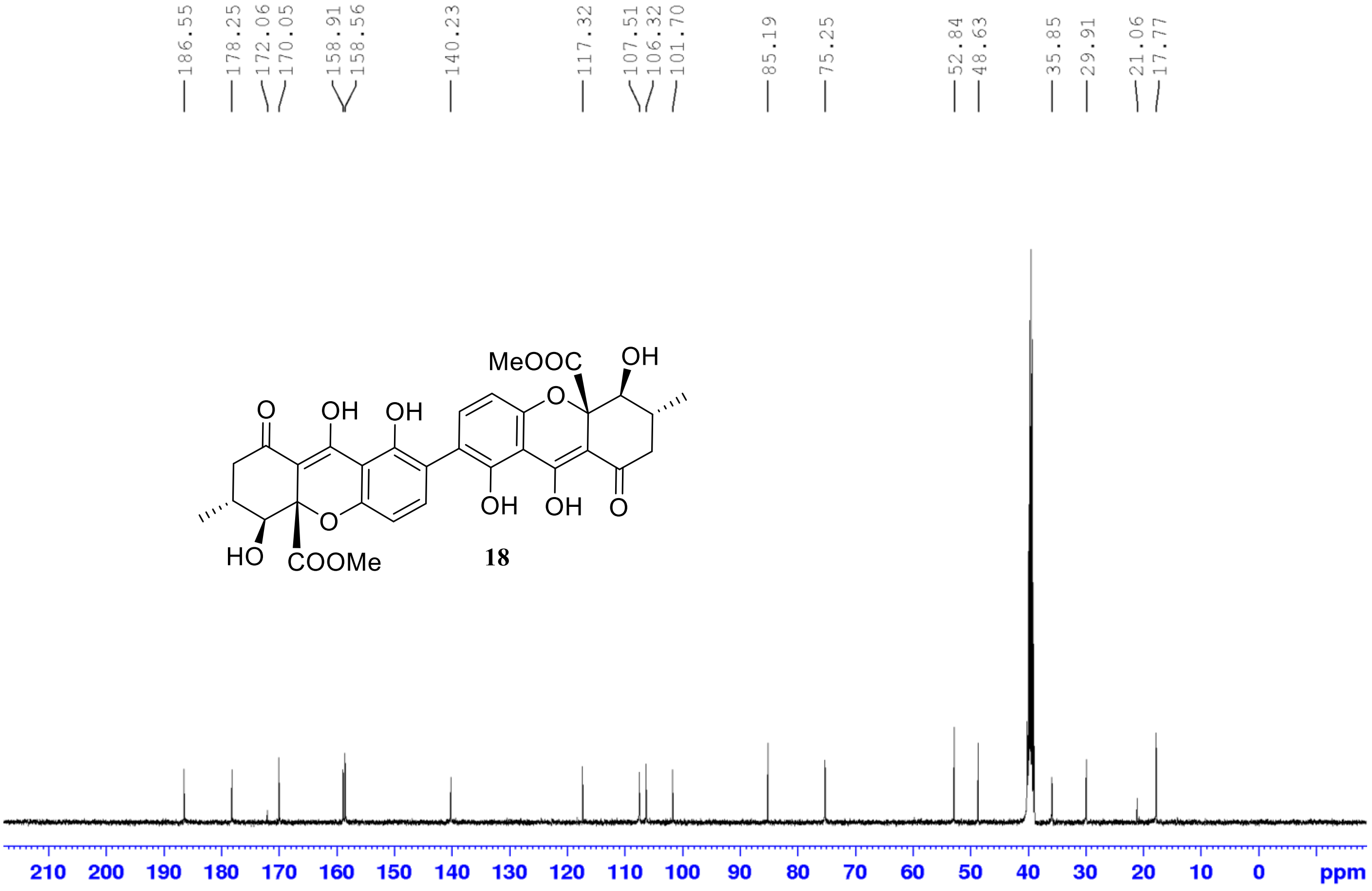

Figure S67: ^{13}C NMR spectrum of **18** in DMSO-d_6 (101 MHz)

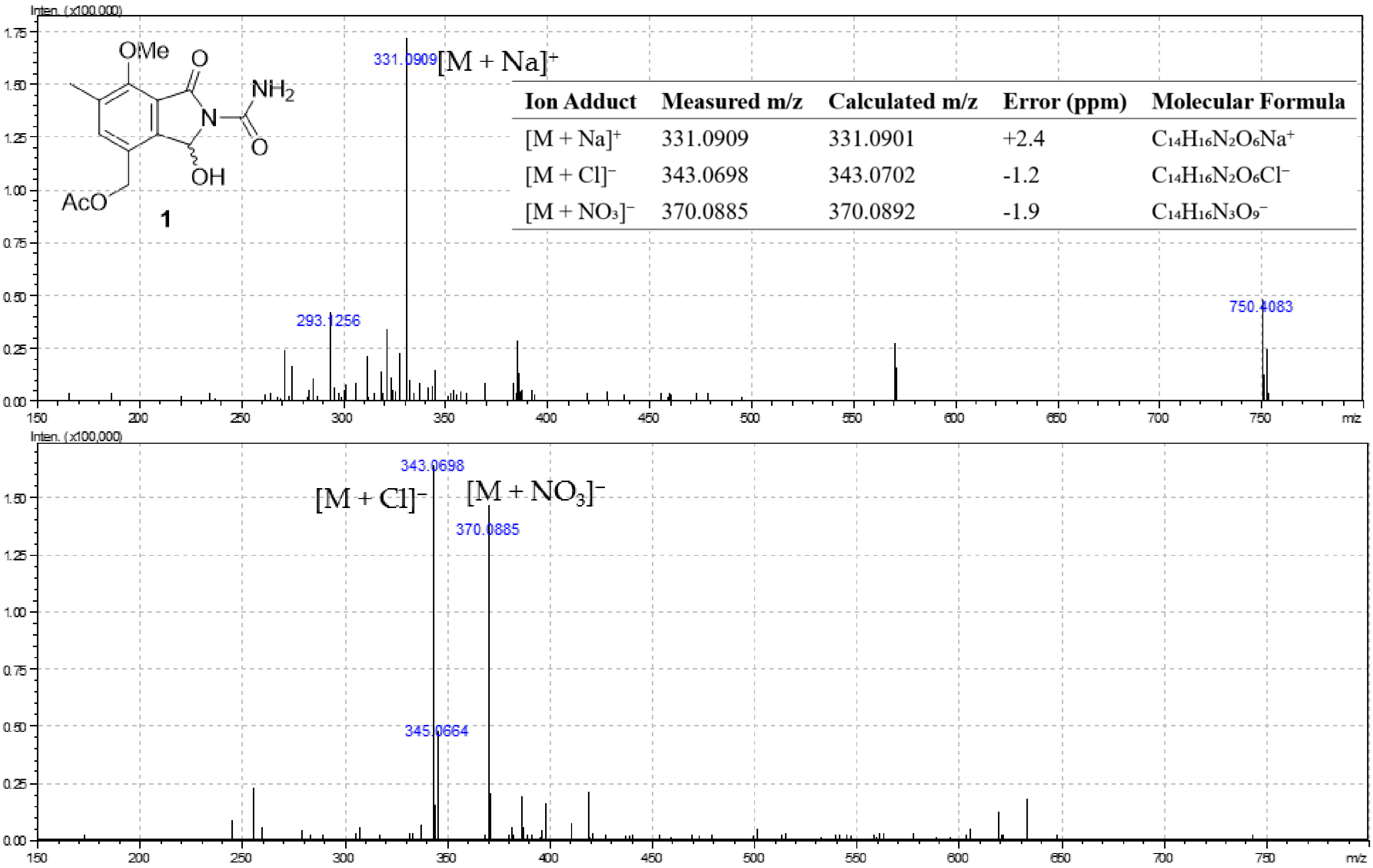

Ion Adduct	Measured m/z	Calculated m/z	Error (ppm)	Molecular Formula
$[M + Na]^+$	331.0909	331.0901	+2.4	$C_{14}H_{16}N_2O_6Na^+$
$[M + Cl]^-$	343.0698	343.0702	-1.2	$C_{14}H_{16}N_2O_6Cl^-$
$[M + NO_3]^-$	370.0885	370.0892	-1.9	$C_{14}H_{16}N_3O_9^-$

Figure S68: HRESIMS spectrum of compound **1**

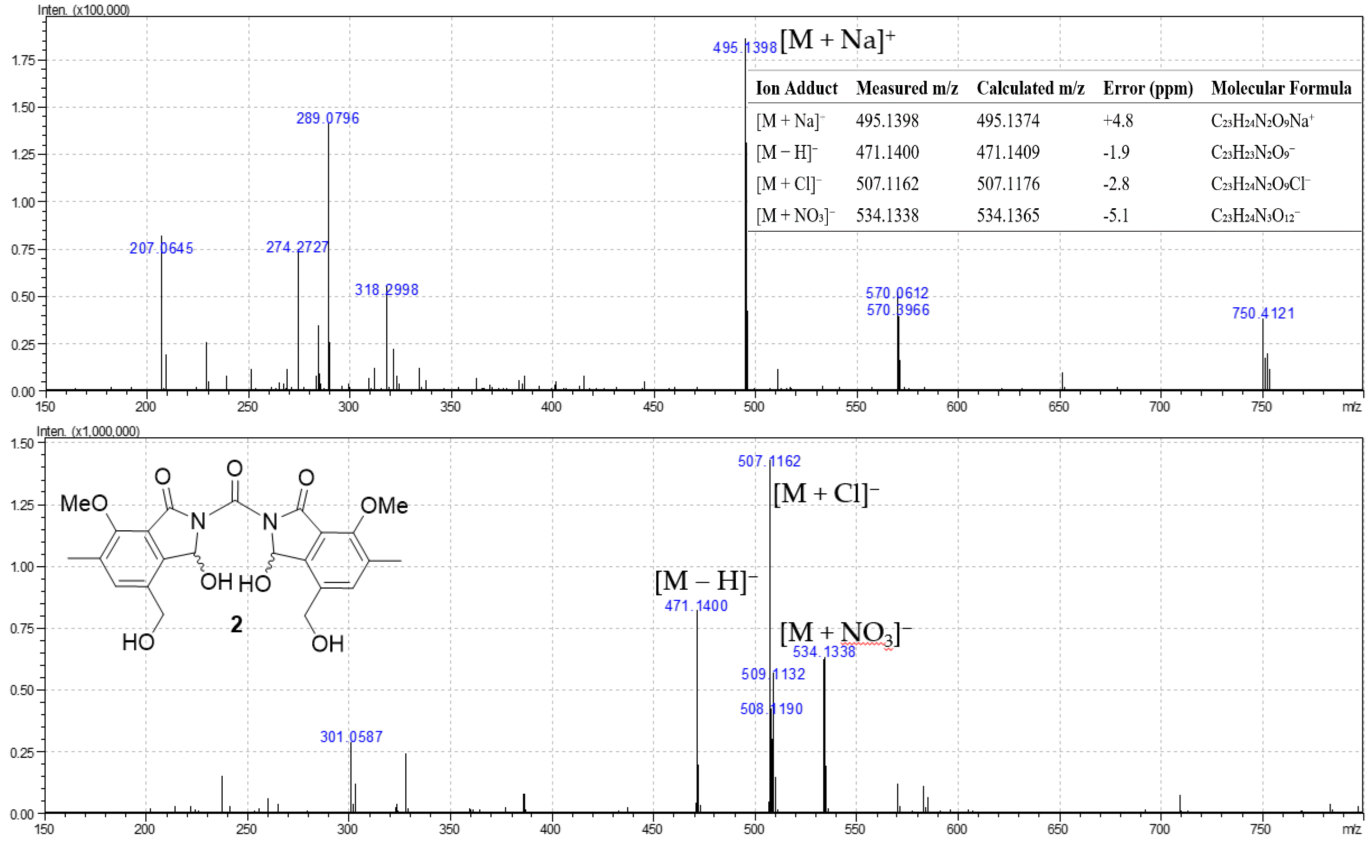

Ion Adduct	Measured m/z	Calculated m/z	Error (ppm)	Molecular Formula
$[M + Na]^+$	495.1398	495.1374	+4.8	$C_{23}H_{24}N_2O_9Na^+$
$[M - H]^-$	471.1400	471.1409	-1.9	$C_{23}H_{23}N_2O_9^-$
$[M + Cl]^-$	507.1162	507.1176	-2.8	$C_{23}H_{24}N_2O_9Cl^-$
$[M + NO_3]^-$	534.1338	534.1365	-5.1	$C_{23}H_{24}N_3O_{12}^-$

Figure S69: HRESIMS spectrum of compound **2**

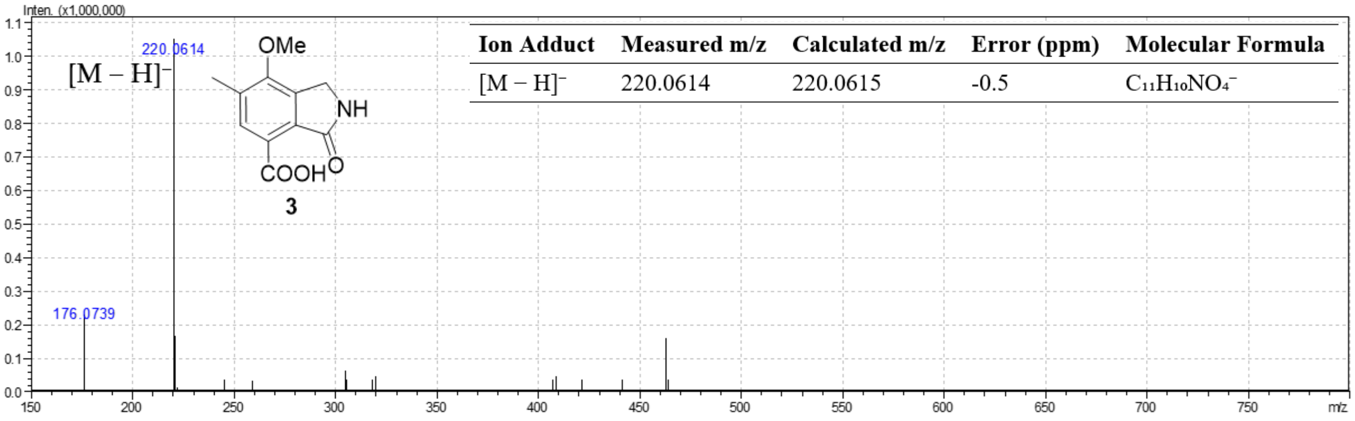

Ion Adduct	Measured m/z	Calculated m/z	Error (ppm)	Molecular Formula
[M – H]⁻	220.0614	220.0615	-0.5	$C_{11}H_{10}NO_4^-$

Figure S70: HRESIMS spectrum of compound **3**

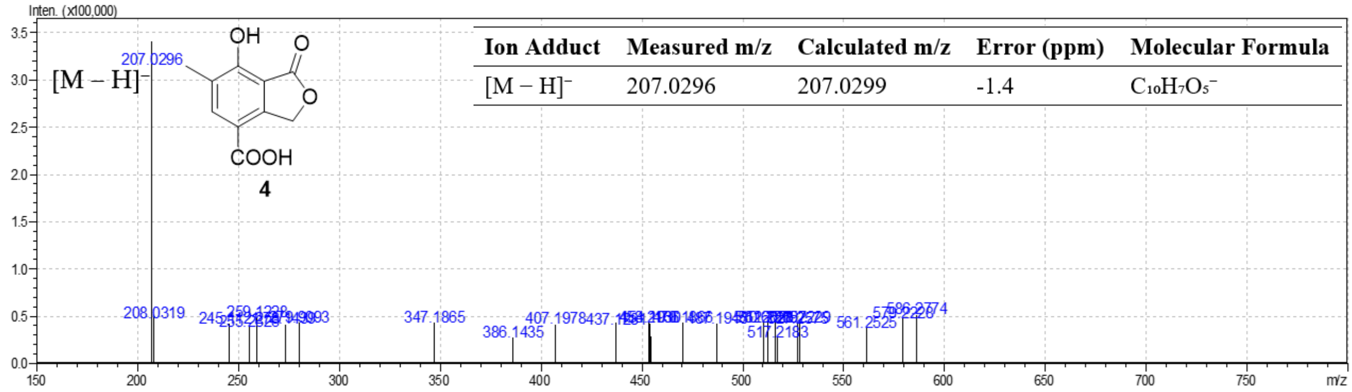

Ion Adduct	Measured m/z	Calculated m/z	Error (ppm)	Molecular Formula
[M – H]⁻	207.0296	207.0299	-1.4	$C_{10}H_7O_5^-$

Figure S71: HRESIMS spectrum of compound **4**

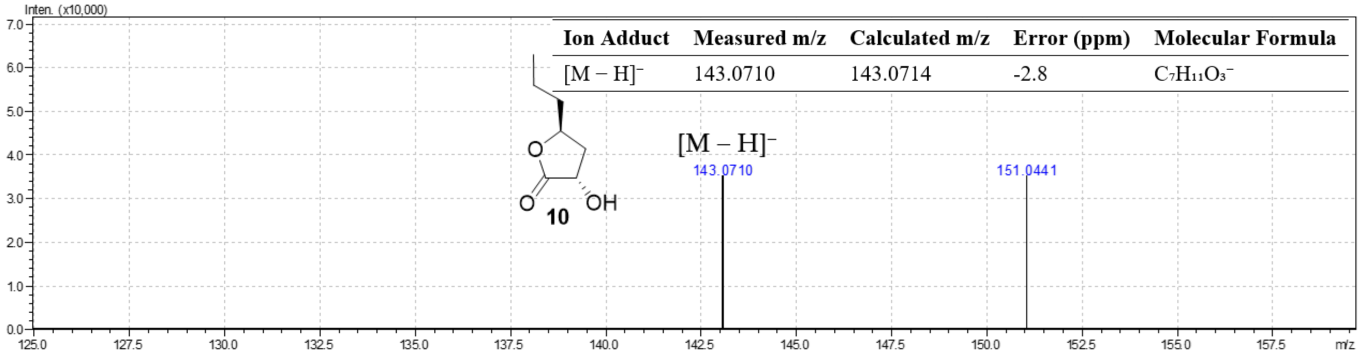

Ion Adduct	Measured m/z	Calculated m/z	Error (ppm)	Molecular Formula
[M – H]⁻	143.0710	143.0714	-2.8	$C_7H_{11}O_3^-$

Figure S72: HRESIMS spectrum of compound **10**

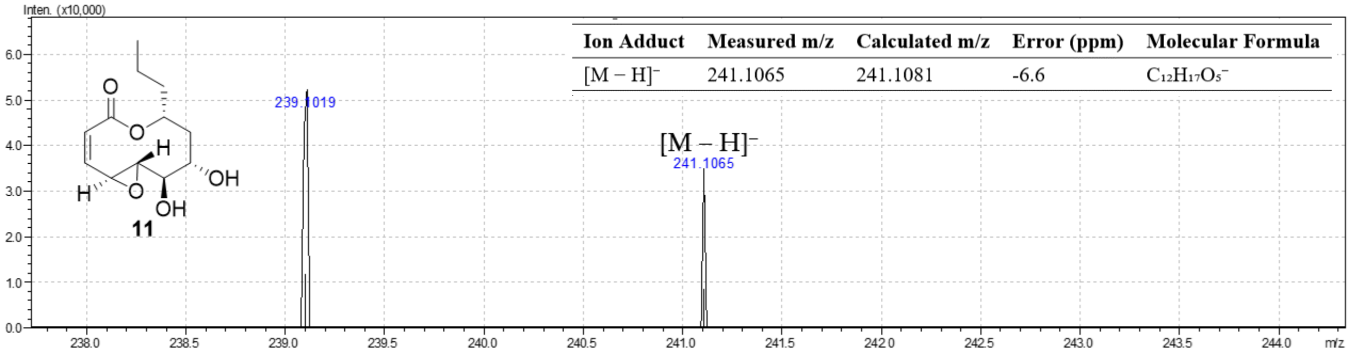

Ion Adduct	Measured m/z	Calculated m/z	Error (ppm)	Molecular Formula
[M – H]⁻	241.1065	241.1081	-6.6	$C_{12}H_{17}O_5^-$

Figure S73: HRESIMS spectrum of compound **11**

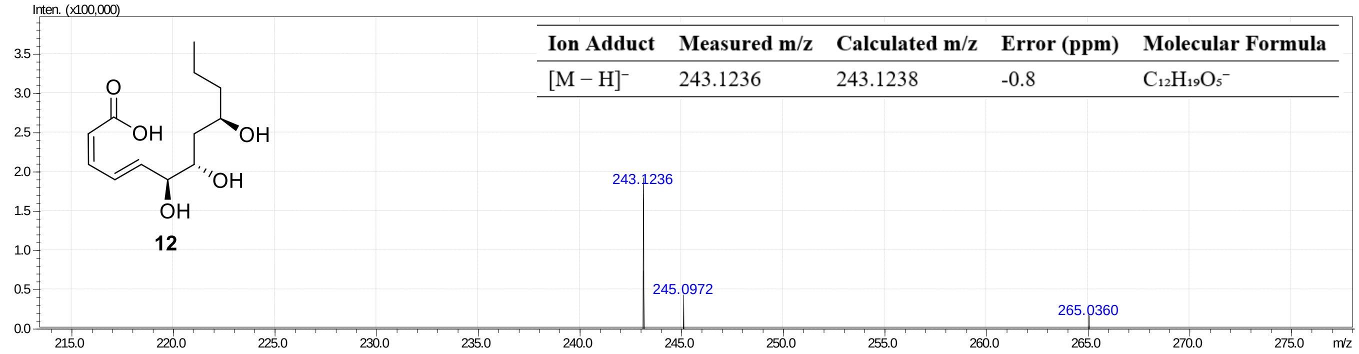

Figure S74: HRESIMS spectrum of compound **12**

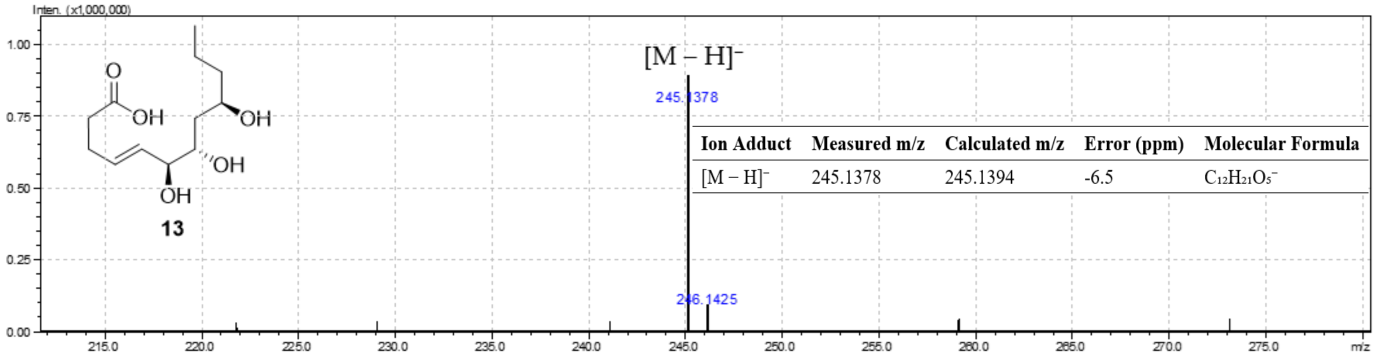

Figure S75: HRESIMS spectrum of compound **13**

Table S1: Shielding sensors (σ_C) of the conformers (**1**) and the calculated chemical shifts (δ_C).

Nulei	C1 (27.08%)	C2(24.92%)	C3 (22.57%)	C4 (12.56%)	C4 (9.5%)	C5 (3.38%)	Averaged	Exp.	Cal.
1	14.5229	14.0493	14.1444	14.4878	13.8615	14.9808	14.26913918	166.3	**165.9**
3	103.4674	102.8195	102.3693	103.6366	102.6052	103.3344	103.003296	81.5	**79.9**
4	37.5814	37.673	38.3055	42.4783	37.1798	37.9593	38.36108589	144.3	**142.7**
5	55.1883	55.5454	54.0074	53.8783	54.27	53.2436	54.69877366	126.4	**126.9**
6	42.0821	42.5671	42.497	47.2594	38.8811	40.7261	42.60115422	137.4	**138.6**
7	44.8547	45.8411	45.7944	45.1522	46.0328	45.1779	45.4772963	132.6	**135.8**
8	21.9734	23.4139	24.055	23.8604	22.6688	22.9509	23.14049676	156.0	**157.4**
9	61.2331	61.1459	61.5907	62.0112	61.1575	60.9942	61.38067593	118.3	**120.3**
10	119.7189	119.3204	123.663	121.2852	124.069	124.4499	121.2916436	61.4	**62.0**
11	168.0954	168.0131	167.9653	167.6869	168.1897	167.9985	168.0167125	15.2	**16.9**
12	27.5048	27.2354	27.2969	27.4547	27.1057	27.4791	27.34841725	156.9	**153.3**
13	125.7229	125.274	125.4143	125.9805	125.1665	125.3704	125.5215375	61.7	**58.1**
14	7.0643	7.0016	6.986	7.3657	6.2637	7.4918	7.00795762	172.5	**172.9**
15	164.5276	164.637	164.386	164.3624	164.3394	163.5152	164.4665089	20.4	**20.4**

Table S2: Shielding sensors (σ_C) of the conformer (**2**) and the calculated chemical shifts (δ_C).

Nulei	Shielding Tensor	Exp.	Cal.
1	11.0819	166.5	172.2
3	102.8185	80.8	78.2
4	39.0086	142.2	143.6
5	49.946	132.8	132.4
6	42.8347	135.5	139.7
7	45.8711	132.4	136.6
8	23.1348	154.8	159.9
9	63.4368	117.8	118.6
10	121.0413	58.4	59.6
11	168.2664	15.2	11.2
12	28.4908	155.2	154.4
OMe	125.7449	61.6	54.7

Table S3: Shielding sensors (σ_C) of the conformer (**10**) and the calculated chemical shifts (δ_C).

Conformer	C4 (56.51%)	C2 (22.01%)	C1 (16.68%)	C3 (4.8%)	averaged	Exp.	Cal.
1	-1.268	-0.9034	-1.2355	-1.0218	-1.17	179.4	180.3
2	105.0329	105.163	109.2576	107.4191	105.88	78.1	78.2
3	117.4663	117.7509	117.4897	117.7673	117.55	69.5	67.0
4	146.3207	148.3654	148.3508	150.2247	147.30	38.6	38.6
5	147.963	150.7758	148.3508	151.7343	148.83	38.7	37.2
6	163.6234	164.9224	166.0673	165.9435	164.43	19.5	22.3
7	172.2806	172.1559	175.202	174.3137	172.8380319	14.1	14.3
